# Co-existing TP53 and ARID1A mutations promote aggressive endometrial tumorigenesis

**DOI:** 10.1371/journal.pgen.1009986

**Published:** 2021-12-23

**Authors:** Jake J. Reske, Mike R. Wilson, Jeanne Holladay, Rebecca A. Siwicki, Hilary Skalski, Shannon Harkins, Marie Adams, John I. Risinger, Galen Hostetter, Ken Lin, Ronald L. Chandler

**Affiliations:** 1 Department of Obstetrics, Gynecology and Reproductive Biology, College of Human Medicine, Michigan State University, Grand Rapids, Michigan, United States of America; 2 Genomics Core Facility, Van Andel Research Institute, Grand Rapids, Michigan, United States of America; 3 Department of Women’s Health, Spectrum Health System, Grand Rapids, Michigan, United States of America; 4 Pathology and Biorepository Core, Van Andel Research Institute, Grand Rapids, Michigan, United States of America; 5 Department of Obstetrics & Gynecology and Women’s Health, Albert Einstein College of Medicine, Bronx, New York, New York, United States of America; 6 Department of Epigenetics, Van Andel Research Institute, Grand Rapids, Michigan, United States of America; University of Michigan Michigan Medicine, UNITED STATES

## Abstract

TP53 and ARID1A are frequently mutated across cancer but rarely in the same primary tumor. Endometrial cancer has the highest TP53-ARID1A mutual exclusivity rate. However, the functional relationship between TP53 and ARID1A mutations in the endometrium has not been elucidated. We used genetically engineered mice and *in vivo* genomic approaches to discern both unique and overlapping roles of TP53 and ARID1A in the endometrium. TP53 loss with oncogenic PIK3CA^H1047R^ in the endometrial epithelium results in features of endometrial hyperplasia, adenocarcinoma, and intraepithelial carcinoma. Mutant endometrial epithelial cells were transcriptome profiled and compared to control cells and ARID1A/PIK3CA mutant endometrium. In the context of either TP53 or ARID1A loss, PIK3CA mutant endometrium exhibited inflammatory pathway activation, but other gene expression programs differed based on TP53 or ARID1A status, such as epithelial-to-mesenchymal transition. Gene expression patterns observed in the genetic mouse models are reflective of human tumors with each respective genetic alteration. Consistent with TP53-ARID1A mutual exclusivity, the p53 pathway is activated following ARID1A loss in the endometrial epithelium, where ARID1A normally directly represses p53 pathway genes *in vivo*, including the stress-inducible transcription factor, ATF3. However, co-existing TP53-ARID1A mutations led to invasive adenocarcinoma associated with mutant ARID1A-driven ATF3 induction, reduced apoptosis, TP63+ squamous differentiation and invasion. These data suggest TP53 and ARID1A mutations drive shared and distinct tumorigenic programs in the endometrium and promote invasive endometrial cancer when existing simultaneously. Hence, TP53 and ARID1A mutations may co-occur in a subset of aggressive or metastatic endometrial cancers, with ARID1A loss promoting squamous differentiation and the acquisition of invasive properties.

## Author summary

Endometrial cancer is the most commonly diagnosed gynecologic malignancy in the United States, with annual incidence continuing to rise. Although the majority of endometrial cancer patients have an excellent overall prognosis if the disease is confined to the endometrium, myometrial invasion and metastasis to other sites correlate with poor survival. Here, we used genetically engineered mice, *in vivo* genomics, and public cancer patient data to understand the relationship between TP53 and ARID1A, two of the most commonly mutated genes in endometrial cancer, in the context of mutant PIK3CA. Mutations in TP53 and ARID1A change different aspects of endometrial cell health but also share some similarities. ARID1A mutations specifically promote cancer cells to invade nearby tissue, a hallmark of metastasis, associated with squamous differentiation. Mice with co-existing TP53 and ARID1A mutations developed more invasive disease. Our studies suggest that co-existing TP53 and ARID1A tumor mutations may promote invasion and metastasis.

## Introduction

*TP53* (p53) and *ARID1A* are among the most frequently mutated tumor suppressor genes across cancer [1]. The historic tumor suppressor roles of TP53 have been well characterized in numerous reports since its discovery, when it was found interacting with the transforming agent SV40 large T antigen [2,3]. Meanwhile, the functions of ARID1A in cellular homeostasis and carcinogenesis have only recently been described since exome studies revealed widespread mutation prevalence in disease [4,5]. Both proteins serve roles in transcriptional regulation—ARID1A is a SWI/SNF chromatin remodeling complex subunit [5], while TP53 is a transcription factor [6]. Evidence supports TP53 and ARID1A also have other important nuclear functions including DNA repair and cell cycle regulation [7–10].

TP53 and ARID1A mutations are frequent among gynecologic cancers [11–16]. Both genes are commonly mutated in ovarian and uterine cancers, and there are mutation-defining subtypes within each cancer [13,17–21]. However, an early mechanistic study showed biochemical and functional evidence linking ARID1A and TP53 regulation and mutant ARID1A-TP53 mutual exclusivity in a cohort of 77 ovarian clear cell and uterine endometrioid carcinomas, where all ARID1A mutant tumors were TP53 wild-type, and vice versa [22]. Since then, numerous reports have observed ARID1A and TP53 alterations co-occur less frequently than expected by chance in other human cancer types, including gastric, breast, and esophageal [23–26]. Among gynecologic cancers, loss of ARID1A expression by immunohistochemical staining was significantly associated with wild-type TP53 expression in high-grade endometrial tumors [27]. Within the endometrioid subtype of endometrial cancer, one study observed that tumors marked by high TP53 expression, indicative of TP53 mutation, almost never displayed low/absent ARID1A expression [28].

In this study, we show that endometrial cancer displays the highest mutual exclusivity rate for TP53 and ARID1A mutations, irrespective of histological subtype, across over 10,000 human tumors profiled by The Cancer Genome Atlas (TCGA). We develop a genetically engineered mouse model with co-existent TP53 loss of function and oncogenic PIK3CA^H1047R^ activation specifically in the endometrial epithelium. To discern both overlapping and distinct molecular features associated with TP53 or ARID1A mutation, endometrial epithelial cells were isolated from this model, profiled by RNA-seq, and compared to control cells and ARID1A/PIK3CA mutant cells. These mouse model data were compared with human tumor data to determine cross-species gene expression signatures associated with TP53 and ARID1A mutation status. We show that ARID1A mutant tumors display p53 pathway activation in endometrial cancer and across cancer, and ARID1A directly regulates TP53 target genes *in vivo*. Finally, we developed mice simultaneously harboring endometrial mutations in TP53, ARID1A, and PIK3CA, which develop aggressive and highly invasive cancer. We further show that ARID1A directly represses promoter chromatin at target gene *Atf3*, and ATF3 induction in ARID1A mutant cells is associated with invasive squamous differentiation independent of TP53 mutation status. These studies reveal that co-existing TP53 and ARID1A mutations promote invasive endometrial cancer.

## Results

### TP53 and ARID1A mutations rarely co-occur in endometrial cancer

**Fig 1:**
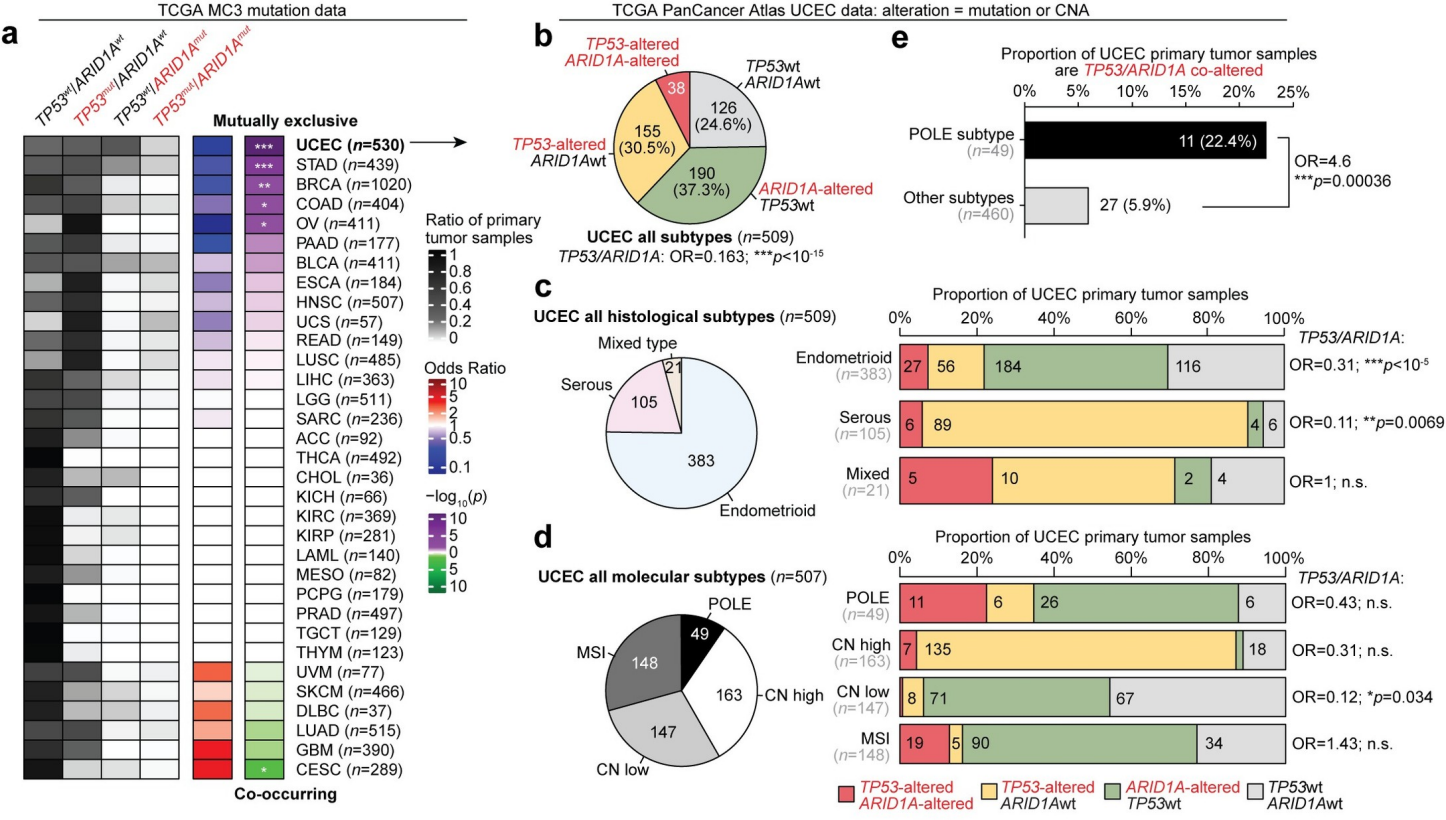
TP53 and ARID1A mutations rarely co-occur in endometrial cancer. **a**, Pan-cancer analysis of TP53 and ARID1A mutation rates across 33 TCGA tumor types, only considerate of somatic single nucleotide variants. For heatmap, darker color indicates a greater proportion of sequenced tumor samples. Odds ratio (OR) and statistics for each tumor type accompany two-tailed Fisher’s exact tests performed on TP53 and ARID1A mutation contingency tables. Asterisks indicate significant associations between TP53 and ARID1A mutations, either co-occurring or mutually exclusive: * *p* < 0.05; ** *p* < 0.01; *** *p* < 0.001. **b**, Details within the Uterine Corpus Endometrial Carcinoma (UCEC) cohort (*n* = 509), further inclusive of copy number alteration (CNA) events. TP53 and ARID1A mutation classes (left) and distribution of histological subtypes (right) across the UCEC cohort. **c**, Left, distribution of UCEC histological subtypes: endometrioid, serous, and mixed. Right, TP53 and ARID1A alteration rates and association of co-occurrence for primary tumors within each histological subtype. **d**, As in **c** but for UCEC molecular subtypes: POLE mutant, copy-number alteration high (CN high), CN low, and microsatellite instable (MSI). **e**, Association between POLE mutant molecular subtype tumors and TP53/ARID1A co-alterations. Statistic is two-tailed Fisher’s exact test.

We quantified the co-mutation rates for TP53 and ARID1A across 10,144 primary tumor samples from 33 cancer types profiled by TCGA, through the standardized MC3 mutation data set [29] (Fig 1A). Five of 33 cancer types (uterine corpus endometrial carcinoma, UCEC; stomach adenocarcinoma, STAD; breast invasive carcinoma, BRCA; colon adenocarcinoma, COAD; ovarian serous cystadenocarcinoma, OV) display significant mutual exclusivity (two-tailed Fisher’s exact test, *p* < 0.05), indicating these mutations co-occur less frequently than expected by chance. One tumor type, cervical squamous cell carcinoma and endocervical adenocarcinoma (CESC), indicated the opposite, that mutations in these two genes were occurring more frequently than expected. Uterine corpus endometrial carcinoma (UCEC) [18] displayed the highest rate of TP53-ARID1A mutual exclusivity out of all profiled cancer types (two-tailed Fisher’s exact test, OR = 0.155, *p* < 10^−20^).

We further investigated TP53 and ARID1A genetic alteration status in UCEC via the TCGA Pan-Cancer Atlas data set [30], which includes 509 primary tumor samples with both mutation and copy number alteration (CNA) data (Fig 1B). Tumors were considered “altered” for a gene if they displayed either a mutation or CNA event at each locus. In the combined data set of all disease subtypes, TP53 and ARID1A mutations were co-altered in 7.5% of tumor samples, while the independent alteration frequencies for each gene were above 30% (Fig 1B). The UCEC data set is comprised of three distinct histological disease subtypes—endometrioid, serous, and mixed type morphology—which have different incidence rates and are strongly associated with TP53 and ARID1A status [15,18,31,32]. The original TCGA-UCEC report also classified four molecular subtypes based on integrative multi-omic analyses: POLE ultra-mutated, copy-number alteration high (CN high), copy-number alteration low (CN low), and microsatellite instable (MSI) [33]. Therefore, we sought to determine if TP53-ARID1A genetic mutual exclusivity could be attributed to sampling error by investigating alterations within each histological and molecular subtype independently. In the two predominant endometrial cancer histological subtypes, endometrioid and serous, TP53 and ARID1A alterations co-occurred less frequently than expected by chance in primary tumors (two-tailed Fisher’s exact test, Fig 1C). Across the molecular subtypes, only CN low tumors displayed significant ARID1A-TP53 mutual exclusivity (OR = 0.12, Fig 1D). The other molecular subtypes are characterized by heightened genomic instability, which are more likely to harbor passenger mutations in those tumor subtypes. Supporting this, POLE ultra-mutated primary tumors are more associated with TP53/ARID1A co-alterations compared to other subtypes (OR = 4.6, two-tailed Fisher’s exact test, Fig 1E). Overall, these analyses suggest that mutually exclusive TP53 and ARID1A alterations are observed in primary uterine endometrial tumors of both endometrioid and serous subtypes and are notable in CN low tumors.

### TP53 loss in the presence of PIK3CA^H1047R^ drives hyperplasia and endometrial intraepithelial carcinoma

We previously reported that ARID1A loss paired with PI3K activation through constitutive expression of oncogenic PIK3CA^H1047R^ drives endometrial hyperplasia and myometrial invasion in mice [34,35]. In addition, we also showed that ARID1A and PIK3CA mutations frequently co-occur in UCEC tumor samples [34]. Upon further examination of TCGA-UCEC data, we found that roughly half of TP53 mutant tumors also harbor PIK3CA mutations (S1A Fig). Since TP53 and PIK3CA mutations are frequently observed together, we tested whether TP53 mutations could also promote endometrial tumorigenesis in the presence of PIK3CA^H1047R^ in mice.

**Fig 2:**
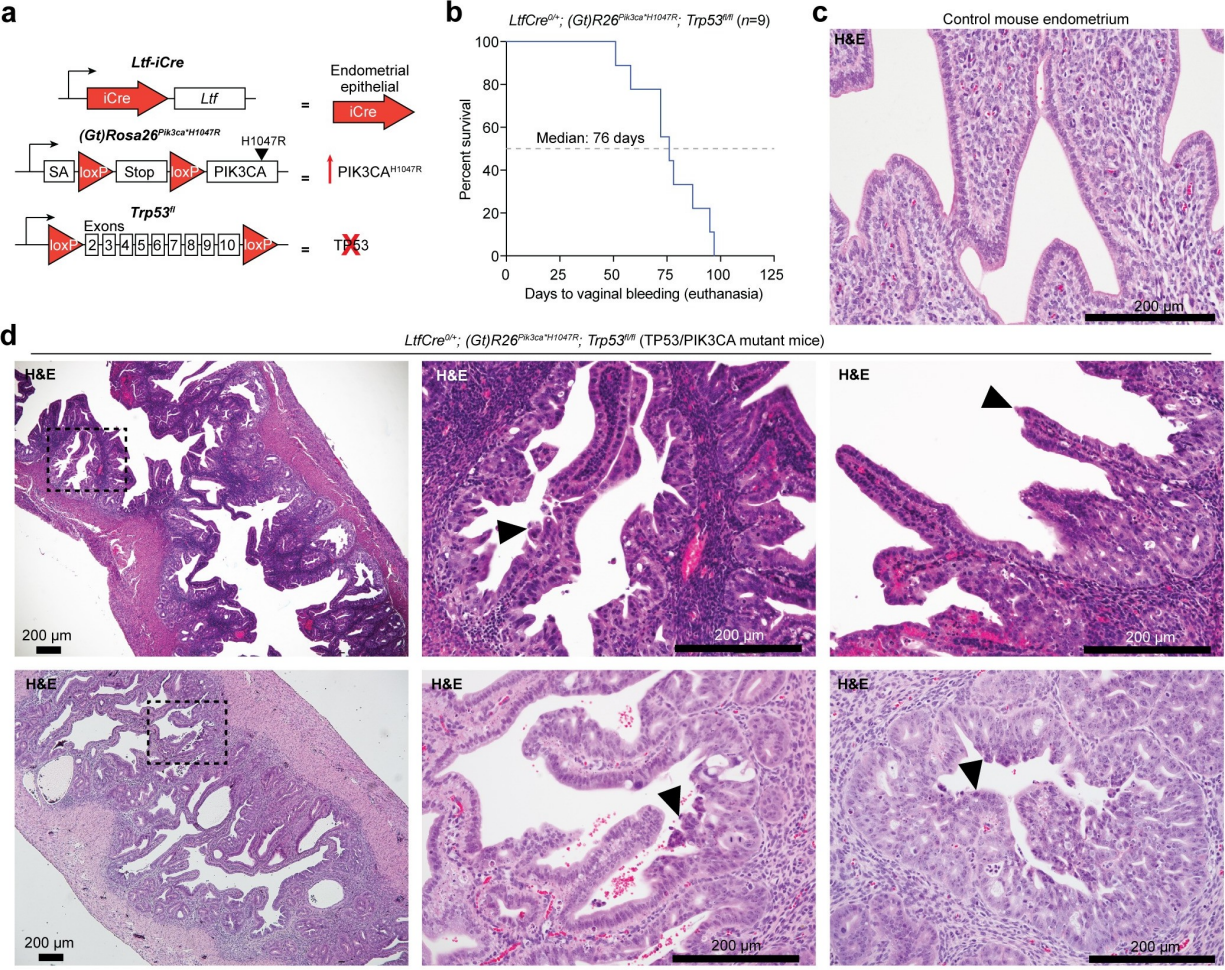
TP53 loss with oncogenic PIK3CA activation results in endometrial intraepithelial carcinoma. **a**, Diagram of mouse alleles used in this study. **b**, Survival data of *LtfCre*^*0/+*^*; (Gt)R26*^Pik3ca*H1047R^*; Trp53*^*fl/fl*^ (TP53/PIK3CA mutant) mice. Survival is measured as days to vaginal bleeding, requiring euthanasia. **c**, Representative H&E histology of control mouse endometrium from CRE-negative littermates. **d**, Representative H&E histology of endometrial intraepithelial carcinomas and hyperplastic epithelia in TP53/PIK3CA mutant uterus. Arrowheads denote dyplastic endometrial epithelia.

TCGA-UCEC data indicates that roughly 19% of *TP53* mutations in uterine serous carcinoma putatively result in direct TP53 protein truncation through frameshift or splice site alteration, and missense vs. truncating *TP53* mutations are not associated with differences in survival or tumor grading (S1B–S1E Fig). Therefore, we modeled the effects of TP53 loss in combination with *PIK3CA* mutation in the endometrium by crossing the *Trp53*^*fl*^ allele [36] with *(Gt)R26*^Pik3ca*H1047R^ [37] in *LtfCre*^*+*^ mice (Fig 2A). The *LtfCre* allele results in tissue-specific Cre recombinase expression in the endometrial epithelium at onset of puberty [38]. Vaginal bleeding, indicating endometrial dysfunction, was observed with biallelic loss of TP53 in the presence of PIK3CA^H1047R^ at a median of 76 days (Fig 2B). Compared to control mice (Figs 2C and S2A), histological analysis of *LtfCre*^*0/+*^*; (Gt)R26*^Pik3ca*H1047R^*; Trp53*^*fl/fl*^ mice (henceforth referred to as TP53/PIK3CA mutant mice) revealed features of hyperplasia, adenocarcinoma, and endometrial intraepithelial carcinoma (EIC) within luminal and glandular areas (Figs 2D and S2B). Mutant endometrial epithelial cells expressed KRT8, a marker of endometrial epithelium, and phospho-S6, a marker of PI3K pathway activity (S2C Fig). EIC is typically considered a precursor lesion to uterine serous carcinoma, a subtype of endometrial cancer dominated by TP53 mutations (see Fig 1C) [39,40]. EIC is marked by high-grade cytology and often presents as non-invasive with hobnail and papillary morphologies [41–43], although nuclear atypia was infrequently observed in TP53/PIK3CA mutant mice. This non-invasive, EIC-like morphology contrasts with the ARID1A loss-driven invasive hyperplasia observed in *LtfCre*^*0/+*^*; (Gt)R26*^Pik3ca*H1047R^*; Arid1a*^*fl/fl*^ mice (henceforth referred to as ARID1A/PIK3CA mutant mice) [34]. Stromal or myometrial invasion is not observed in the uterus of TP53/PIK3CA mutant mice, while collective invasion is a critical pathological feature of ARID1A/PIK3CA mutant endometrial epithelia [34]. In the context of mutant PIK3CA, these results indicate that TP53 mutation in the endometrial epithelium promotes an endometrial phenotype that is distinct from ARID1A mutation, suggesting distinct tumor suppressive mechanisms.

### Endometrial phenotypes driven by TP53 or ARID1A loss display overlapping and distinct gene expression signatures

**Fig 3:**
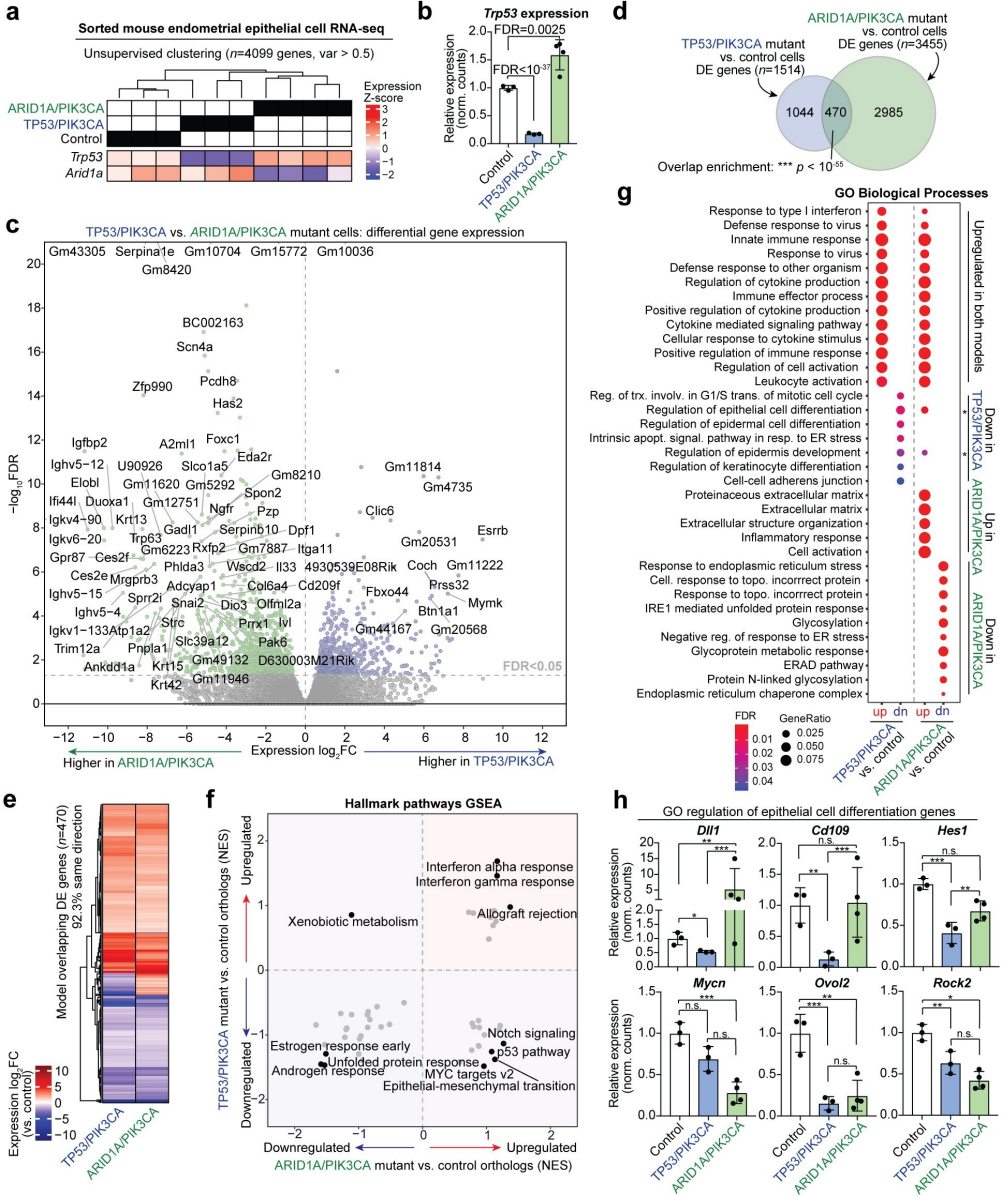
Endometrial epithelial TP53 and ARID1A loss results in overlapping and distinct gene expression programs. **a**, Unsupervised hierarchical clustering of gene-level RNA-seq data from sorted endometrial epithelial cells of TP53/PIK3CA mutant mice compared to ARID1A/PIK3CA mutant and control cells. Relative Z-score expression of targeted genes are displayed below clustering result. **b**, Relative linear *Trp53* expression in endometrial epithelial cell transcriptomes. **c**, Volcano plot depicting differential gene expression between TP53/PIK3CA mutant and ARID1A/PIK3CA mutant cells. **d**, Overlap of DE genes between TP53/PIK3CA mutants and ARID1A/PIK3CA mutant cells vs. controls. Statistic is hypergeometric enrichment. **e**, Heatmap of 470 shared dysregulated genes in endometrial epithelial cells from each genetic model. 92.3% of intersecting DE genes are affected in the same direction. **f**, Overview of Broad GSEA results for MSigDB Hallmark pathways in endometrial epithelial cells from each genetic model. Axes display gene set normalized enrichment score (NES) for each model compared to control cells. **g**, Enrichment for Gene Ontology (GO) Biological Process gene sets among genetic model DE genes separated by directionality. **h**, Examples of DE genes within the GO regulation of epithelial cell differentiation gene set: *DLL1*, *CD109*, *HES1*, *MYCN*, *OVOL2*, *ROCK2*. Statistic is FDR as reported by *DESeq2*: * FDR < 0.05; ** FDR < 0.01; *** FDR < 0.001.

In order to dissect tumorigenic mechanisms resulting from ARID1A or TP53 mutations in the context of mutant PIK3CA, we isolated endometrial epithelial cells from TP53/PIK3CA mutant mice at onset of vaginal bleeding (*n* = 3) using our previously developed sorting method [34] and performed RNA-seq. TP53/PIK3CA mutant endometrial epithelial cells were isolated with an average purity of 84.6% (S3 Fig). Transcriptome analysis was performed on these cells by comparing to RNA-seq data from our previously published control endometrial epithelial cells and hyperplastic *LtfCre*^*0/+*^*; (Gt)R26*^Pik3ca*H1047R^*; Arid1a*^*fl/fl*^ cells [34]. Samples grouped into genetically distinct clades by unsupervised hierarchical clustering (Fig 3A). TRP53 gene expression was significantly decreased in TP53/PIK3CA mutant cells compared to controls (FDR < 10^−37^) (Fig 3B). Interestingly, TRP53 expression was significantly upregulated in ARID1A/PIK3CA mutant cells (FDR = 0.0025) (Fig 3B). Directly comparing TP53/PIK3CA mutants to ARID1A/PIK3CA mutant cells resulted in 1799 significant differentially expressed (DE) genes at an FDR < 0.05 significance threshold (Fig 3C). Upon comparing each genetic model to control cells, 1514 genes were significantly affected in TP53/PIK3CA mutant cells, and 3455 genes were affected in ARID1A/PIK3CA mutant cells (Fig 3D). Of these gene sets, 470 DE genes overlapped between the genetic mouse models (hypergeometric enrichment, *p* < 10^−55^) (Fig 3D). Among overlapping DE genes, 92.3% of genes were affected in the same direction between both genetic models, which could be attributed to the PIK3CA^H1047R^ tumorigenic mechanisms shared in both models (Fig 3E).

Next, we asked what biological processes and pathways were affected in each genetic model. We performed Broad Gene Set Enrichment Analysis (GSEA) [44] for MSigDB Hallmark pathways [45] on human orthologs from each model compared to control cells. Comparing the GSEA normalized enrichment scores (NES) revealed that certain pathways were upregulated or downregulated in both genetic models, such as interferon responses (upregulated) and estrogen response (downregulated) (Fig 3F). However, many pathways were upregulated in ARID1A/PIK3CA mutant cells and downregulated in TP53/PIK3CA mutant cells (Fig 3F). These include Notch signaling, p53, epithelial-to-mesenchymal transition (EMT) and Myc targets. We previously reported that ARID1A transcriptionally represses mesenchymal fates through integrative genomic and cellular assays *in vivo* and *in vitro* [34].

Further investigation into the Hallmark EMT pathway revealed a cluster of 49 genes which are upregulated in both models as well as a cluster of 60 genes which are upregulated in ARID1A/PIK3CA mutant cells but mostly downregulated in TP53/PIK3CA mutants (S4A–S4D Fig). Among the affected genes, the EMT master regulator *SNAI2* is highly upregulated in ARID1A/PIK3CA mutants (4.54 log_2_FC vs. controls) but unaffected in TP53/PIK3CA mutant endometrial epithelial cells (S4E Fig). SNAI2/SLUG is a conserved transcription factor that directly represses epithelial gene transcription to regulate cellular processes like adhesion, polarity, migration, and invasion [46]. The observation that EMT-promoting factors are aberrantly upregulated in ARID1A mutant cells, but not in TP53 mutants, may explain the lack of collective invasion in TP53/PIK3CA mutant endometrial epithelia.

Enrichment for Gene Ontology (GO) Biological Process gene sets provided further insight into cellular processes affected in the genetic models (Fig 3G). As was observed in the GSEA analysis, interferon and immune pathways were enriched in genes commonly upregulated in both models, though various extracellular matrix pathways were uniquely enriched among upregulated genes in ARID1A/PIK3CA mutant cells (Fig 3G). Interestingly, no pathways were enriched among genes uniquely upregulated in the TP53/PIK3CA mutant model. Further, direct comparison of the two genetic models showed there were no significantly enriched gene sets among 603 human ortholog genes more highly expressed in TP53/PIK3CA mutant cells among the Hallmark, GO, and Oncogenic signature MSigDB Collections (S5 Fig). Among downregulated pathways, ARID1A/PIK3CA mutant cells downregulate ER stress response and glycosylation processes, while TP53/PIK3CA mutant cells downregulate G1/S mitotic transition transcriptional programs, apoptotic signaling pathways, and cellular differentiation pathways (Fig 3G). Notably, regulation of epithelial cell differentiation was enriched among genes downregulated in TP53/PIK3CA mutants but also genes upregulated in ARID1A/PIK3CA mutants. Further investigation into genes involved in this process showed that some appear to be oppositely affected following ARID1A or TP53 loss (*DLL1*), while others are uniquely affected by TP53 loss (*CD109*, *HES1*), uniquely affected by ARID1A loss (*MYCN*), or affected by loss of both TP53 and ARID1A (*OVOL2*, *ROCK2*) (Fig 3H). Altogether, these results highlight transcriptional programs with shared and unique regulation by ARID1A and TP53.

### Gene expression programs in mouse models reflect human tumor genetics

The molecular profiling data generated by TCGA serve as an excellent resource to support the human disease relevance of mouse model observations. We segregated UCEC bulk primary tumor samples by ARID1A and TP53 genetic mutation status and histological subtype, then performed Broad GSEA for the MSigDB collection of Hallmark pathways and GO Biological Process gene sets using RNA-seq data from each sample. The various UCEC comparisons included TP53mut/ARID1Awt vs. wt/wt, ARID1Amut/TP53wt vs. wt/wt, ARID1Amut/TP53wt vs. TP53mut/ARID1Awt, and endometrioid vs. serous. The same GSEA genetic comparison framework was also applied to the transcriptomic data generated from isolated mutant mouse cells compared to control cells and each other. GSEA results were then contrasted between the human and mouse comparisons, which were labeled as enriched by an absolute NES >1 threshold.

Hallmark pathway GSEA results for UCEC tumors corroborated certain observations in our genetic mouse models, such as upregulation of EMT, apoptosis, and p53 pathway in ARID1A mutant tumors, but downregulation of these pathways in TP53 mutant tumors (S6A Fig). As TP53 mutations are a hallmark of uterine serous carcinoma, while ARID1A mutations comprise roughly half of uterine endometrioid adenocarcinoma, we also observed that both Hallmark pathway and GO Biological Process GSEA results significantly correlated in a comparison between ARID1A mutant vs. TP53 mutant tumors and endometrioid vs. serous histological subtype (S6B and S6C Fig). We further confirmed the observed GSEA correlations were stronger than may be expected by chance due to sampling dependency (S6D Fig), suggesting that tumor gene expression features linked to TP53 or ARID1A genetic status are associated with histological subtype.

**Fig 4:**
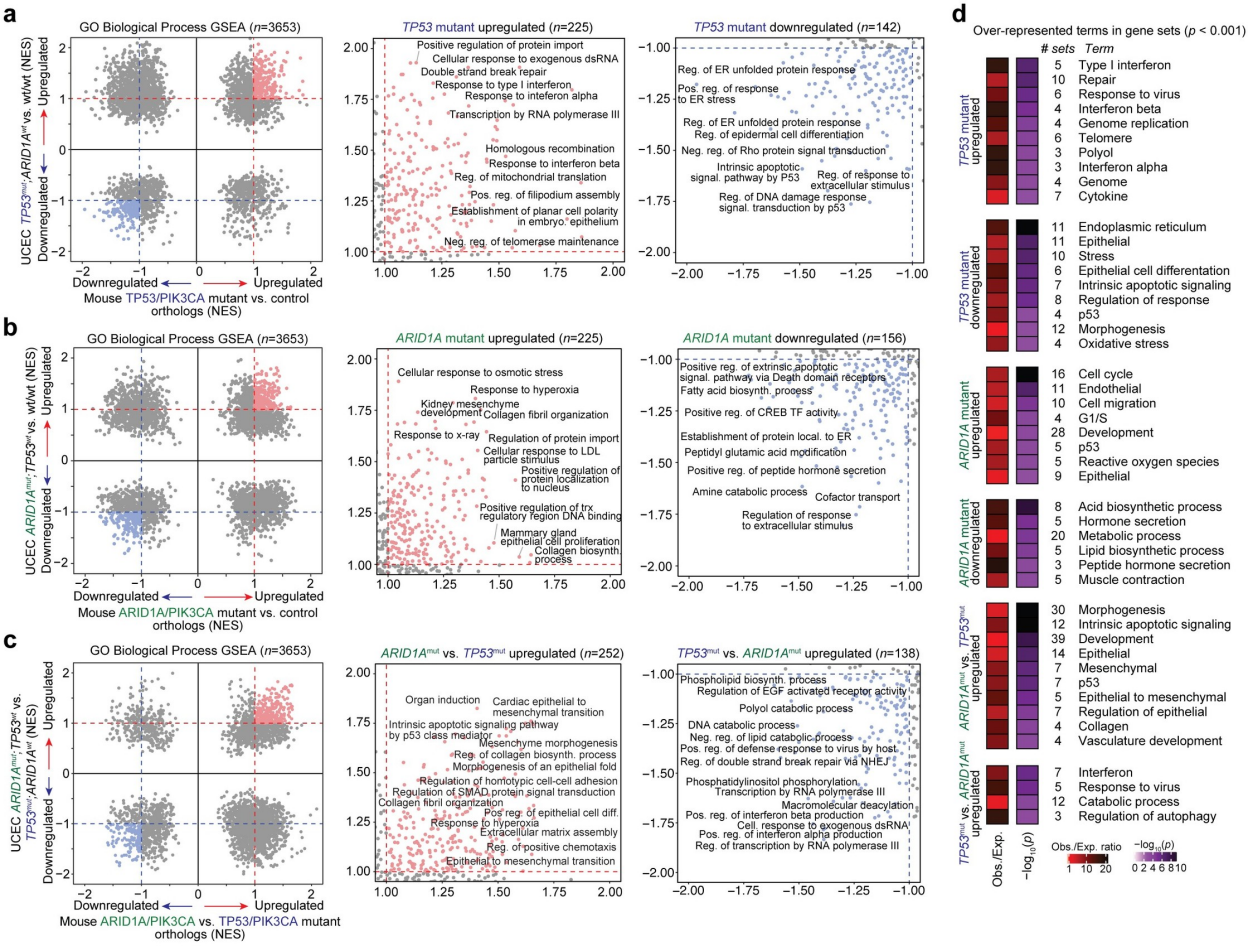
Pathway analysis of TP53 and ARID1A regulated expression programs in human disease and mouse models. **a**-**c,** Various Broad GSEA results for GO Biological Process gene sets (*n* = 3653) comparing TP53 and ARID1A mutant human UCEC tumors and genetically engineered mouse models: (**a**) TP53 mutant, ARID1A wild-type vs. wild-type/wild-type UCEC tumors compared to mouse endometrial epithelial cells from TP53/PIK3CA mutants vs. controls; (**b**) ARID1A mutant, TP53 wild-type vs. wild-type/wild-type UCEC tumors compared to mouse endometrial epithelial cells from ARID1A/PIK3CA mutants vs. controls; (**c**) *ARID1A* mutant, TP53 wild-type vs. TP53 mutant, ARID1A wild-type UCEC tumors compared to mouse endometrial epithelial cells from ARID1A/PIK3CA mutants vs. TP53/PIK3CA mutants. Presented are the overview of GSEA results (left) with zooms into shared upregulated (NES > 1, center) and shared downregulated (NES < -1, right) gene sets. Representative examples of highly enriched gene sets are labeled. **d**, Significantly over-represented terms in enriched gene sets (|NES| > 1) highlighted in **a**-**c**. Statistic is hypergeometric enrichment. See Materials and Methods for enrichment analysis framework.

From 3653 total GO Biological Process gene sets queried in all comparisons, 225 were mutually upregulated and 142 were mutually downregulated between TP53mut/ARID1Awt vs. wt/wt UCEC tumors and TP53/PIK3CA mutant vs. control mouse cells (Fig 4A). Upregulated TP53 mutant gene sets include response to type I interferon (NES 1.80 and 1.82, respectively) and double strand break repair (NES 1.93 and 1.11, respectively). Downregulated gene sets include intrinsic apoptotic signaling pathway by p53 (NES -1.59 and -1.49) and regulation of response to extracellular stimulus (NES -1.59 and -1.02). With regard to ARID1A mutant comparisons, 225 gene sets were mutually upregulated and 156 were mutually downregulated between ARID1Amut/TP53wt vs. wt/wt UCEC tumors and ARID1A/PIK3CA mutant vs. control mouse cells (Fig 4B). Upregulated ARID1A mutant gene sets include response to hyperoxia (NES 1.81 and 1.39, respectively) and collagen fibril organization (NES 1.75 and 1.42). Curiously, in both the ARID1A and TP53 human-mouse disease comparisons, mutually downregulated gene sets (compared to wild-types or controls) overlapped more than expected by chance, while upregulated gene sets did not (hypergeometric enrichment, S7 Fig). Direct comparison of ARID1A mutant vs. TP53 mutant human tumors and mouse models furthered that many related processes appear to be distinctly affected in TP53 vs. ARID1A mutants, such as extracellular matrix assembly and EMT (Fig 4C). Notably, gene sets that were expressed higher in ARID1A mutants compared to TP53 mutants strongly overlapped between UCEC tumors and mouse models (*p <* 10^−8^, hypergeometric enrichment), while downregulated gene sets did not display significant overlap (S7 Fig). At the gene level, 81 genes were significantly more highly expressed in TP53 mutant mice and UCEC tumors as compared to ARID1A mutants, including *ESRRB*, *MAL*, *WNT7A*, *RASAL1*, *USP51*, *PLCXD3*, and *AIF1L* (S8 Fig). In contrast, 149 genes were significantly more highly expressed in ARID1A mutants, including *COL17A1*, *KRT5*, *TP63*, *SNAI2*, *ZNF750*, *HAS3*, *ANKK1*, *WDR38*, *C6*, and *IL33* (S8 Fig).

In order to characterize the affected pathways in TP53 vs. ARID1A mutant disease in an unbiased manner, we identified over-represented terms among enriched GO Biological Process gene sets. Upregulated TP53 mutant gene sets were enriched for terms such as “type I interferon” and “response to virus”, and downregulated sets involved terms “endoplasmic reticulum” and “epithelial” (Fig 4D). Upregulated ARID1A mutant gene sets were enriched for terms “cell cycle”, “cell migration”, “oxidative stress”, and “p53” (Fig 4D). Human-human and mouse-mouse genetic comparisons also showed that p53 pathway-related processes are consistently upregulated in ARID1A mutant tumors and downregulated in TP53 mutant tumors (S9 Fig).

### ARID1A mutant tumors display p53 pathway activation

**Fig 5:**
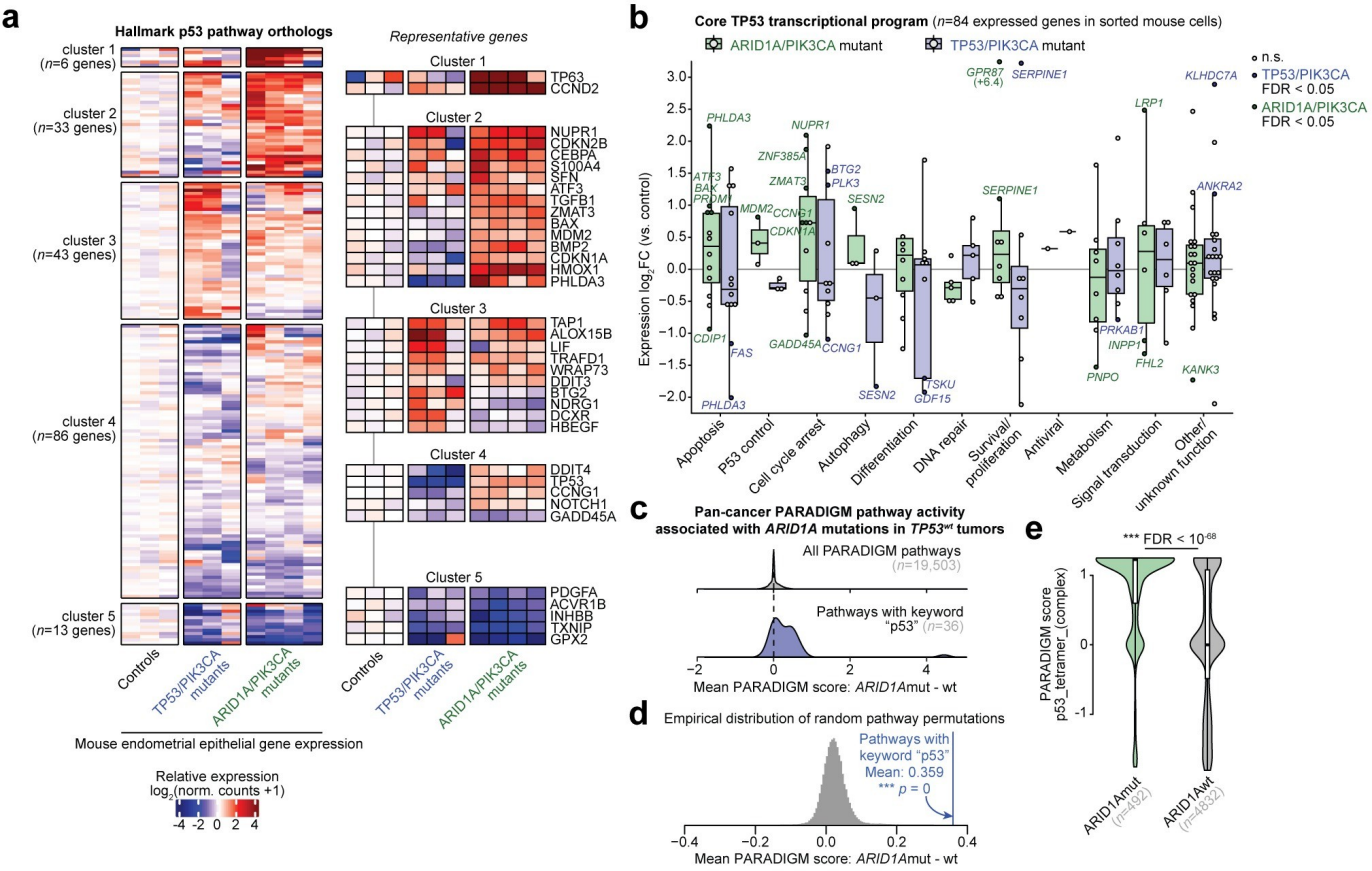
ARID1A mutation is associated with p53 pathway activation. **a**, *k*-means clustering and heatmap of genetic mouse model RNA-seq relative log_2_ gene expression data for MSigDB Hallmark p53 pathway genes (*n* = 181 expressed orthologs). Representative genes are highlighted on the right. **b**, Differential expression of core TP53 transcriptional program gene orthologs, segregated by function, in mouse endometrial epithelial cells from ARID1A/PIK3CA mutants (green) and TP53/PIK3CA mutants (blue) compared to controls. Significant DE genes (FDR < 0.05) in each model are labeled and respectively colored. **c**, Distribution of PARADIGM score differences between ARID1A mutant (*n* = 492) vs. wild-type (*n* = 4832) TCGA Pan-Cancer Atlas tumors, considerate of only *TP53* wild-type tumors. Top, all 19,503 measured pathways; bottom, the 36 pathways with keyword “p53”. **d**, Empirical distribution of mean differences between ARID1A mutant vs. wild-type PARADIGM scores, based on 50,000 samples of 36 random PARADIGM pathways. The blue line represents the mean score difference for the 36 pathways with keyword “p53” with associated permutation statistic. **e**, Example violin plot for the top p53 PARADIGM pathway significantly different between ARID1A mutant vs. wild-type tumors. Statistic is FDR-adjusted, two-tailed, unpaired Wilcoxon test.

p53 pathway gene signatures were upregulated in both ARID1A mutant UCEC tumors and the ARID1A/PIK3CA mutant genetic mouse model. This result suggests tumor cell dependencies on the p53 pathway itself could be a potential mechanism underpinning mutual exclusivity of TP53 and ARID1A mutations. Mouse model analysis of gene expression within the Hallmark p53 pathway showed that certain canonical members of the p53 pathway were upregulated in ARID1A/PIK3CA mutant mice, such as TP63, TP53, MDM2, DDIT3 (CHOP), and CDKN1A (Fig 5A). As TP53 and ARID1A both regulate transcription, we proceeded further with an unbiased investigation to determine which aspects of TP53 directed transcriptional regulation are co-regulated by ARID1A in the endometrium. We interrogated a recently reported gene set composed of 103 high-confidence TP53 target genes that are transcriptionally regulated by TP53 in multiple cell lines, known as the core TP53 transcriptional program, which were further categorized based on known functions [47]. These genes were enriched for expression alterations in diseased endometrial epithelia from both ARID1A/PIK3CA and TP53/PIK3CA mutant mouse models (hypergeometric enrichment, *p* = 0.019 and 0.0063, respectively) (S10 Fig). We analyzed expression alterations of orthologous genes in TP53/PIK3CA mutant and ARID1A/PIK3CA mutant mice compared to controls (Fig 5B). Examples of opposing regulation by ARID1A and TP53 emerged, such as the pro-apoptotic Akt repressor PHLDA3 [48] which is upregulated in ARID1A/PIK3CA mutant mice but downregulated in TP53/PIK3CA mutant mice (Fig 5B).

Next, we tested whether p53 pathway activation is a hallmark feature of ARID1A mutant tumors across human cancer. We leveraged PARADIGM [49] pathway activity data produced by a recent pan-cancer TCGA study [30], which infers protein and pathway regulatory activity from both gene expression and copy-number data across 9829 tumors. In analysis of all TP53 wild-type primary tumors across cancer, we computed the mean difference in PARADIGM scores between ARID1A mutant and ARID1A wild-type tumors (*n* = 492 and 4832, respectively) to determine pathway alterations associated with ARID1A mutation (Fig 5C). Overall, the 36 PARADIGM pathways with keyword “p53” were more highly activated in ARID1A mutant tumors than ARID1A wild-type tumors across cancer (Fig 5C–5E, permutation test, *p* = 0). This result was also recapitulated specifically in UCEC tumors (S11 Fig), corroborating the GSEA results. Altogether, these data implicate aberrant p53-mediated transcriptional regulation as a hallmark feature of ARID1A mutant tumors. However, the functional mechanism underlying this activation remains unclear.

### p53 pathway target genes are directly regulated by ARID1A

**Fig 6:**
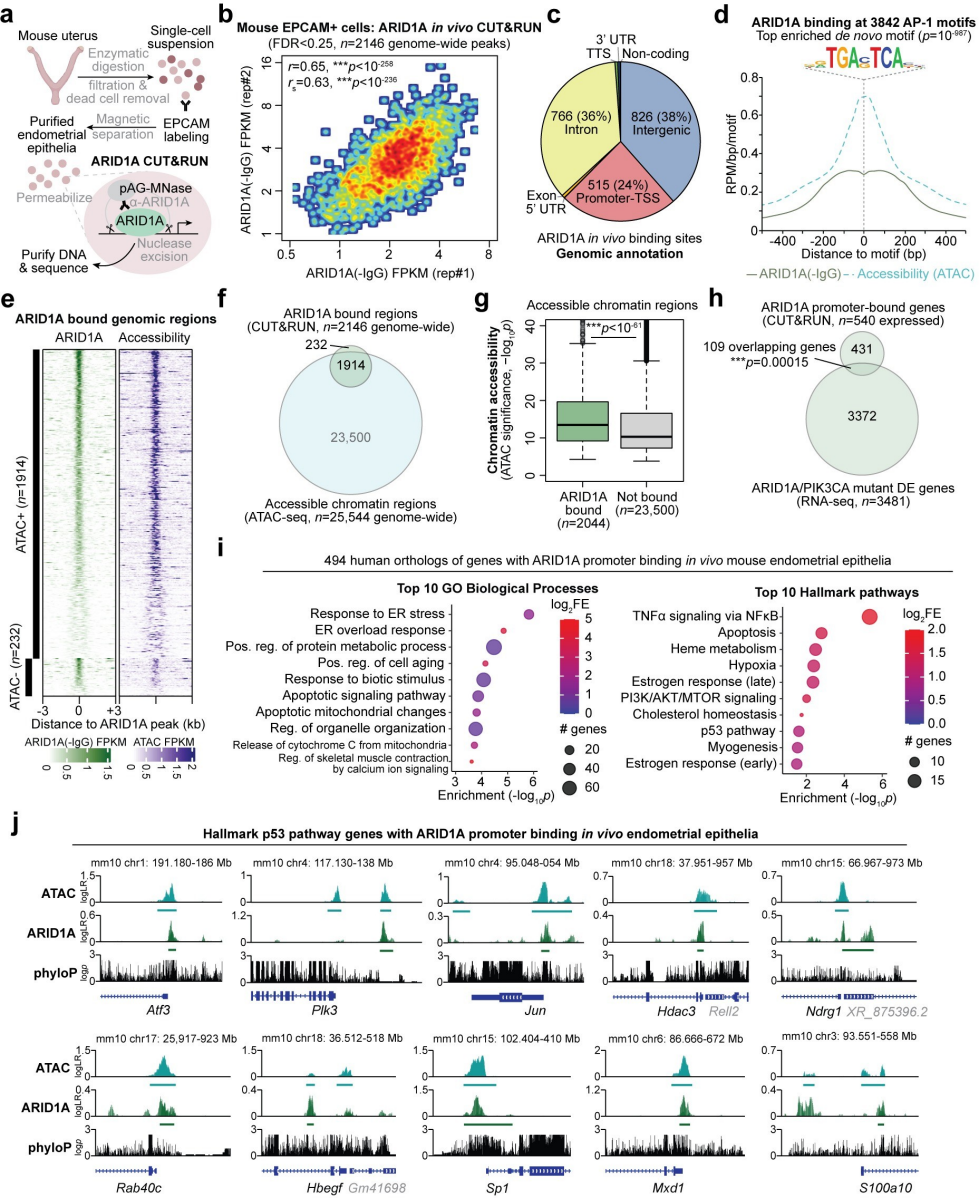
Analysis of ARID1A chromatin interactions in mouse endometrial epithelia *in vivo*. **a**, Diagram of experimental workflow for measuring ARID1A chromatin interactions *in vivo*. Endometrial epithelial cells are purified from mouse uterus by positive enrichment with labeling and magnetic beads. Purified cells were then subject to CUT&RUN for ARID1A or IgG negative control. **b**, Correlation of ARID1A binding signal (compared to IgG) *in vivo* across 2146 genomic regions with significant binding detected (FDR < 0.25) in two independent experiments. **c**, Genomic annotation of 2146 ARID1A bound genomic regions *in vivo*. **d**, ARID1A binding and accessibility profiles at 3842 bound AP-1/bZIP motifs, using the top *de novo* enriched motif among ARID1A genome-wide binding sites *in vivo*, TGA(G/C)TCA. **e**, Heatmap of ARID1A binding and chromatin accessibility signal across 2146 genomic regions with significant ARID1A binding detected, segregated by accessibility. **f**, Overlap of ARID1A bound regions and accessible chromatin regions. **g**, Chromatin accessibility quantified at accessible regions with significantly detected ARID1A binding vs. not. Statistic is two-tailed, unpaired Wilcoxon test. **h**, Significant overlap of genes with ARID1A promoter binding (within 3kb of TSS) and DE genes from ARID1A/PIK3CA mutant endometrial epithelia. **i**, Enrichment statistics for top 10 (left) GO Biological Process gene sets and (right) Hallmark pathways among 494 human gene orthologs with ARID1A promoter binding *in vivo* mouse endometrial epithelia. **j**, Examples of ARID1A chromatin interactions and accessibility (ATAC) at Hallmark p53 pathway genes *in vivo*. *y*-axis is log-likelihood ratio of signal compared to background. Bars underneath signal tracks represent significant (FDR < 0.25) and reproducible (*n* = 2) signal detection i.e. peaks. phyloP track represents sequence conservation across vertebrates.

We have previously shown that ARID1A normally represses key genes involved in endometrial pathologies through chromatin mechanisms [34,50]. We hypothesized that p53 pathway activation following ARID1A loss could result from the derepression of ARID1A target genes. To test this, we profiled ARID1A binding genome-wide in sorted mouse endometrial epithelial cells through *in vivo* CUT&RUN (Fig 6A) [51]. Our ARID1A *in vivo* CUT&RUN in mouse endometrial epithelial cells revealed significant ARID1A binding enriched over the IgG control at 2146 genome-wide sites (*MACS2*, FDR < 0.25, Fig 6B). These ARID1A bound genomic regions primarily comprised of intergenic (38%), intronic (36%), and promoter-TSS regions (24%, defined as within 3 kilobases of a gene transcription start site, TSS) (Fig 6C). Gene promoters and CpG islands represented the top enriched genomic features (S12A Fig). Sequence motif analysis of ARID1A bound genomic regions revealed strong enrichment for AP-1/bZIP family transcription factor binding sites (Figs 6D and S12B). We previously reported that ARID1A binds chromatin near gene promoters and AP-1 motifs *in vitro* 12Z human endometriotic epithelial cells [34].

As an orthogonal experimental control supporting the validity of these ARID1A binding data, we leveraged our previously reported chromatin accessibility data (ATAC-seq) in this same cell population [34]. We previously found that ARID1A binding is associated with accessible chromatin genome-wide *in vitro* 12Z human endometriotic epithelial cells [34]. In agreement, 89% of ARID1A bound regions *in vivo* overlapped with accessible chromatin (Fig 6E and 6F). We further observed that accessible chromatin regions bound by ARID1A were more highly accessible than those without detectable ARID1A binding (*p* < 10^−61^, two-tailed, unpaired Wilcoxon test, Fig 6G). These data support detection of ARID1A binding at active chromatin in mouse endometrial epithelial cells *in vivo*.

We next investigated genes regulated by ARID1A interactions at promoter chromatin in the endometrial epithelium. 587 annotated mouse genes had ARID1A binding detected within 3 kilobases of the primary TSS, indicating highly likely regulation of associated gene expression activity. In agreement with our expression data, we observed significant overlap between expressed genes with ARID1A promoter binding and DE genes in the ARID1A/PIK3CA mutant mouse model (Fig 6H). Similarly, we also observed significant overlap between the ARID1A/PIK3CA mutant DE genes and a more relaxed set of 3065 genes with ARID1A binding <50 kb from the gene TSS (S12C Fig). Functionally, 494 human orthologs of the ARID1A promoter bound genes were significantly enriched for gene sets including TNFα signaling via NFκB, apoptosis, hypoxia, and response to endoplasmic reticulum stress (Fig 6I). Overall similar pathways were enriched in the larger set of genes with ARID1A binding detected within 50 kb of the TSS (S12D Fig). Among the Hallmark p53 pathway, 11 genes had ARID1A binding detected within the ±3 kb promoter region in endometrial epithelia (* *p* = 0.025, hypergeometric enrichment) among those including *Atf3*, *Plk3*, *Jun*, *Ndrg1*, *Rab40c*, *Hbegf*, and *Mxd1* (Fig 6J). These data indicate that ARID1A regulates numerous target genes within the p53 pathway through direct chromatin interactions, and ARID1A loss may lead to aberrant gene expression by disrupting local chromatin regulation.

### Simultaneous TP53 and ARID1A loss promotes aggressive tumorigenesis

**Fig 7:**
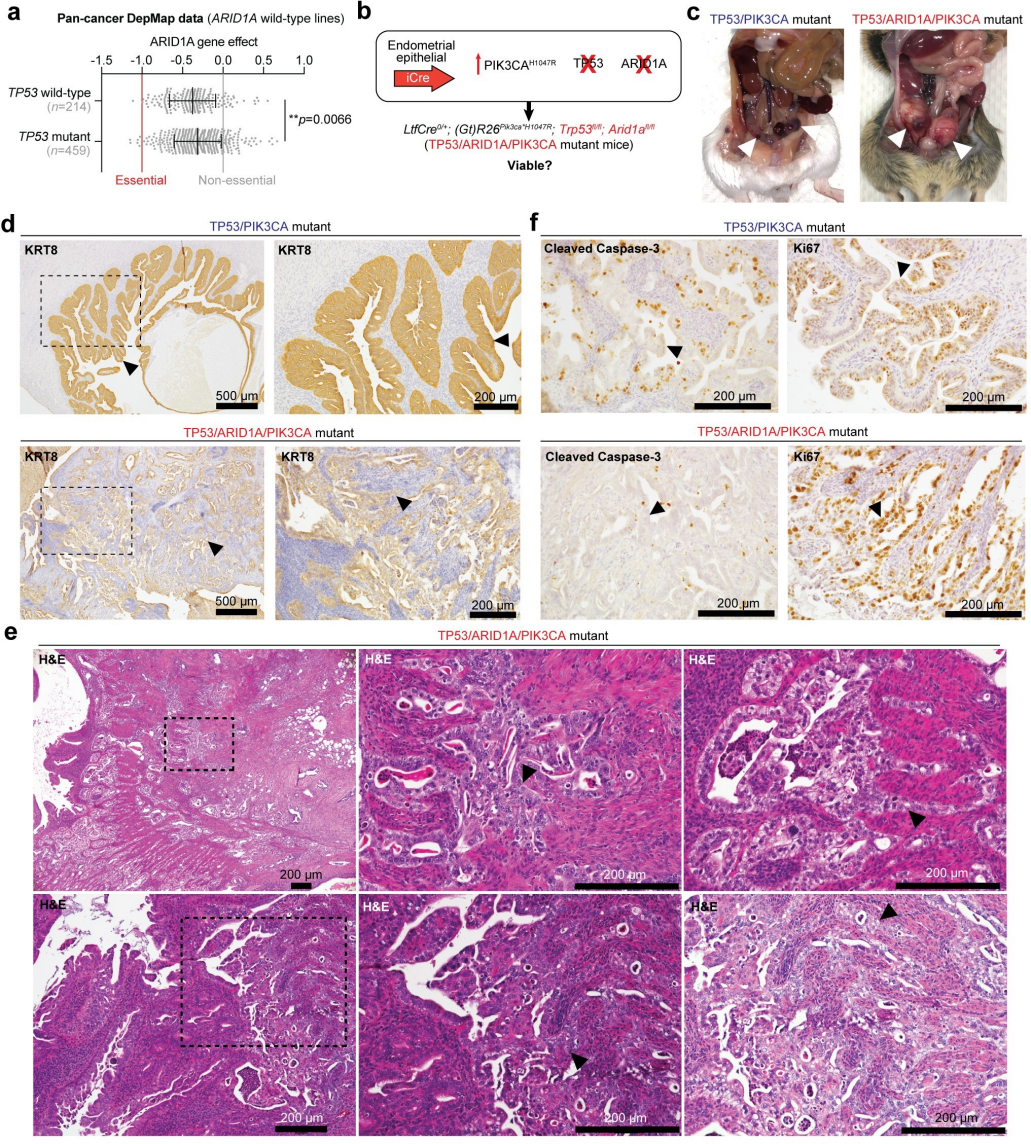
Co-existing TP53 and ARID1A mutations promote aggressive endometrial tumorigenesis. **a**, Cancer dependency map (DepMap) data for ARID1A wild-type cell lines, measuring ARID1A knockout viability effect on TP53 wild-type vs. mutant lines. Statistic is unpaired, two-tailed Wilcoxon test. **b**, Schematic of genetically engineered mice harboring endometrial epithelial specific PIK3CA^H1047R^, TP53 loss, and ARID1A loss. **c**, Example gross necropsy images in TP53/PIK3CA mutant (top) and *LtfCre*^*0/+*^*; (Gt)R26*^Pik3ca*H1047R^*; Trp53*^*fl/fl*^*; Arid1a*^*fl/fl*^ (TP53/ARID1A/PIK3CA mutant, bottom) mice. Arrowheads denote uterine abnormalities. **d**, Immunohistochemical staining of KRT8 staining in TP53/PIK3CA and TP53/ARID1A/PIK3CA mutant uterus. Arrowheads denote mutant endometrial epithelial cells. In TP53/ARID1A/PIK3CA mutant image. **e**, Representative H&E uterine histology images of TP53/ARID1A/PIK3CA mutant mice. Arrowheads depict mutant tumor cells with squamous differentiation. **f**, Uterine IHC staining for Cleaved Caspase-3 (cell death, left) and Ki67 (proliferation, right) in TP53/PIK3CA mutant (top) and TP53/ARID1A/PIK3CA mutant (bottom) mice. Arrowheads denote endometrial epithelial cells.

Despite the implication of p53 pathway activation as a hallmark of ARID1A mutant tumors, rare co-mutant patient tumors indeed exist, but the etiology and characteristics of which have not been elucidated. Intriguingly, the 38 UCEC primary tumors with alterations in both TP53 and ARID1A were associated with higher histologic tumor grading (S13A Fig), suggesting co-altered tumors may be more aggressive. We also found that TP53/ARID1A co-altered primary tumors were enriched for mixed morphology subtype (hypergeometric enrichment, *p* = 0.015) (S13B Fig). To determine if TP53 and ARID1A co-alterations were associated with metastasis, we examined the MSK-IMPACT Clinical Sequencing Cohort of 10,501 primary or metastatic tumor samples with targeted mutation data [52]. We observed that TP53/ARID1A co-mutation rates were slightly higher in metastatic tumors than in primary tumors (one-tailed Fisher’s exact test, OR = 1.20, *p* = 0.045) (S13C Fig). However, it is known that TP53 mutations are more prevalent in advanced stage and metastatic tumors compared to primaries [52]. When controlling for TP53 mutation status, we observed a similar, minor but insignificant association trend between *ARID1A* mutations and metastatic TP53 mutant tumors (OR = 1.10, *p* = 0.21) (S13D Fig). As in the TCGA-UCEC cohort, POLE mutant tumors in the MSK-IMPACT cohort were also enriched for TP53/ARID1A co-mutations (S13E Fig). To rule out the possibility of tumor heterogeneity contributing to TP53-ARID1A co-dependencies, Cancer Dependency Map (DepMap [53]) data suggested that TP53 mutant cancer cell lines were not more genetically dependent on *ARID1A* than TP53 wild-type lines (Fig 7A). On the contrary, ARID1A loss had a significantly lesser effect on cellular health in TP53 mutant lines (two-tailed, unpaired Wilcoxon test, *p* = 0.0066).

As ARID1A is an established metastasis gene in numerous tumor contexts [24,54–58], including endometrial cancer [59], we hypothesized that TP53 mutant primary tumors are not dependent on ARID1A function, and *ARID1A* mutations may promote metastasis in this genetic context. Therefore, we established and interrogated the tumorigenic potential of *LtfCre*^*0/+*^*; (Gt)R26*^Pik3ca*H1047R^*; Arid1a*^*fl/fl*^*; Trp53*^*fl/fl*^ mice (henceforth referred to as TP53/ARID1A/PIK3CA mutant mice), simultaneously harboring endometrial epithelial PIK3CA^H1047R^, ARID1A loss, and TP53 loss (Fig 7B). By gross observation and histopathology, TP53/ARID1A/PIK3CA mutant mice had visibly greater tumor burden as compared to TP53/PIK3CA mutant mice (Fig 7C and 7D). Vaginal bleeding was observed at a median of 98 days (S14A Fig). This latency period is significantly longer than TP53/PIK3CA mutant mice (S14A Fig), indicating that the humane survival endpoint of vaginal bleeding is not predictive of tumor burden in our mouse models, as we have previously observed [34]. Uterine histology of TP53/ARID1A/PIK3CA mutant mice showed invasive adenocarcinoma with areas of squamous differentiation (Fig 7D and 7E). Endometrial epithelial tumor origin was confirmed through staining for KRT8, phospho-S6, and loss of ARID1A (Figs 7D and S14B). Proliferation and caspase-mediated cell death indices of TP53/ARID1A/PIK3CA mutant endometrial epithelial cells were characterized through IHC staining for Ki67 and Cleaved Caspase-3, respectively. Compared to TP53/PIK3CA mutants, endometrial epithelia with additional ARID1A loss displayed markedly fewer caspase-3-positive foci while proliferation was not dramatically affected (Figs 7F and S15). These data suggest that co-occurring TP53-ARID1A mutations promote tumor cell progression towards invasive adenocarcinoma and metastatic disease.

### ARID1A loss results in ATF3 activation and squamous differentiation

We observed that endometrial epithelia harboring loss of both TP53 and ARID1A in addition to PIK3CA activation formed invasive tumors marked by high proliferation and minimal cell death. Compared to TP53/PIK3CA mice, which displayed frequent cell death marked by cleaved caspase-3-positive foci, the addition of ARID1A loss in TP53/ARID1A/PIK3CA mutant mice drastically reduced the number of apoptotic cells. This result suggests that ARID1A may normally promote cell death and stress-related programs in the absence of TP53, further supporting ARID1A regulation of apoptotic, inflammatory, and p53 pathway genes (see Fig 6).

Activation Transcription Factor 3 (ATF3) is a stress-inducible transcription factor and member of the Hallmark p53 pathway and apoptosis module of the core TP53 transcriptional program [47,60]. ATF3 has context-dependent roles in tumorigenesis, cell death, and cell cycle arrest or senescence [61–71]. ATF3 is known to both activate and repress transcriptional activity, and it co-regulates chromatin at p53 target genes [60,72,73]. ATF3 has also been shown to stabilize TP53 by competing for ubiquitination by MDM2 [74]. Further, ATF3 binds AP-1 consensus DNA motifs [75]—the top motif enriched among ARID1A binding sites (see S12B Fig)—indicating ATF3 may co-regulate ARID1A chromatin targets.

**Fig 8:**
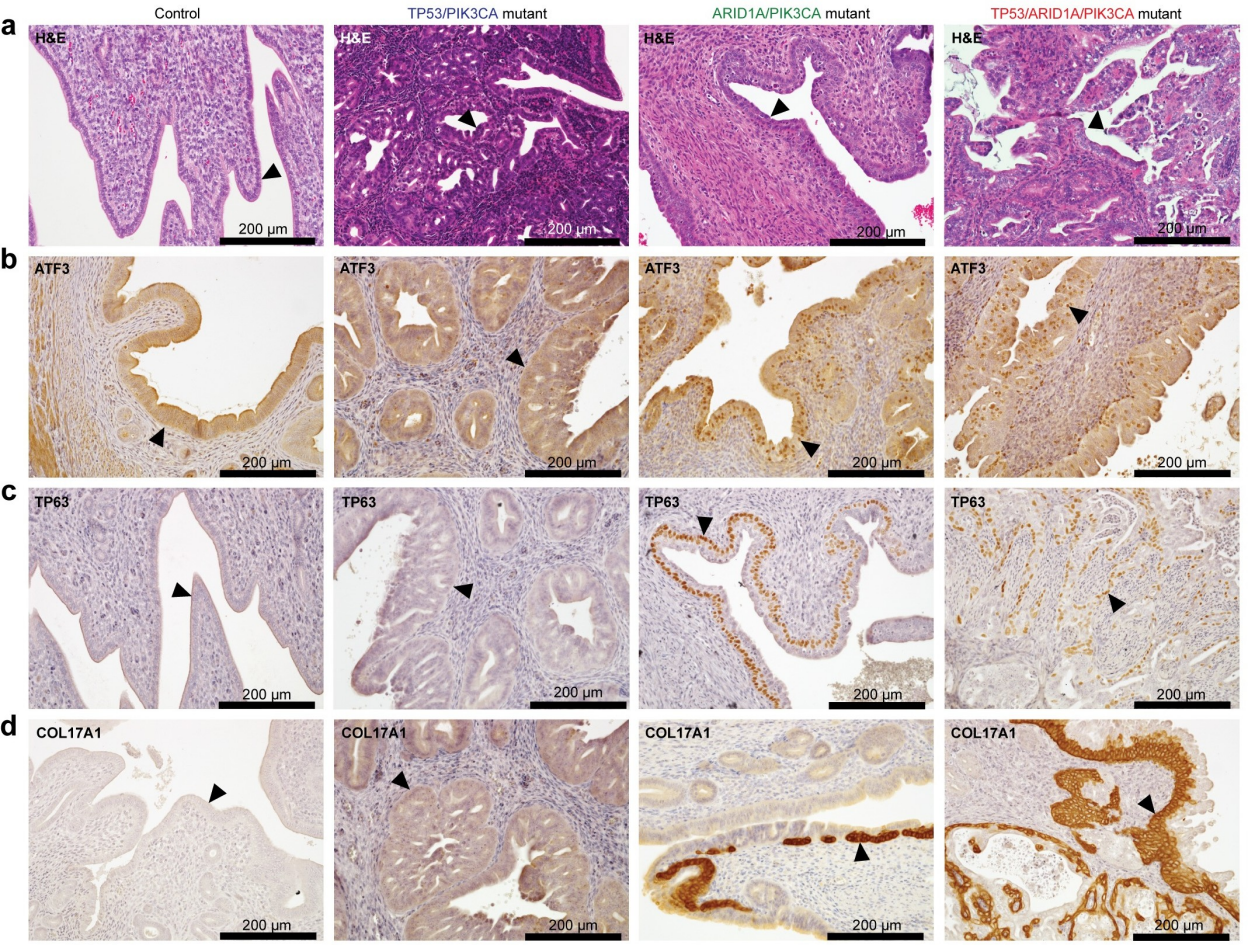
ARID1A loss relieves *Atf3* repression associated with squamous differentiation. **a**, Representative H&E histology of (from left to right) control mice, TP53/PIK3CA mutants, ARID1A/PIK3CA mutants, and TP53/ARID1A/PIK3CA mutants. Arrowheads depict endometrial epithelia. **b**-**d**, Immunohistochemical staining of ATF3 (**b**), TP63 (GeneTex) (**c**), and COL17A1 (**d**) of mouse sections as in **a**.

**Fig 9:**
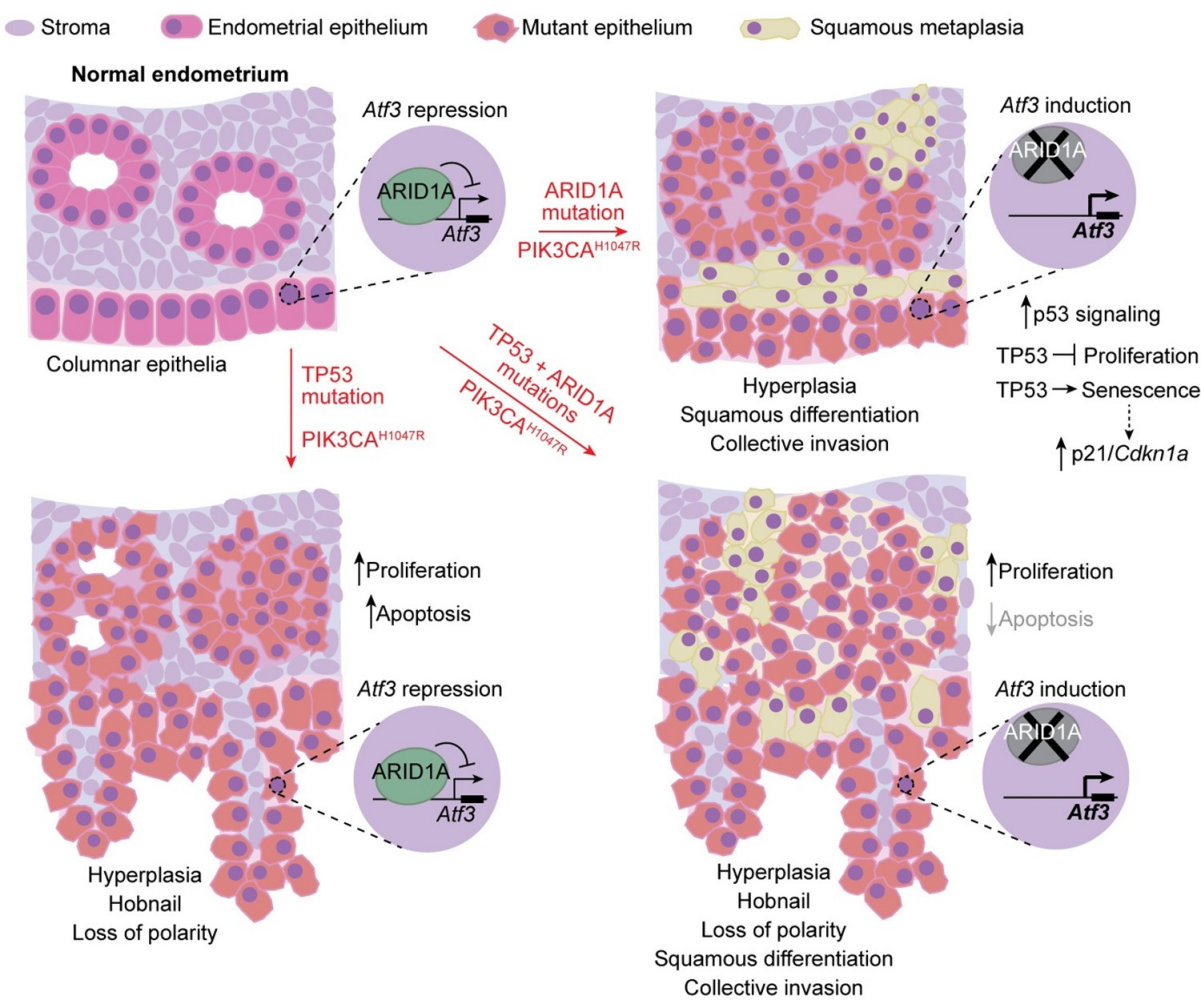
Model of independent and co-existing TP53 and ARID1A mutations in endometrial epithelia. Summary of endometrial disease features and hypothesized molecular mechanisms in genetically engineered mouse models from this study.

We observed that *Atf3* gene expression is directly repressed by ARID1A chromatin regulation in endometrial epithelia *in vivo* (Figs 6J and S16A). Uterine ATF3 staining revealed focal nuclear staining in a subset of endometrial epithelia in ARID1A/PIK3CA mutant and TP53/ARID1A/PIK3CA mutant mice, but not controls or TP53/PIK3CA mutants (Fig 8A and 8B). Among other roles, ATF3 is also dysregulated in some squamous tumor types [62,67,76,77], and ATF3 has been shown to promote epithelial squamous differentiation *in vivo* [68,78]. Areas of squamous-like cells were noted during histopathological analysis of TP53/ARID1A/PIK3CA mutant uteri, as well as induction of squamous marker TP63 in ARID1A/PIK3CA mutant mouse RNA-seq data (see Fig 5A), so we further examined markers of squamous differentiation. Nuclear TP63 staining was observed in both TP53/ARID1A/PIK3CA and ARID1A/PIK3CA mutant endometrial epithelia that neighbored basement membrane and those cells collectively invading (Figs 8C and S17A and S17B), confirming squamous differentiation, while TP63 expression was not observed in TP53/PIK3CA mutants or controls (Figs 8C and S17A and S17B). We also observed in our RNA-seq data that expression of *Trp63* (mouse TP63) correlated with pro-EMT transcription factor *Snai2* (S16B Fig), supporting an association between squamous differentiation and invasion phenotypes. Using a recently reported set of direct and functional TP63 target genes [79], we identified 30 genes through our data predicted as TP63 target genes associated with endometrial ARID1A mutation (*p* < 10^−4^, hypergeometric enrichment), including *COL17A1* (S16C–S16F Fig). In TP53/ARID1A/PIK3CA and ARID1A/PIK3CA mutant endometrial epithelia, COL17A1 expression patterns were similar to TP63 (Figs 8D and S17C). As further evidence that squamous phenotypes are associated with ATF3 expression, we observed expression of ATF3, TP63, and COL17A1 in normal vaginal pseudostratified squamous epithelia (S17D Fig). Collectively, these results suggest that ARID1A mediated repression of ATF3 promotes apoptosis in the absence of TP53, and derepression of ATF3 following ARID1A loss suppresses proapoptotic genes and promotes squamous differentiation (Fig 9). In ARID1A-deficient cells with wild-type TP53, enhanced TP53 signaling blocks proliferation and induces senescence.

## Discussion

Here, we develop new models of TP53 mutant endometrial dysfunction and provide genetic and molecular evidence in support of unique and overlapping roles for TP53 and ARID1A. We demonstrate that concurrent TP53 and oncogenic PIK3CA mutations in the endometrial epithelium led to the development of features of hyperplasia, adenocarcinoma, and endometrial intraepithelial carcinoma in the mouse, and additional ARID1A loss promotes invasive adenocarcinoma with squamous differentiation or metaplasia. Endometrial intraepithelial carcinoma is typically considered a precursor lesion to high-grade uterine serous carcinoma [43], an aggressive histologic presentation dominated by TP53 mutations. However, we cannot evaluate if the TP53/PIK3CA mutant mouse model develops uterine serous carcinoma due to the limitation of vaginal bleeding in early tumorigenesis. EIC lesions are non-invasive but frequently associate with extrauterine spread (e.g. to the peritoneum) [80]. Myometrial invasion in endometrioid tumors is associated with metastasis and poor outcomes. Acquisition of ARID1A mutation in EIC confined to the uterus may promote extrauterine dissemination. If TP53/PIK3CA and TP53/ARID1A/PIK3CA mutant mice were able to progress past the vaginal bleeding endpoint, it is possible that extrauterine dissemination could be observed.

Gene expression changes associated with TP53 and ARID1A mutations in the endometrium of humans and mice described here support mutation-specific programs that differentially affect cellular function, though commonalities are also found. Extensive pathway analysis in genetic mouse models and human tumors summarized distinct hallmarks of TP53 and ARID1A mutant tumors: TP53 mutations are associated with epithelial dedifferentiation and loss of intrinsic apoptosis and other p53-mediated cellular processes, while ARID1A mutations are associated with gene expression signatures related to EMT, cell cycle, cell migration, and the p53 pathway. PI3K pathway mutations are highly prevalent in endometrial cancer [33], and the aforementioned gene expression observations were noted in the context of oncogenic PIK3CA^H1047R^ in our genetic mouse models. In the endometrium, ARID1A directly regulates promoter chromatin at p53 target genes, which notably includes repression of *Atf3*. As reviewed, stress-induced ATF3 has numerous characterized roles in p53 signaling, cell death, and senescence, and our data support it as a marker of squamous epithelium. We hypothesize that ATF3 induction is associated with squamous differentiation and anti-apoptotic mechanisms in the endometrium.

Despite accounts of mutual exclusivity observed in primary tumors, our genetic mouse model experiments indicate co-existing TP53 and ARID1A mutations are tolerated *in vivo* and promote more aggressive cancer phenotypes than either mutation independently. Myometrial invasion is suggested to be driven at least partially through squamous differentiation following ARID1A loss, since the invasive front cells were TP63/COL17A1+. Given the invasiveness observed in TP53/ARID1A/PIK3CA co-mutant mice, we hypothesize that a human TP53 mutant endometrial cancer cell that acquires an ARID1A mutation will gain metastatic properties. This hypothesis may explain the rarity of TP53/ARID1A co-mutant samples in clinical cohorts like TCGA, which have mostly focused on sequencing primary tumors. The observation that TP53/ARID1A co-alterations are enriched among POLE mutant ultra-mutators could be interpreted possibly such that these are often non-functional passenger mutations. However, our data suggest that functional deleterious ARID1A mutation in a TP53 mutant primary may promote metastatic dissemination. In addition, it is worth noting that 3.3% of POLE wild-type tumors profiled by MSK-IMPACT are TP53/ARID1A co-mutant, which could be functional mutations. Outside of POLE ultra-mutators, our analysis of the TCGA molecular subtype framework suggests that TP53/ARID1A co-mutated primary UCEC tumors are most likely to present as microsatellite instable (MSI).

Intriguingly, 5 out of 57 primary tumors (8.8%) profiled in the TCGA uterine carcinosarcoma (UCS) data set harbored mutations in both *TP53* and *ARID1A* in the absence of hypermutator signatures [20]. A reported mouse model of uterine *Fbxw7* and *Pten* loss developed invasive endometrioid intraepithelial neoplasia followed by uterine carcinosarcomas at late stage confirmed as endometrial epithelial origin [81]. If our mouse models were able to age past the point of vaginal bleeding, it remains possible that a subset of TP53/ARID1A/PIK3CA mutant mice could progress toward uterine carcinosarcoma phenotype.

Currently, one large-scale sequencing study has focused specifically on endometrial cancer metastasis, and frequent, subclonal *ARID1A* mutations were observed in metastatic lesions [59]. In that study by Gibson et al., *ARID1A* mutations were more associated with late-occurring metastatic endometrial cancer clones as compared to other frequently mutated driver genes like *TP53* associated with early tumorigenesis. These results further support a role for ARID1A mutations as metastatic drivers in endometrial cancer. Another study examined cases of synchronous endometrial and epithelial ovarian cancers and found 4 out of 7 TP53 mutant tumors also harbored ARID1A mutations [82]. Additional clinical sequencing of matched primary and metastases from endometrial cancer patients would provide additional support for these findings.

Numerous reports have now shown in various tissue contexts that deleterious ARID1A alterations promote metastatic molecular and cellular signatures, such as EMT induction [34,83–86]. We also previously reported that ARID1A directly represses mesenchymal gene expression, and ARID1A loss promotes enhanced migration and invasion in the 12Z human endometriotic epithelial cell model [34]. However, *TP53* has also long been considered a metastasis gene in addition to displaying general tumor suppressor properties. Early studies discovered that de-differentiation and high-grade status of some tumor types were associated with TP53 loss and highly malignant behavior [87]. TP53 has been demonstrated to promote epithelialization through cell-cell adhesion and maintenance of the extracellular matrix, and it also regulates cell migration and invasion, stemness, EMT, and anoikis phenotypes [88]. Classically, uterine endometrioid adenocarcinomas, which are most associated with ARID1A mutations, are often indolent, low-grade tumors not generally associated with metastatic risk [31]. On the other hand, uterine serous carcinomas associated with TP53 mutations are generally higher grade and more aggressive [31]. Numerous genetically engineered mouse model studies have demonstrated that TP53 functions as a tumor suppressor in the endometrium [89–92]. Gene expression pathway analysis in TP53 mutant lesions here supports endometrial TP53 loss as leading to epithelial de-differentiation. This phenotype is distinct from ARID1A deficient lesions, which also lose epithelial features like cell adhesion and cell-cell junctions but acquire invasive, mesenchymal characteristics akin to canonical EMT or transdifferentiation [34], in addition to a subset of cells undergoing squamous metaplasia in our ARID1A mutant mouse model. In TP53 mutant serous primary tumor cells, it is hypothesized that further ARID1A mutation may trigger metastatic progression. In ARID1A mutant cells with intact TP53 signaling, we suspect that TP53 suppresses proliferation and induces senescence, given the phenotypes observed and upregulation of TP53 target genes, such as p21 (CDKN1A) and CHOP (DDIT3). In addition, senescence-associated secretory phenotypes (SASP) are associated with metastatic invasion [93]. Further experiments will be required to fully understand the phenotypic effects of ARID1A mutations in TP53 mutant cells, and vice versa.

Lack of invading features in TP53/PIK3CA mutant endometrial epithelia could be attributed to the use of a *Trp53*^*fl*^ allele, which may not completely model non-deleterious, gain-of-function TP53 mutations. In mice, *Trp53* missense alleles corresponding to known gain-of-function TP53 clinical mutations are associated with enhanced metastasis [94]. Using a gain-of-function mutation model may elicit different phenotypes in the endometrial epithelium than those observed with loss-of-function alleles, and these features could be cell type specific. In support of a nullizygous approach in the endometrium, *Trp53* null mutations in the mouse endometrium have been previously demonstrated to promote tumorigenesis [90,92]. In TCGA-UCEC samples, we show there is no significant difference in tumor grading or overall survival between TP53 missense vs. truncating mutations, further supporting the use of either strategy to study TP53 mutant tumors. Further, the type of TP53 mutation in endometrial cancer is not associated with genomic or histologic subtype [95]. Although a clinical sign of endometrial cancer in women, vaginal bleeding is a humane survival endpoint in mice, requiring euthanasia at early stages of the disease. In the absence of vaginal bleeding, it remains possible that endometrial invasion would be detected in TP53/PIK3CA mutant endometrium at later stages of the disease.

Mechanistically, ARID1A directly represses *Atf3* promoter chromatin in the endometrial epithelium, and ARID1A loss induces ATF3 expression associated with TP63+ squamous differentiation independent of TP53 mutation status. Similar to our findings, ARID1A downregulation in testicular germ cell tumors also leads to *ATF3* upregulation [96]. We also showed ATF3 expression in the vaginal squamous epithelium is strongest near basal epithelial cells marked by TP63 and COL17A1, indicating that ATF3 may be linked to squamous differentiation or metaplasia observed in ARID1A-deficient endometrial epithelia. Interestingly, previous reports have shown that ATF3 functions are context-dependent, and ATF3 may promote apoptosis or suppresses proapoptotic genes depending on whether it is activated in healthy versus tumor cells [71,72].

TP63 is a classical marker of squamous differentiation in cancer [97,98], and our studies suggest ATF3 induction is associated with TP63 expression in invading endometrial epithelial cells following ARID1A loss. Previous studies have identified roles for ATF3 in squamous tumors [62,67,76,77], and ATF3 promotes epithelial squamous differentiation *in vivo* [68,78]. Squamous differentiation is observed in 25% of human endometrial cancer and was recently associated with disease recurrence, and it has been observed in mouse models [99–101]. ARID1A and SWI/SNF mutations have been shown to promote carcinogenesis of various squamous tumor types [102,103]. We observed that ARID1A mutant UCEC tumors have higher TP63 expression than TP53 mutant tumors. A recent mouse model of ARID1A deletion paired with oncogenic KRAS^G12D^ developed invasive vaginal squamous cell carcinoma [104]. TP63+ squamous differentiation in invasive endometrial cancer was also recently reported in a subset of *Fbxw7/Pten* knockout mice [81]. Another mouse model with uterine-specific β-catenin deletion showed increased TP63 expression and squamous differentiation in endometrial epithelia [105]. TP63 marks basal endometrial epithelial cells during fetal life [106], and TP63+ basal cells are reactivated in GATA2 knockout and SOX17 knockout mouse uteri [107,108]. Transcriptional regulation by TP63 has been previously linked to cellular migration and invasion [109]. A subset of direct TP63 target genes are activated following ARID1A mutation in both genetic mouse models and human tumors, including COL17A1. Given the reported links between ATF3 and TP53 post-translational regulation, stability, and target gene regulation, it remains possible that ATF3 regulates TP63. Additional work will be required to determine the functional relationship between ATF3 and TP63 in endometrial squamous differentiation.

Regulation of apoptotic processes, normally induced by DNA damage and stress, is an important TP53 tumor suppressor mechanism [110]. In addition to EMT and invasion, ARID1A and SWI/SNF are implicated in regulating genomic stability, such that ARID1A inactivation is associated with increased DNA damage, but the roles of ARID1A in apoptotic signaling are not well characterized and may be context dependent [8,50,111–113]. Genetic experiments here suggest that TP53 mutant endometrial epithelia display increased caspase-mediated apoptosis, and further ARID1A loss in TP53-ARID1A mutants suppresses cell death. ATF3 has well established ties to apoptosis and cell death regulation despite context-dependency [71,72], and ATF3 activation in ARID1A-deficient endometrial epithelia may suppress pro-apoptotic genes in the absence of TP53. TP63 has also been previously shown to antagonize BCL-2-related, pro-apoptotic transcriptional programs [114]. ATF3 induction and associated TP63 could be a mechanism of how ARID1A loss causes cells to bypass cell death, perhaps explaining why ARID1A mutant tumors are dependent on p53 pathway function, but not TP53 itself. The TNF-mediated extrinsic apoptotic pathway is also regulated by the p53 pathway to some extent but is less characterized [115]. Endoplasmic reticulum (ER) stress related gene sets were the most highly enriched GO terms among ARID1A promoter bound genes *in vivo* endometrial epithelia, and ER stress crosstalk between ARID1A and TP53 could contribute to interrelated transcriptional and cell death regulation [116,117].

The root of mutation mutual exclusivity could be attributed to interconnected transcriptional regulation by ARID1A and TP53. Our *in vivo* binding data revealed that ARID1A interacts with chromatin near TP53 target genes, notably including direct repression of stress-induced transcription factor *Atf3*. ATF3 also has been shown to bind 20–40% of TP53 genomic targets [72,73], suggesting ARID1A loss probably affects multiple aspects of TP53 regulated chromatin. Recently, integrative transcriptomic analyses in mouse tumors and human cell-based models of ARID1A mutant endometrial tumorigenesis have identified the p53 pathway as a key ARID1A-mediated signaling network [118]. Our analyses suggest that ARID1A mutant tumors are dependent on p53 pathway function. In TP53 mutant tumors, ATF3 induction and TP63+ squamous differentiation following ARID1A loss could be a compensatory mechanism that partially restores p53 pathway function. It is worth noting that these genetic mechanisms are difficult to explore in cell culture models, as the majority of immortalized cell lines have impaired p53 signaling, whether that be through inherent mutation [119], selection during culture [120], or immortalization techniques like SV40 large T antigen [121]. In fact, half of the 30 endometrial cancer cell lines profiled by CCLE are TP53/ARID1A co-mutant [122]. This places further emphasis on *in vivo* characterization of TP53-ARID1A functional and genetic relationships.

## Materials and methods

### Ethics statement

Mice were housed at the Van Andel Research Institute (VARI) Animal Facility and the Michigan State University Grand Rapids Research Center in accordance with protocols approved by the Michigan State University Institutional Animal Care and Use Committee (IACUC).

### Mice and animal husbandry

All mice were maintained on an outbred genetic background using CD-1 mice (Charles River). *(Gt)R26*^Pik3ca*H1047R^, *Trp53*^*fl*^, and *LtfCre (Tg(Ltf-iCre)14Mmul)* alleles were purchased from The Jackson Laboratory and confirmed by PCR using published methods [36–38]. Age-matched, CRE-negative (*LtfCre*^*0/0*^) littermates were used as controls. Endpoints were vaginal bleeding, severe abdominal distension, and signs of severe illness, such as dehydration, hunching, jaundice, ruffled fur, signs of infection, or non-responsiveness. Sample sizes within each genotype were chosen based on the proportions of animals with vaginal bleeding between each experimental group or a Kaplan-Meyer log-rank test for survival differences.

### Histology and immunohistochemistry

For indirect immunohistochemistry (IHC), 10% neutral-buffered formalin (NBF)-fixed paraffin sections were processed for heat-based antigen unmasking in 10 mM sodium citrate [pH 6.0], with the exception of ATF3, which used 10 mM Tris-HCl, 1 mM EDTA [pH 9.0]. Sections were incubated with antibodies at the following dilutions: 1:200 ARID1A (D2A8U) (12354, Cell Signaling); 1:400 Phospho-S6 (4585, Cell Signaling); 1:100 KRT8 (TROMA1, DHSB); 1:200 Cleaved Caspase-3 (9579, Cell Signaling); 1:400 Ki67 (12202, Cell Signaling); 1:200 ATF3 (GTX37776, GeneTex); 1:200 TP63 (N2C1, GeneTex); 1:200 TP63 (13109, Cell Signaling); 1:100 COL17A1 (ab184996, abcam). The following Biotin-conjugated secondary antibodies were used: donkey anti-rabbit IgG (711-065-152, Jackson Immuno-research Lab) and donkey anti-rat IgG (#705-065-153, Jackson Immuno-research Lab). Secondary antibodies were detected using VECTASTAIN Elite ABC HRP Kit (Vector). Sections for IHC were lightly counter-stained with Hematoxylin QS or Methyl Green (Vector Labs). Routine Hematoxylin and Eosin (H&E) staining of sections was performed by the VARI Histology and Pathology Core. Adjacent sections were used for H&E and IHC marker comparisons as in Fig 8. At least four animals per genotype were assayed for each histological analysis and immunohistochemical marker.

### Endometrial epithelial cell isolation and RNA-seq

Approximately 76-day old mouse uteri were surgically removed, digested, enriched for EPCAM-positive epithelial cells by magnetic sorting, and purified for RNA as previously described [34]. Mouse libraries (*n* = 3 biological replicates) were prepared and sequenced by the Van Andel Genomics Core from 100 ng of isolated mouse cell total RNA as previously described [34]. For analysis, briefly, raw reads were trimmed and aligned to mm10 assembly and indexed to GENCODE (vM16) via *STAR* [123]. Low count genes with less than one count per sample on average were filtered prior to count normalization and differential gene expression (DGE) analysis by *DESeq2* [124], using a single term model matrix. Differential expression probabilities were corrected for multiple testing by independent hypothesis weighting (IHW) [125] for downstream analyses. Comparisons between TP53/PIK3CA mutant and ARID1A/PIK3CA mutant mouse DGE results and gene sets were initially filtered for genes with transcripts commonly detected in both cell populations.

### Cleavage Under Targets and Release Using Nuclease (CUT&RUN)

EPCAM-positive endometrial epithelial cells were enriched from healthy, adult wild-type mouse uteri as described above, and 100,000 resulting cells were used for each CUT&RUN reaction as previously reported with slight amendments [50,51]. Briefly, Concanavalin A magnetic beads (Bangs) were washed in Binding Buffer (20 mM HEPES-KOH pH 7.9, 10 mM KCl, 1 mM CaCl_2_, 1 mM MnCl_2_) and incubated with either anti-ARID1A (*n* = 2, D2A8U, Cell Signaling) or Rabbit IgG (*n* = 2, 2729, Cell Signaling). Purified cells were washed in Wash Buffer (20 mM HEPES-NaOH pH 7.5, 150 mM NaCl, 0.5 mM spermidine, 5 mM sodium butyrate) then added to the conjugated antibody-bead slurry. After 10 minutes of nutating incubation at ambient temperature, Antibody Buffer (20 mM HEPES-NaOH pH 7.5, 150 mM NaCl, 0.5 mM spermidine, 5 mM sodium butyrate, 2 mM EDTA, 0.05% digitonin) was added to cell bead mixtures, and nuclear permeabilization was confirmed with Trypan blue dye. Reactions were then incubated overnight at 4°C. Reactions were washed with Digitonin Buffer (20 mM HEPES-NaOH pH 7.5, 150 mM NaCl, 0.5 mM spermidine, 5 mM sodium butyrate, 0.05% digitonin) and incubated with pAG-MNase (CUTANA, EpiCypher) for one hour at room temperature, followed by an additional wash in Digitonin Buffer then Low-Salt Rinse Buffer (20 mM HEPES-NaOH pH 7.5, 0.5 mM spermidine, 5 mM sodium butyrate). Calcium Incubation Buffer (3.5 mM HEPES-NaOH pH 7.5, 10 mM CaCl_2_, 0.05% digitonin) was then added to activate the pAG-MNase enzyme, and the reaction was quenched after 3 minutes using EGTA-STOP buffer (170 mM NaCl, 20 mM EGTA, 0.05% digitonin, 20 μg/mL RNase A, 20 μg/mL glycogen, 0.8 pg/mL *Saccharomyces cerevisiae* nucleosomal DNA as spike-in). Fragments were then released into solution at 37°C followed by 5 minutes centrifugation at 16,000 x *g*. Eluted DNA was then purified with the NucleoSpin Gel and PCR Clean-up Kit (Takara).

### Construction and sequencing of CUT&RUN libraries

Libraries for CUT&RUN samples were prepared by the Van Andel Genomics Core from 0.1–0.3 ng of IP material, using the KAPA Hyper Prep Kit (v8.2) (Kapa Biosystems). Prior to PCR amplification, end-repaired and A-tailed DNA fragments were ligated to IDT for Illumina Unique Dual Index adapters (IDT, Coralville, IA USA) at a concentration of 500 nM. Quality and quantity of the finished libraries were assessed using a combination of Agilent DNA High Sensitivity chip (Agilent Technologies, Inc.), QuantiFluor dsDNA System (Promega Corp.), and Kapa Illumina Library Quantification qPCR assays (Kapa Biosystems). Individually indexed libraries were pooled, and 50 bp, paired-end sequencing was performed on an Illumina NovaSeq6000 sequencer using a 100 cycle sequencing kit (Illumina Inc.). Each library was sequenced to an average depth of 50 million reads. Base calling was done by Illumina RTA3 and output was demultiplexed and converted to FastQ format with Illumina Bcl2fastq v1.9.0.

### CUT&RUN bioinformatic analysis

Raw paired-end reads for anti-ARID1A or IgG CUT&RUN were trimmed with *cutadapt* [126] and *Trim Galore*! and analyzed for quality via *FastQC* [127]. Trimmed reads were aligned to mm10 genome assembly with *bowtie2* [128] using flag `—very-sensitive`and filtered for only properly-paired reads with *samtools* [129] using flag `-f 3`. *Picard MarkDuplicates* (http://broadinstitute.github.io/picard/) was used to remove PCR duplicates. For each biological replicate, *MACS2* [130] was used to call ARID1A broad peaks against the respective IgG negative control as input, with FDR < 0.25 threshold. The resulting peaks were repeat-masked by ENCODE blacklist filtering and filtered for non-standard contigs [131]. A naïve replicate-overlapping peak set was constructed by calling peaks on pooled replicates followed by *bedtools intersect* [132] to select for peaks of at least 50% overlap with each biological replicate. *HOMER* [133] was used to annotate peaks, test genomic enrichment, compute motif enrichment, re-center peaks on motifs, and quantify signal profiles across regions of interest. *csaw* [134] was used to count sequencing reads in genomic loci of interest. *IGV* [135] was used for visualizing CUT&RUN and ATAC signal as *MACS2* enrichment log-likelihood (logLR) at mm10 genomic loci.

### Clinical and public cancer data analysis

The Cancer Genome Atlas (TCGA) Pan-Cancer Atlas [30] and UCEC cohort-specific [18] data were utilized in this study. TCGA Pan-Cancer Atlas somatic mutation data were extracted from the MC3 Public MAF (v0.2.8) analysis data set [29]. Clinical data and ARID1A and TP53 alteration incidence rates specifically in endometrial cancer were extracted from the TCGA Pan-Can UCEC cohort (*n*  =  509) retrieved from cBioPortal [136,137]. MSK-IMPACT 2017 data [52] were also retrieved from cBioPortal. Cancer dependency map CRISPR knockout screen data from Achilles (DepMap Public 21Q1) were retrieved from the DepMap portal [53,138,139]. PARADIGM pathway activity inference [49] data for TCGA Pan-Cancer Atlas were retrieved from NCI GDC [140]. For TCGA-UCEC specific molecular analyses, data were retrieved from the 28th January, 2016 release of Broad GDAC Firehose (https://doi.org/10.7908/C11G0KM9). RNASeqV2 RSEM [141] normalized gene counts were further quantile normalized prior to filtering low-count genes (less than one normalized count per sample on average) and fitting linear models via *limma* [142] for differential expression analysis in subsets of patients using a single term model matrix. Empirical Bayes moderated statistics were computed via *limma*::*eBayes* with arguments ‘trend  =  TRUE’ and ‘robust  =  TRUE’, and probabilities were adjusted for multiple testing by Benjamini-Hochberg FDR [143] correction. Only non-silent frameshift, nonsense, or splice site mutations were considered functional ARID1A mutations in UCEC-specific molecular analyses, e.g. GSEA and DGE comparisons. All non-synonymous or non-silent variants were considered as mutations for TP53, PIK3CA, POLE, and all pan-cancer molecular analyses. “Wild-type” samples included synonymous or silent mutations.

### Gene set enrichment analysis

For MSigDB Hallmark pathways [45] and GO Biological Process [144,145] gene set collections (v6.2), Broad GSEA [44] was performed via GenePattern [146] on ortholog-converted *DESeq2* [124] normalized counts from experimental mouse data and RNASeqV2 RSEM [141] normalized counts from TCGA-UCEC data. Manual hypergeometric enrichment tests or *clusterProfiler* enrichment functions were computed on gene sets of interest compared to respective expressed gene universes [147]. Identifying significantly over-represented terms in enriched GO Biological Process gene sets was achieved with a hypergeometric enrichment test framework. Briefly, universes of gene set terms were constructed for single words, word doublets, and word triplets within the 3653 measured gene sets. The number of gene sets containing a given term was then computed for the list of enriched gene sets as well as the respective gene set universe. A hypergeometric enrichment test was then employed to determine if the specific term is over-represented within the respective gene set universe, and this framework was applied to all single word, word doublet, and word triplet terms. For simplified visualization in Fig 4D, results were manually curated to omit some duplicate terms, such as “response to virus” and “to virus”.

### Bioinformatics and statistics

*biomaRt* was used for all gene nomenclature and mouse-human ortholog conversions [148]. The cumulative hypergeometric distribution was calculated in R for enrichment tests. Hierarchical clustering by Euclidean distance and heatmaps were generated by *ComplexHeatmap* [149]. Mouse mm10 genome sequence conservation across vertebrates, computed via *PHAST* [150,151], was extracted from the UCSC browser. *ggplot2* was used for some plots in this study [152]. The statistical language R was used for various computing functions throughout this study [153].

## Supporting information

S1 Fig TP53 mutations in endometrial cancers profiled by TCGA-UCEC. **a**, *PIK3CA* co-alteration rate among *ARID1A* and *TP53* altered UCEC tumors. **b**, Lollipop plot for mutations in *TP53* gene across TCGA-UCEC data (Pan-Can cohort). **c**, Distribution of TP53 mutations by type in TCGA-UCEC serous subtype primary tumors. **d**, Kaplan-Meier overall survival curves for TP53 mutant UCEC tumors segregated by type of TP53 mutation: missense vs. truncating. Statistic is log-rank test. **e**, Distribution of tumor grading among TP53 mutant UCEC tumors segregated by type of TP53 mutation: missense vs. truncating. Statistic is chi-squared test.
(TIF)

S2 Fig Additional histopathological characterization of TP53/PIK3CA mutant mice. **a,** Representative low-magnification H&E histology of control mouse uterus. **b**, Additional representative H&E histology of TP53/PIK3CA mutant uterus (approximately 76-day old) at varying magnifications. Arrowheads depict endometrial epithelia. **c**, KRT8 (left), a marker of endometrial epithelium, and phospho-S6 (right), a marker of activated PI3K signaling. Arrowheads depict mutant endometrial epithelia.
(TIF)

S3 Fig EPCAM endometrial epithelial cell purification statistics. **a**, Example flow cytometry analysis of EPCAM purity from magnetically sorted *LtfCre*^*0/+*^*; (Gt)R26*^Pik3ca*H1047R^*; Trp53*^*fl/fl*^ (TP53/PIK3CA mutant) mouse endometrial epithelial cells. **b**, Purity of EPCAM-isolated cell populations for each sample sequenced by RNA-seq. Mean purity ± SD (%) among sequenced samples was 84.6 ± 6.9. These results are not significantly different from the previously reported control group, 87.7 ± 5.4 (by unpaired, two-tailed *t*-test).
(TIF)

S4 Fig Differences in epithelial-mesenchymal transition following TP53 vs. ARID1A loss. **a**, Broad GSEA waterfall plots for the Hallmark epithelial-mesenchymal transition (EMT) pathway in cells from each genetic mouse model compared to controls. **b**, *k*-means clustering (*k* = 3) of differential gene expression in TP53/PIK3CA mutant and ARID1A/PIK3CA mutant endometrial epithelial cells compared to controls for 183 mouse orthologs within the Hallmark EMT pathway. Red values indicate gene upregulation in mutant cells, and blue values indicate downregulation. **c**, Relative expression box-dot plots summarizing gene expression changes in the *k* clusters for each genetic mouse model compared to control cells. Statistic is unpaired, two-tailed Wilcoxon test. *** *p* < 0.001. **d**, Zoom into cluster 1 genes (*n* = 60) labeled by human ortholog. Red, bolded genes are further displayed in **e** as a box-dot plot. Statistic is FDR as reported by *DESeq2* Wald test: * FDR < 0.05; ** FDR < 0.01; *** FDR < 0.001.
(TIF)

S5 Fig Pathway analysis of distinct expression programs in TP53- and ARID1A-loss-driven hyperplasia. Enrichment for Hallmark pathways, GO Biological Process gene sets, and oncogenic signatures (all retrieved from MSigDB) among genes DE between TP53/PIK3CA mutant vs. ARID1A/PIK3CA mutant endometrial epithelial cells, separated by directionality.
(TIF)

S6 Fig Pathway alterations driven by TP53 and ARID1A mutations are associated with histological subtype. **a**, Broad GSEA results for Hallmark pathways between TCGA-UCEC tumors: ARID1A mutant / TP53 wild-type vs. wild-type / wild-type compared to TP53 mutant / ARID1A wild-type vs. wild-type / wild-type. **b**, Broad GSEA results for Hallmark pathways between TCGA-UCEC tumors: ARID1A mutant / TP53 wild-type vs. TP53 mutant / ARID1A wild-type compared to endometrioid vs. serous. Significant correlation of pathway enrichment is observed between genetics and subtype by Pearson (*r*) and Spearman (*r*_s_) correlations. **c**, same as in **b** but for GO Biological Process gene sets. **d**, Top, phi correlation and associated statistic of dependent samples classified as either endometrioid vs. serous histotype and TP53mut/ARID1Awt or ARID1Amut/TP53wt. Bottom, Fisher’s Z-transformations comparing the Pearson correlation coefficients between the phi correlation and GSEA results.
(TIF)

S7 Fig GO Biological Process gene set overlaps between mouse and human genotypes. Overlap of enriched gene sets (|NES| > 1) determined in Fig 4 GSEA for various mouse and human genetic comparisons as displayed, further segregated by upregulated vs. downregulated gene sets. Significant overlap indicates that more enriched gene sets were observed in both comparisons than expected by chance alone. Statistic is hypergeometric enrichment.
(TIF)

S8 Fig Differential gene expression analysis of TP53 vs. ARID1A mutant mouse and human samples. **a**, 81 genes with significantly higher expression (human: *limma* FDR < 0.05; mouse: *DESeq2* FDR < 0.05) in TP53 mutant samples compared to ARID1A mutants. **b**, as in **a** but for 149 genes with higher expression in ARID1A mutant samples compared to TP53 mutants. **c**, top 10 enriched Hallmark pathways and GO Biological Process gene sets among the 81 genes identified in **a**. Gray text indicates non-significance (FDR > 0.05). **d**, as in **c** but for genes identified in **b**.
(TIF)

S9 Fig Extended GSEA results for disease and model genetic comparisons. **a**, Detailed Broad GSEA results for TCGA-UCEC ARID1A mutant / TP53 wild-type vs. wild-type / wild-type compared to TP53 mutant / ARID1A wild-type vs. wild-type / wild-type. Representative examples of highly enriched gene sets are labeled for each quadrant. **b**, Significantly over-represented terms in enriched gene sets (|NES| > 1) highlighted in **a**. Statistic is hypergeometric enrichment. See methods for analysis framework. **c**-**d**, Same as in **a**-**b** but for TP53/PIK3CA mutant vs. control cells compared to ARID1A/PIK3CA mutant vs. control cells.
(TIF)

S10 Fig Enrichment of gene expression alterations at TP53 core transcriptional program genes in mouse models. Proportion of genes significantly differentially expressed (DE) in TP53 core transcriptional program gene mouse orthologs compared to all expressed genes, for (top) ARID1A/PIK3CA mutant and (bottom) TP53/PIK3CA mutant endometrial epithelia compared to cells from control mice. Statistic is hypergeometric enrichment test.
(TIF)

S11 Fig PARADIGM pathway activity associated with ARID1A mutation in UCEC tumors. **a**, Distribution of PARADIGM score differences between ARID1A mutant (*n* = 174) vs. wild-type (*n* = 128) TCGA-UCEC tumors, considerate of only TP53 wild-type tumors, as in Fig 5C. Top, all 19,503 measured pathways; bottom, the 36 pathways with keyword “p53”. **b**, Empirical distribution of mean differences of ARID1A mutant vs. wild-type PARADIGM scores, based on 50,000 samples of 36 random PARADIGM pathways, as in Fig 5D. The blue line represents the mean score difference for the 36 pathways with keyword “p53” with associated permutation statistic.
(TIF)

S12 Fig Extended analysis of ARID1A binding sites *in vivo* endometrial epithelia. **a**, Genomic feature enrichment among 2146 ARID1A *in vivo* binding sites. **b**, Top significant (*p* < 10^−30^) known motifs from *HOMER* sequence analysis of ARID1A *in vivo* binding sites compared to the background genome. Motif sequence logos are scaled by information content for each nucleotide base. **c**, Overlap of ARID1A/PIK3CA mutant DE genes (RNA-seq, FDR < 0.05, *n* = 3481) and genes with ARID1A binding detected within 50 kb of TSS. **d**, Top 10 (left) GO Biological Process gene sets and (right) Hallmark pathways enriched among 2887 human orthologs of genes with ARID1A binding within 50 kb from TSS.
(TIF)

S13 Fig Additional TCGA-UCEC and MSK-IMPACT analysis of TP53/ARID1A co-altered tumors. **a**, Distribution of histologic grading among TCGA-UCEC primary tumors, segregated by TP53/ARID1A co-altered (*n* = 38) vs. else (*n* = 471). Statistic is two-tailed Fisher’s exact test. **b**, Frequency of mixed morphology subtype tumors across all TCGA-UCEC primary tumors (*n* = 509) compared to specifically TP53/ARID1A co-altered tumors (*n* = 38). Statistic is hypergeometric enrichment. **c**, Contingency table of *TP53*/*ARID1A* co-mutation rate in all primary vs. metastatic tumors from the MSK-IMPACT Clinical Sequencing Cohort. Statistic is one-tailed Fisher’s exact test. **d**, Contingency table of ARID1A co-mutation rate in TP53 mutant primary vs. metastatic tumors from MSK-IMPACT as in **c**. **e**, *TP53*/*ARID1A* co-mutation rates of all MSK-IMPACT tumor samples segregated by presence or absence of *POLE* mutations. Statistic is two-tailed Fisher’s exact test.
(TIF)

S14 Fig TP53/ARID1A/PIK3CA mutant mouse survival and marker staining. **a**, Survival curves for *LtfCre*^*0/+*^*; (Gt)R26*^Pik3ca*H1047R^*; Trp53*^*fl/fl*^ mice with and without an additional *Arid1a*^*fl/fl*^ allele (TP53/PIK3CA mutant and TP53/ARID1A/PIK3CA mutant mice). Statistic is Cox log-rank test. **b**, Representative IHC marker analysis in TP53/ARID1A/PIK3CA mutant mice. Left, phospho-S6; right, ARID1A staining. Arrowheads denote mutant endometrial epithelia.
(TIF)

S15 Fig Representative proliferation and caspase-mediated cell death marker IHC. More representative IHC images of Ki67 (proliferation) and cleaved caspase-3 (cell death) in TP53/PIK3CA mutant (**a** and **c**) and TP53/ARID1A/PIK3CA mutant (**b** and **d**) mouse uterus, respectively. Arrowheads denote mutant endometrial epithelia.
(TIF)

S16 Fig ARID1A loss-induced ATF3 and TP63 is associated with invasive transcriptional signatures. **a**, *Atf3* gene expression (linear) in mutant mouse endometrial epithelial cell RNA-seq data. Statistic is FDR as reported by *DESeq2*. **b**, Significant correlation of *Trp63* and *Snai2* gene expression in experimental mouse endometrial epithelial cells. Expression is quantified as log_2_(normalized counts + 1). Statistics are Pearson (*r*) and Spearman (*r*_*s*_) coefficients. **c**, Workflow for identification of the TP63 target gene network induced by ARID1A mutation (*n* = 30 genes), beginning with 180 high confidence TP63 target genes defined by Riege *et al*. [79]. **d**, RNA-seq relative expression heatmap for genetic mouse models and UCEC primary tumor samples for the TP63 target gene network induced by ARID1A mutation. Red, bolded genes display similar expression patterns in genetic mouse models and human UCEC tumors. **e**, Top GO Biological Process gene sets enriched (hypergeometric enrichment *p* < 0.001) in the TP63 target gene network induced by ARID1A mutation. # represents the number of target genes found within each gene set. **f**, Broad GSEA results for the TP63 target gene network induced by ARID1A mutation among human UCEC primary tumors segregated by ARID1A and TP53 genetic status. Significant enrichment was observed in ARID1Amut/TP53wt vs. wt/wt tumors.
(TIF)

S17 Fig Further representative marker staining of mutant mouse models and vaginal pseudostratified squamous epithelium. **a**, Representative uterine TP63 (Cell Signaling) staining in mutant mouse models. Arrowheads denote endometrial epithelium. **b**-**c**, Further representative uterine (**b**) TP63 (GeneTex) and (**c**) COL17A1 staining in TP53/ARID1A/PIK3CA mutant mice. Arrowheads denote mutant endometrial epithelia. **d**, Representative H&E and IHC staining for ATF3, TP63 (Cell Signaling), and COL17A1 in vaginal pseudostratified squamous epithelium of wild-type CD-1 mice. Arrowheads denote basal epithelial cells expressing markers.
(TIF)

## References

[1] Lawrence MS, Stojanov P, Mermel CH, Robinson JT, Garraway LA, Golub TR, et al. Discovery and saturation analysis of cancer genes across 21 tumour types. Nature. 2014;505(7484):495–501. Epub 2014/01/07. doi: 10.1038/nature12912 ; PubMed Central PMCID: PMC4048962. 24390350 PMC4048962

[2] Lane DP, Crawford LV. T antigen is bound to a host protein in SV40-transformed cells. Nature. 1979;278(5701):261–3. Epub 1979/03/15. doi: 10.1038/278261a0 . 218111

[3] Kastenhuber ER, Lowe SW. Putting p53 in Context. Cell. 2017;170(6):1062–78. Epub 2017/09/09. doi: 10.1016/j.cell.2017.08.028 ; PubMed Central PMCID: PMC5743327. 28886379 PMC5743327

[4] Wu JN, Roberts CW. ARID1A mutations in cancer: another epigenetic tumor suppressor? Cancer Discov. 2013;3(1):35–43. Epub 2012/12/05. doi: 10.1158/2159-8290.CD-12-0361 ; PubMed Central PMCID: PMC3546152. 23208470 PMC3546152

[5] Kadoch C, Crabtree GR. Mammalian SWI/SNF chromatin remodeling complexes and cancer: Mechanistic insights gained from human genomics. Sci Adv. 2015;1(5):e1500447. Epub 2015/11/26. doi: 10.1126/sciadv.1500447 ; PubMed Central PMCID: PMC4640607. 26601204 PMC4640607

[6] Sullivan KD, Galbraith MD, Andrysik Z, Espinosa JM. Mechanisms of transcriptional regulation by p53. Cell Death Differ. 2018;25(1):133–43. Epub 2017/11/11. doi: 10.1038/cdd.2017.174 ; PubMed Central PMCID: PMC5729533. 29125602 PMC5729533

[7] Nagl NG Jr., Patsialou A, Haines DS, Dallas PB, Beck GR Jr., Moran E. The p270 (ARID1A/SMARCF1) subunit of mammalian SWI/SNF-related complexes is essential for normal cell cycle arrest. Cancer Res. 2005;65(20):9236–44. Epub 2005/10/19. doi: 10.1158/0008-5472.CAN-05-1225 . 16230384

[8] Shen J, Peng Y, Wei L, Zhang W, Yang L, Lan L, et al. ARID1A Deficiency Impairs the DNA Damage Checkpoint and Sensitizes Cells to PARP Inhibitors. Cancer Discov. 2015;5(7):752–67. Epub 2015/06/13. doi: 10.1158/2159-8290.CD-14-0849 ; PubMed Central PMCID: PMC4497871. 26069190 PMC4497871

[9] Williams AB, Schumacher B. p53 in the DNA-Damage-Repair Process. Cold Spring Harb Perspect Med. 2016;6(5). Epub 2016/04/07. doi: 10.1101/cshperspect.a026070 ; PubMed Central PMCID: PMC4852800. 27048304 PMC4852800

[10] Shaw PH. The role of p53 in cell cycle regulation. Pathol Res Pract. 1996;192(7):669–75. Epub 1996/07/01. doi: 10.1016/S0344-0338(96)80088-4 . 8880867

[11] Berchuck A, Kohler MF, Marks JR, Wiseman R, Boyd J, Bast RC Jr., The p53 tumor suppressor gene frequently is altered in gynecologic cancers. Am J Obstet Gynecol. 1994;170(1 Pt 1):246–52. Epub 1994/01/01. doi: 10.1016/s0002-9378(94)70414-7 . 8296829

[12] Berger AC, Korkut A, Kanchi RS, Hegde AM, Lenoir W, Liu W, et al. A Comprehensive Pan-Cancer Molecular Study of Gynecologic and Breast Cancers. Cancer Cell. 2018;33(4):690–705 e9. Epub 2018/04/07. doi: 10.1016/j.ccell.2018.03.014 ; PubMed Central PMCID: PMC5959730. 29622464 PMC5959730

[13] Wiegand KC, Shah SP, Al-Agha OM, Zhao Y, Tse K, Zeng T, et al. ARID1A mutations in endometriosis-associated ovarian carcinomas. N Engl J Med. 2010;363(16):1532–43. Epub 2010/10/15. doi: 10.1056/NEJMoa1008433 ; PubMed Central PMCID: PMC2976679. 20942669 PMC2976679

[14] Jones S, Wang TL, Shih Ie M, Mao TL, Nakayama K, Roden R, et al. Frequent mutations of chromatin remodeling gene ARID1A in ovarian clear cell carcinoma. Science. 2010;330(6001):228–31. Epub 2010/09/10. doi: 10.1126/science.1196333 ; PubMed Central PMCID: PMC3076894. 20826764 PMC3076894

[15] Le Gallo M, O’Hara AJ, Rudd ML, Urick ME, Hansen NF, O’Neil NJ, et al. Exome sequencing of serous endometrial tumors identifies recurrent somatic mutations in chromatin-remodeling and ubiquitin ligase complex genes. Nat Genet. 2012;44(12):1310–5. Epub 2012/10/30. doi: 10.1038/ng.2455 ; PubMed Central PMCID: PMC3515204. 23104009 PMC3515204

[16] Le Gallo M, Rudd ML, Urick ME, Hansen NF, Zhang S, Program NCS, et al. Somatic mutation profiles of clear cell endometrial tumors revealed by whole exome and targeted gene sequencing. Cancer. 2017;123(17):3261–8. Epub 2017/05/10. doi: 10.1002/cncr.30745 ; PubMed Central PMCID: PMC5587124. 28485815 PMC5587124

[17] Okuda T, Otsuka J, Sekizawa A, Saito H, Makino R, Kushima M, et al. p53 mutations and overexpression affect prognosis of ovarian endometrioid cancer but not clear cell cancer. Gynecol Oncol. 2003;88(3):318–25. Epub 2003/03/22. doi: 10.1016/s0090-8258(02)00149-x . 12648581

[18] Cancer Genome Atlas Research N, Kandoth C, Schultz N, Cherniack AD, Akbani R, Liu Y, et al. Integrated genomic characterization of endometrial carcinoma. Nature. 2013;497(7447):67–73. Epub 2013/05/03. doi: 10.1038/nature12113 ; PubMed Central PMCID: PMC3704730. 23636398 PMC3704730

[19] Tashiro H, Isacson C, Levine R, Kurman RJ, Cho KR, Hedrick L. p53 gene mutations are common in uterine serous carcinoma and occur early in their pathogenesis. Am J Pathol. 1997;150(1):177–85. Epub 1997/01/01. ; PubMed Central PMCID: PMC1858541. 9006334 PMC1858541

[20] Cherniack AD, Shen H, Walter V, Stewart C, Murray BA, Bowlby R, et al. Integrated Molecular Characterization of Uterine Carcinosarcoma. Cancer Cell. 2017;31(3):411–23. Epub 2017/03/16. doi: 10.1016/j.ccell.2017.02.010 ; PubMed Central PMCID: PMC5599133. 28292439 PMC5599133

[21] Cancer Genome Atlas Research N. Integrated genomic analyses of ovarian carcinoma. Nature. 2011;474(7353):609–15. Epub 2011/07/02. doi: 10.1038/nature10166 ; PubMed Central PMCID: PMC3163504. 21720365 PMC3163504

[22] Guan B, Wang TL, Shih Ie M. ARID1A, a factor that promotes formation of SWI/SNF-mediated chromatin remodeling, is a tumor suppressor in gynecologic cancers. Cancer Res. 2011;71(21):6718–27. Epub 2011/09/09. doi: 10.1158/0008-5472.CAN-11-1562 ; PubMed Central PMCID: PMC3206175. 21900401 PMC3206175

[23] Streppel MM, Lata S, DelaBastide M, Montgomery EA, Wang JS, Canto MI, et al. Next-generation sequencing of endoscopic biopsies identifies ARID1A as a tumor-suppressor gene in Barrett’s esophagus. Oncogene. 2014;33(3):347–57. Epub 2013/01/16. doi: 10.1038/onc.2012.586 ; PubMed Central PMCID: PMC3805724. 23318448 PMC3805724

[24] Cho HD, Lee JE, Jung HY, Oh MH, Lee JH, Jang SH, et al. Loss of Tumor Suppressor ARID1A Protein Expression Correlates with Poor Prognosis in Patients with Primary Breast Cancer. J Breast Cancer. 2015;18(4):339–46. Epub 2016/01/16. doi: 10.4048/jbc.2015.18.4.339 ; PubMed Central PMCID: PMC4705085. 26770240 PMC4705085

[25] Wang K, Kan J, Yuen ST, Shi ST, Chu KM, Law S, et al. Exome sequencing identifies frequent mutation of ARID1A in molecular subtypes of gastric cancer. Nat Genet. 2011;43(12):1219–23. Epub 2011/11/01. doi: 10.1038/ng.982 . 22037554

[26] Zang ZJ, Cutcutache I, Poon SL, Zhang SL, McPherson JR, Tao J, et al. Exome sequencing of gastric adenocarcinoma identifies recurrent somatic mutations in cell adhesion and chromatin remodeling genes. Nat Genet. 2012;44(5):570–4. Epub 2012/04/10. doi: 10.1038/ng.2246 . 22484628

[27] Allo G, Bernardini MQ, Wu RC, Shih Ie M, Kalloger S, Pollett A, et al. ARID1A loss correlates with mismatch repair deficiency and intact p53 expression in high-grade endometrial carcinomas. Mod Pathol. 2014;27(2):255–61. Epub 2013/07/28. doi: 10.1038/modpathol.2013.144 ; PubMed Central PMCID: PMC4603563. 23887303 PMC4603563

[28] Bosse T, ter Haar NT, Seeber LM, v Diest PJ, Hes FJ, Vasen HF, et al. Loss of ARID1A expression and its relationship with PI3K-Akt pathway alterations, TP53 and microsatellite instability in endometrial cancer. Mod Pathol. 2013;26(11):1525–35. Epub 2013/05/25. doi: 10.1038/modpathol.2013.96 . 23702729

[29] Ellrott K, Bailey MH, Saksena G, Covington KR, Kandoth C, Stewart C, et al. Scalable Open Science Approach for Mutation Calling of Tumor Exomes Using Multiple Genomic Pipelines. Cell Syst. 2018;6(3):271–81 e7. Epub 2018/03/30. doi: 10.1016/j.cels.2018.03.002 ; PubMed Central PMCID: PMC6075717. 29596782 PMC6075717

[30] Hoadley KA, Yau C, Hinoue T, Wolf DM, Lazar AJ, Drill E, et al. Cell-of-Origin Patterns Dominate the Molecular Classification of 10,000 Tumors from 33 Types of Cancer. Cell. 2018;173(2):291–304 e6. Epub 2018/04/07. doi: 10.1016/j.cell.2018.03.022 ; PubMed Central PMCID: PMC5957518. 29625048 PMC5957518

[31] Morice P, Leary A, Creutzberg C, Abu-Rustum N, Darai E. Endometrial cancer. Lancet. 2016;387(10023):1094–108. Epub 2015/09/12. doi: 10.1016/S0140-6736(15)00130-0 . 26354523

[32] Urick ME, Bell DW. Clinical actionability of molecular targets in endometrial cancer. Nat Rev Cancer. 2019;19(9):510–21. Epub 2019/08/08. doi: 10.1038/s41568-019-0177-x ; PubMed Central PMCID: PMC7446243. 31388127 PMC7446243

[33] Cancer Genome Atlas Research Network, Kandoth C, Schultz N, Cherniack AD, Akbani R, Liu Y, et al. Integrated genomic characterization of endometrial carcinoma. Nature. 2013;497(7447):67–73. Epub 2013/05/03. doi: 10.1038/nature12113 ; PubMed Central PMCID: PMC3704730. 23636398 PMC3704730

[34] Wilson MR, Reske JJ, Holladay J, Wilber GE, Rhodes M, Koeman J, et al. ARID1A and PI3-kinase pathway mutations in the endometrium drive epithelial transdifferentiation and collective invasion. Nat Commun. 2019;10(1):3554. Epub 2019/08/09. doi: 10.1038/s41467-019-11403-6 ; PubMed Central PMCID: PMC6686004. 31391455 PMC6686004

[35] Wilson MR, Holladay J, Chandler RL. A mouse model of endometriosis mimicking the natural spread of invasive endometrium. Hum Reprod. 2020;35(1):58–69. Epub 2019/12/31. doi: 10.1093/humrep/dez253 . 31886851 PMC8205619

[36] Marino S, Vooijs M, van Der Gulden H, Jonkers J, Berns A. Induction of medulloblastomas in p53-null mutant mice by somatic inactivation of Rb in the external granular layer cells of the cerebellum. Genes Dev. 2000;14(8):994–1004. Epub 2000/04/27. ; PubMed Central PMCID: PMC316543. 10783170 PMC316543

[37] Adams JR, Xu K, Liu JC, Agamez NM, Loch AJ, Wong RG, et al. Cooperation between Pik3ca and p53 mutations in mouse mammary tumor formation. Cancer Res. 2011;71(7):2706–17. Epub 2011/02/18. doi: 10.1158/0008-5472.CAN-10-0738 . 21324922

[38] Daikoku T, Ogawa Y, Terakawa J, Ogawa A, DeFalco T, Dey SK. Lactoferrin-iCre: a new mouse line to study uterine epithelial gene function. Endocrinology. 2014;155(7):2718–24. Epub 2014/05/16. doi: 10.1210/en.2014-1265 ; PubMed Central PMCID: PMC4060188. 24823394 PMC4060188

[39] Hendrickson M, Ross J, Eifel PJ, Cox RS, Martinez A, Kempson R. Adenocarcinoma of the endometrium: analysis of 256 cases with carcinoma limited to the uterine corpus. Pathology review and analysis of prognostic variables. Gynecol Oncol. 1982;13(3):373–92. Epub 1982/06/01. doi: 10.1016/0090-8258(82)90076-2 . 6284595

[40] Murali R, Soslow RA, Weigelt B. Classification of endometrial carcinoma: more than two types. Lancet Oncol. 2014;15(7):e268–78. Epub 2014/05/30. doi: 10.1016/S1470-2045(13)70591-6 . 24872110

[41] Soslow RA, Pirog E, Isacson C. Endometrial intraepithelial carcinoma with associated peritoneal carcinomatosis. Am J Surg Pathol. 2000;24(5):726–32. Epub 2000/05/09. doi: 10.1097/00000478-200005000-00012 . 10800992

[42] Pathiraja P, Dhar S, Haldar K. Serous endometrial intraepithelial carcinoma: a case series and literature review. Cancer Manag Res. 2013;5:117–22. Epub 2013/07/19. doi: 10.2147/CMAR.S45141 ; PubMed Central PMCID: PMC3704304. 23861597 PMC3704304

[43] Ambros RA, Sherman ME, Zahn CM, Bitterman P, Kurman RJ. Endometrial intraepithelial carcinoma: a distinctive lesion specifically associated with tumors displaying serous differentiation. Hum Pathol. 1995;26(11):1260–7. Epub 1995/11/01. doi: 10.1016/0046-8177(95)90203-1 . 7590702

[44] Subramanian A, Tamayo P, Mootha VK, Mukherjee S, Ebert BL, Gillette MA, et al. Gene set enrichment analysis: a knowledge-based approach for interpreting genome-wide expression profiles. Proc Natl Acad Sci U S A. 2005;102(43):15545–50. Epub 2005/10/04. doi: 10.1073/pnas.0506580102 ; PubMed Central PMCID: PMC1239896. 16199517 PMC1239896

[45] Liberzon A, Birger C, Thorvaldsdottir H, Ghandi M, Mesirov JP, Tamayo P. The Molecular Signatures Database (MSigDB) hallmark gene set collection. Cell Syst. 2015;1(6):417–25. Epub 2016/01/16. doi: 10.1016/j.cels.2015.12.004 ; PubMed Central PMCID: PMC4707969. 26771021 PMC4707969

[46] Zhou W, Gross KM, Kuperwasser C. Molecular regulation of Snai2 in development and disease. J Cell Sci. 2019;132(23). Epub 2019/12/04. doi: 10.1242/jcs.235127 . 31792043 PMC12233911

[47] Andrysik Z, Galbraith MD, Guarnieri AL, Zaccara S, Sullivan KD, Pandey A, et al. Identification of a core TP53 transcriptional program with highly distributed tumor suppressive activity. Genome Res. 2017;27(10):1645–57. Epub 2017/09/15. doi: 10.1101/gr.220533.117 ; PubMed Central PMCID: PMC5630028. 28904012 PMC5630028

[48] Kawase T, Ohki R, Shibata T, Tsutsumi S, Kamimura N, Inazawa J, et al. PH domain-only protein PHLDA3 is a p53-regulated repressor of Akt. Cell. 2009;136(3):535–50. Epub 2009/02/11. doi: 10.1016/j.cell.2008.12.002 . 19203586

[49] Vaske CJ, Benz SC, Sanborn JZ, Earl D, Szeto C, Zhu J, et al. Inference of patient-specific pathway activities from multi-dimensional cancer genomics data using PARADIGM. Bioinformatics. 2010;26(12):i237–45. Epub 2010/06/10. doi: 10.1093/bioinformatics/btq182 ; PubMed Central PMCID: PMC2881367. 20529912 PMC2881367

[50] Wilson MR, Reske JJ, Holladay J, Neupane S, Ngo J, Cuthrell N, et al. ARID1A Mutations Promote P300-Dependent Endometrial Invasion through Super-Enhancer Hyperacetylation. Cell Rep. 2020;33(6):108366. Epub 2020/11/12. doi: 10.1016/j.celrep.2020.108366 ; PubMed Central PMCID: PMC7682620. 33176148 PMC7682620

[51] Skene PJ, Henikoff S. An efficient targeted nuclease strategy for high-resolution mapping of DNA binding sites. Elife. 2017;6. Epub 2017/01/13. doi: 10.7554/eLife.21856 ; PubMed Central PMCID: PMC5310842. 28079019 PMC5310842

[52] Zehir A, Benayed R, Shah RH, Syed A, Middha S, Kim HR, et al. Mutational landscape of metastatic cancer revealed from prospective clinical sequencing of 10,000 patients. Nat Med. 2017;23(6):703–13. Epub 2017/05/10. doi: 10.1038/nm.4333 ; PubMed Central PMCID: PMC5461196. 28481359 PMC5461196

[53] Dempster JM, Rossen J, Kazachkova M, Pan J, Kugener G, Root DE, et al. Extracting Biological Insights from the Project Achilles Genome-Scale CRISPR Screens in Cancer Cell Lines. bioRxiv. 2019. 10.1101/720243.

[54] Sun X, Wang SC, Wei Y, Luo X, Jia Y, Li L, et al. Arid1a Has Context-Dependent Oncogenic and Tumor Suppressor Functions in Liver Cancer. Cancer Cell. 2017;32(5):574–89 e6. Epub 2017/11/15. doi: 10.1016/j.ccell.2017.10.007 ; PubMed Central PMCID: PMC5728182. 29136504 PMC5728182

[55] Xu N, Wang L, Sun P, Xu S, Fu S, Sun Z. Low Arid1a Expression Correlates with Poor Prognosis and Promotes Cell Proliferation and Metastasis in Osteosarcoma. Pathol Oncol Res. 2019;25(3):875–81. Epub 2017/11/28. doi: 10.1007/s12253-017-0338-8 . 29177569

[56] Namjan A, Techasen A, Loilome W, Sa-Ngaimwibool P, Jusakul A. ARID1A alterations and their clinical significance in cholangiocarcinoma. PeerJ. 2020;8:e10464. Epub 2020/12/22. doi: 10.7717/peerj.10464 ; PubMed Central PMCID: PMC7719290. 33344089 PMC7719290

[57] Yates LR, Knappskog S, Wedge D, Farmery JHR, Gonzalez S, Martincorena I, et al. Genomic Evolution of Breast Cancer Metastasis and Relapse. Cancer Cell. 2017;32(2):169–84 e7. Epub 2017/08/16. doi: 10.1016/j.ccell.2017.07.005 ; PubMed Central PMCID: PMC5559645. 28810143 PMC5559645

[58] Wei XL, Wang DS, Xi SY, Wu WJ, Chen DL, Zeng ZL, et al. Clinicopathologic and prognostic relevance of ARID1A protein loss in colorectal cancer. World J Gastroenterol. 2014;20(48):18404–12. Epub 2015/01/07. doi: 10.3748/wjg.v20.i48.18404 ; PubMed Central PMCID: PMC4277979. 25561809 PMC4277979

[59] Gibson WJ, Hoivik EA, Halle MK, Taylor-Weiner A, Cherniack AD, Berg A, et al. The genomic landscape and evolution of endometrial carcinoma progression and abdominopelvic metastasis. Nat Genet. 2016;48(8):848–55. Epub 2016/06/28. doi: 10.1038/ng.3602 ; PubMed Central PMCID: PMC4963271. 27348297 PMC4963271

[60] Hai T, Wolfgang CD, Marsee DK, Allen AE, Sivaprasad U. ATF3 and stress responses. Gene Expr. 1999;7(4–6):321–35. Epub 1999/08/10. ; PubMed Central PMCID: PMC6174666. 10440233 PMC6174666

[61] Yin X, Dewille JW, Hai T. A potential dichotomous role of ATF3, an adaptive-response gene, in cancer development. Oncogene. 2008;27(15):2118–27. Epub 2007/10/24. doi: 10.1038/sj.onc.1210861 . 17952119

[62] Wu X, Nguyen BC, Dziunycz P, Chang S, Brooks Y, Lefort K, et al. Opposing roles for calcineurin and ATF3 in squamous skin cancer. Nature. 2010;465(7296):368–72. Epub 2010/05/21. doi: 10.1038/nature08996 ; PubMed Central PMCID: PMC3050632. 20485437 PMC3050632

[63] Zhang C, Zhang X, Huang L, Guan Y, Huang X, Tian XL, et al. ATF3 drives senescence by reconstructing accessible chromatin profiles. Aging Cell. 2021;20(3):e13315. Epub 2021/02/05. doi: 10.1111/acel.13315 ; PubMed Central PMCID: PMC7963335. 33539668 PMC7963335

[64] Sharma K, Vu TT, Cook W, Naseri M, Zhan K, Nakajima W, et al. p53-independent Noxa induction by cisplatin is regulated by ATF3/ATF4 in head and neck squamous cell carcinoma cells. Mol Oncol. 2018;12(6):788–98. Epub 2018/01/21. doi: 10.1002/1878-0261.12172 ; PubMed Central PMCID: PMC5983129. 29352505 PMC5983129

[65] Hartman MG, Lu D, Kim ML, Kociba GJ, Shukri T, Buteau J, et al. Role for activating transcription factor 3 in stress-induced beta-cell apoptosis. Mol Cell Biol. 2004;24(13):5721–32. Epub 2004/06/17. doi: 10.1128/MCB.24.13.5721-5732.2004 ; PubMed Central PMCID: PMC480886. 15199129 PMC480886

[66] Borgoni S, Sofyali E, Soleimani M, Wilhelm H, Muller-Decker K, Will R, et al. Time-Resolved Profiling Reveals ATF3 as a Novel Mediator of Endocrine Resistance in Breast Cancer. Cancers (Basel). 2020;12(10). Epub 2020/10/15. doi: 10.3390/cancers12102918 ; PubMed Central PMCID: PMC7650760. 33050633 PMC7650760

[67] Xie JJ, Xie YM, Chen B, Pan F, Guo JC, Zhao Q, et al. ATF3 functions as a novel tumor suppressor with prognostic significance in esophageal squamous cell carcinoma. Oncotarget. 2014;5(18):8569–82. Epub 2014/08/26. doi: 10.18632/oncotarget.2322 ; PubMed Central PMCID: PMC4226705. 25149542 PMC4226705

[68] Wang A, Arantes S, Yan L, Kiguchi K, McArthur MJ, Sahin A, et al. The transcription factor ATF3 acts as an oncogene in mouse mammary tumorigenesis. BMC Cancer. 2008;8:268. Epub 2008/09/24. doi: 10.1186/1471-2407-8-268 ; PubMed Central PMCID: PMC2564979. 18808719 PMC2564979

[69] Azizi N, Toma J, Martin M, Khalid MF, Mousavi F, Win PW, et al. Loss of activating transcription factor 3 prevents KRAS-mediated pancreatic cancer. Oncogene. 2021;40(17):3118–35. Epub 2021/04/18. doi: 10.1038/s41388-021-01771-z ; PubMed Central PMCID: PMC8173475. 33864001 PMC8173475

[70] Taketani K, Kawauchi J, Tanaka-Okamoto M, Ishizaki H, Tanaka Y, Sakai T, et al. Key role of ATF3 in p53-dependent DR5 induction upon DNA damage of human colon cancer cells. Oncogene. 2012;31(17):2210–21. Epub 2011/09/20. doi: 10.1038/onc.2011.397 . 21927023

[71] Lu D, Wolfgang CD, Hai T. Activating transcription factor 3, a stress-inducible gene, suppresses Ras-stimulated tumorigenesis. J Biol Chem. 2006;281(15):10473–81. Epub 2006/02/14. doi: 10.1074/jbc.M509278200 . 16469745

[72] Tanaka Y, Nakamura A, Morioka MS, Inoue S, Tamamori-Adachi M, Yamada K, et al. Systems analysis of ATF3 in stress response and cancer reveals opposing effects on pro-apoptotic genes in p53 pathway. PLoS One. 2011;6(10):e26848. Epub 2011/11/03. doi: 10.1371/journal.pone.0026848 ; PubMed Central PMCID: PMC3202577. 22046379 PMC3202577

[73] Zhao J, Li X, Guo M, Yu J, Yan C. The common stress responsive transcription factor ATF3 binds genomic sites enriched with p300 and H3K27ac for transcriptional regulation. BMC Genomics. 2016;17:335. Epub 2016/05/06. doi: 10.1186/s12864-016-2664-8 ; PubMed Central PMCID: PMC4857411. 27146783 PMC4857411

[74] Li X, Guo M, Cai L, Du T, Liu Y, Ding HF, et al. Competitive ubiquitination activates the tumor suppressor p53. Cell Death Differ. 2020;27(6):1807–18. Epub 2019/12/05. doi: 10.1038/s41418-019-0463-x ; PubMed Central PMCID: PMC7244561. 31796886 PMC7244561

[75] Hai T, Curran T. Cross-family dimerization of transcription factors Fos/Jun and ATF/CREB alters DNA binding specificity. Proc Natl Acad Sci U S A. 1991;88(9):3720–4. Epub 1991/05/01. doi: 10.1073/pnas.88.9.3720 ; PubMed Central PMCID: PMC51524. 1827203 PMC51524

[76] Xu L, Zu T, Li T, Li M, Mi J, Bai F, et al. ATF3 downmodulates its new targets IFI6 and IFI27 to suppress the growth and migration of tongue squamous cell carcinoma cells. PLoS Genet. 2021;17(2):e1009283. Epub 2021/02/05. doi: 10.1371/journal.pgen.1009283 ; PubMed Central PMCID: PMC7888615. 33539340 PMC7888615

[77] Shi B, Yan W, Liu G, Guo Y. MicroRNA-488 inhibits tongue squamous carcinoma cell invasion and EMT by directly targeting ATF3. Cell Mol Biol Lett. 2018;23:28. Epub 2018/06/28. doi: 10.1186/s11658-018-0094-0 ; PubMed Central PMCID: PMC6006839. 29946339 PMC6006839

[78] Wang A, Arantes S, Conti C, McArthur M, Aldaz CM, MacLeod MC. Epidermal hyperplasia and oral carcinoma in mice overexpressing the transcription factor ATF3 in basal epithelial cells. Mol Carcinog. 2007;46(6):476–87. Epub 2007/02/14. doi: 10.1002/mc.20298 . 17295236

[79] Riege K, Kretzmer H, Sahm A, McDade SS, Hoffmann S, Fischer M. Dissecting the DNA binding landscape and gene regulatory network of p63 and p53. Elife. 2020;9. Epub 2020/12/03. doi: 10.7554/eLife.63266 ; PubMed Central PMCID: PMC7735755. 33263276 PMC7735755

[80] Baergen RN, Warren CD, Isacson C, Ellenson LH. Early uterine serous carcinoma: clonal origin of extrauterine disease. Int J Gynecol Pathol. 2001;20(3):214–9. Epub 2001/07/11. doi: 10.1097/00004347-200107000-00002 . 11444195

[81] Cuevas IC, Sahoo SS, Kumar A, Zhang H, Westcott J, Aguilar M, et al. Fbxw7 is a driver of uterine carcinosarcoma by promoting epithelial-mesenchymal transition. Proc Natl Acad Sci U S A. 2019;116(51):25880–90. Epub 2019/11/28. doi: 10.1073/pnas.1911310116 ; PubMed Central PMCID: PMC6926017. 31772025 PMC6926017

[82] Hajkova N, Ticha I, Hojny J, Nemejcova K, Bartu M, Michalkova R, et al. Synchronous endometrioid endometrial and ovarian carcinomas are biologically related: A clinico-pathological and molecular (next generation sequencing) study of 22 cases. Oncol Lett. 2019;17(2):2207–14. Epub 2019/01/25. doi: 10.3892/ol.2018.9855 ; PubMed Central PMCID: PMC6341770. 30675285 PMC6341770

[83] Yan HB, Wang XF, Zhang Q, Tang ZQ, Jiang YH, Fan HZ, et al. Reduced expression of the chromatin remodeling gene ARID1A enhances gastric cancer cell migration and invasion via downregulation of E-cadherin transcription. Carcinogenesis. 2014;35(4):867–76. Epub 2013/12/03. doi: 10.1093/carcin/bgt398 . 24293408

[84] He F, Li J, Xu J, Zhang S, Xu Y, Zhao W, et al. Decreased expression of ARID1A associates with poor prognosis and promotes metastases of hepatocellular carcinoma. J Exp Clin Cancer Res. 2015;34:47. Epub 2015/05/16. doi: 10.1186/s13046-015-0164-3 ; PubMed Central PMCID: PMC4440314. 25975202 PMC4440314

[85] Zhang L, Wang C, Yu S, Jia C, Yan J, Lu Z, et al. Loss of ARID1A Expression Correlates With Tumor Differentiation and Tumor Progression Stage in Pancreatic Ductal Adenocarcinoma. Technol Cancer Res Treat. 2018;17:1533034618754475. Epub 2018/03/01. doi: 10.1177/1533034618754475 ; PubMed Central PMCID: PMC5833159. 29486633 PMC5833159

[86] Yang Y, Wang X, Yang J, Duan J, Wu Z, Yang F, et al. Loss of ARID1A promotes proliferation, migration and invasion via the Akt signaling pathway in NPC. Cancer Manag Res. 2019;11:4931–46. Epub 2019/06/20. doi: 10.2147/CMAR.S207329 ; PubMed Central PMCID: PMC6549766. 31213911 PMC6549766

[87] Aloni-Grinstein R, Shetzer Y, Kaufman T, Rotter V. p53: the barrier to cancer stem cell formation. FEBS Lett. 2014;588(16):2580–9. Epub 2014/02/25. doi: 10.1016/j.febslet.2014.02.011 . 24560790

[88] Powell E, Piwnica-Worms D, Piwnica-Worms H. Contribution of p53 to metastasis. Cancer Discov. 2014;4(4):405–14. Epub 2014/03/25. doi: 10.1158/2159-8290.CD-13-0136 ; PubMed Central PMCID: PMC4063123. 24658082 PMC4063123

[89] Daikoku T, Hirota Y, Tranguch S, Joshi AR, DeMayo FJ, Lydon JP, et al. Conditional loss of uterine Pten unfailingly and rapidly induces endometrial cancer in mice. Cancer Res. 2008;68(14):5619–27. Epub 2008/07/18. doi: 10.1158/0008-5472.CAN-08-1274 ; PubMed Central PMCID: PMC2824329. 18632614 PMC2824329

[90] Wild PJ, Ikenberg K, Fuchs TJ, Rechsteiner M, Georgiev S, Fankhauser N, et al. p53 suppresses type II endometrial carcinomas in mice and governs endometrial tumour aggressiveness in humans. EMBO Mol Med. 2012;4(8):808–24. Epub 2012/06/09. doi: 10.1002/emmm.201101063 ; PubMed Central PMCID: PMC3494078. 22678923 PMC3494078

[91] Akbay EA, Pena CG, Ruder D, Michel JA, Nakada Y, Pathak S, et al. Cooperation between p53 and the telomere-protecting shelterin component Pot1a in endometrial carcinogenesis. Oncogene. 2013;32(17):2211–9. Epub 2012/06/13. doi: 10.1038/onc.2012.232 ; PubMed Central PMCID: PMC3636499. 22689059 PMC3636499

[92] Guo H, Kong W, Zhang L, Han J, Clark LH, Yin Y, et al. Reversal of obesity-driven aggressiveness of endometrial cancer by metformin. Am J Cancer Res. 2019;9(10):2170–93. Epub 2019/11/14. ; PubMed Central PMCID: PMC6834476. 31720081 PMC6834476

[93] Coppe JP, Desprez PY, Krtolica A, Campisi J. The senescence-associated secretory phenotype: the dark side of tumor suppression. Annu Rev Pathol. 2010;5:99–118. Epub 2010/01/19. doi: 10.1146/annurev-pathol-121808-102144 ; PubMed Central PMCID: PMC4166495. 20078217 PMC4166495

[94] Lozano G. Mouse models of p53 functions. Cold Spring Harb Perspect Biol. 2010;2(4):a001115. Epub 2010/05/11. doi: 10.1101/cshperspect.a001115 ; PubMed Central PMCID: PMC2845198. 20452944 PMC2845198

[95] Schultheis AM, Martelotto LG, De Filippo MR, Piscuglio S, Ng CK, Hussein YR, et al. TP53 Mutational Spectrum in Endometrioid and Serous Endometrial Cancers. Int J Gynecol Pathol. 2016;35(4):289–300. Epub 2015/11/12. doi: 10.1097/PGP.0000000000000243 ; PubMed Central PMCID: PMC5087968. 26556035 PMC5087968

[96] Kurz L, Miklyaeva A, Skowron MA, Overbeck N, Poschmann G, Becker T, et al. ARID1A Regulates Transcription and the Epigenetic Landscape via POLE and DMAP1 while ARID1A Deficiency or Pharmacological Inhibition Sensitizes Germ Cell Tumor Cells to ATR Inhibition. Cancers (Basel). 2020;12(4). Epub 2020/04/11. doi: 10.3390/cancers12040905 ; PubMed Central PMCID: PMC7226530. 32272809 PMC7226530

[97] Kaufmann O, Fietze E, Mengs J, Dietel M. Value of p63 and cytokeratin 5/6 as immunohistochemical markers for the differential diagnosis of poorly differentiated and undifferentiated carcinomas. Am J Clin Pathol. 2001;116(6):823–30. Epub 2002/01/05. doi: 10.1309/21TW-2NDG-JRK4-PFJX . 11764070

[98] Blanco LZ Jr., Heagley DE, Lee JC, Gown AM, Gattuso P, Rotmensch J, et al. Immunohistochemical characterization of squamous differentiation and morular metaplasia in uterine endometrioid adenocarcinoma. Int J Gynecol Pathol. 2013;32(3):283–92. Epub 2013/03/23. doi: 10.1097/PGP.0b013e31826129e1 . 23518912

[99] Zaino RJ, Kurman RJ. Squamous differentiation in carcinoma of the endometrium: a critical appraisal of adenoacanthoma and adenosquamous carcinoma. Semin Diagn Pathol. 1988;5(2):154–71. Epub 1988/05/01. . 3041509

[100] Andrade DAP, da Silva VD, Matsushita GM, de Lima MA, Vieira MA, Andrade C, et al. Squamous differentiation portends poor prognosis in low and intermediate-risk endometrioid endometrial cancer. PLoS One. 2019;14(10):e0220086. Epub 2019/10/11. doi: 10.1371/journal.pone.0220086 ; PubMed Central PMCID: PMC6786591. 31600211 PMC6786591

[101] Shen J, Yao L, Lin YG, DeMayo FJ, Lydon JP, Dubeau L, et al. Glucose-regulated protein 94 deficiency induces squamous cell metaplasia and suppresses PTEN-null driven endometrial epithelial tumor development. Oncotarget. 2016;7(12):14885–97. Epub 2016/02/26. doi: 10.18632/oncotarget.7450 ; PubMed Central PMCID: PMC4924759. 26910913 PMC4924759

[102] Luo Q, Wu X, Chang W, Zhao P, Nan Y, Zhu X, et al. ARID1A prevents squamous cell carcinoma initiation and chemoresistance by antagonizing pRb/E2F1/c-Myc-mediated cancer stemness. Cell Death Differ. 2020;27(6):1981–97. Epub 2019/12/14. doi: 10.1038/s41418-019-0475-6 ; PubMed Central PMCID: PMC7244577. 31831874 PMC7244577

[103] Achenbach F, Rose M, Ortiz-Bruchle N, Seillier L, Knuchel R, Weyerer V, et al. SWI/SNF Alterations in Squamous Bladder Cancers. Genes (Basel). 2020;11(11). Epub 2020/11/25. doi: 10.3390/genes11111368 ; PubMed Central PMCID: PMC7699259. 33227989 PMC7699259

[104] Wang X, Praca MSL, Wendel JRH, Emerson RE, DeMayo FJ, Lydon JP, et al. Vaginal Squamous Cell Carcinoma Develops in Mice with Conditional Arid1a Loss and Gain of Oncogenic Kras Driven by Progesterone Receptor Cre. Am J Pathol. 2021;191(7):1281–91. Epub 2021/04/22. doi: 10.1016/j.ajpath.2021.03.013 ; PubMed Central PMCID: PMC8261476. 33882289 PMC8261476

[105] Jeong JW, Lee HS, Franco HL, Broaddus RR, Taketo MM, Tsai SY, et al. beta-catenin mediates glandular formation and dysregulation of beta-catenin induces hyperplasia formation in the murine uterus. Oncogene. 2009;28(1):31–40. Epub 2008/09/23. doi: 10.1038/onc.2008.363 ; PubMed Central PMCID: PMC2646831. 18806829 PMC2646831

[106] O’Connell JT, Mutter GL, Cviko A, Nucci M, Quade BJ, Kozakewich HP, et al. Identification of a basal/reserve cell immunophenotype in benign and neoplastic endometrium: a study with the p53 homologue p63. Gynecol Oncol. 2001;80(1):30–6. Epub 2001/01/04. doi: 10.1006/gyno.2000.6026 . 11136566

[107] Rubel CA, Wu SP, Lin L, Wang T, Lanz RB, Li X, et al. A Gata2-Dependent Transcription Network Regulates Uterine Progesterone Responsiveness and Endometrial Function. Cell Rep. 2016;17(5):1414–25. Epub 2016/10/27. doi: 10.1016/j.celrep.2016.09.093 ; PubMed Central PMCID: PMC5084852. 27783953 PMC5084852

[108] Wang X, Li X, Wang T, Wu SP, Jeong JW, Kim TH, et al. SOX17 regulates uterine epithelial-stromal cross-talk acting via a distal enhancer upstream of Ihh. Nat Commun. 2018;9(1):4421. Epub 2018/10/26. doi: 10.1038/s41467-018-06652-w ; PubMed Central PMCID: PMC6200785. 30356064 PMC6200785

[109] Gu X, Coates PJ, Boldrup L, Nylander K. p63 contributes to cell invasion and migration in squamous cell carcinoma of the head and neck. Cancer Lett. 2008;263(1):26–34. Epub 2008/01/16. doi: 10.1016/j.canlet.2007.12.011 . 18194839

[110] Zilfou JT, Lowe SW. Tumor suppressive functions of p53. Cold Spring Harb Perspect Biol. 2009;1(5):a001883. Epub 2010/01/13. doi: 10.1101/cshperspect.a001883 ; PubMed Central PMCID: PMC2773645. 20066118 PMC2773645

[111] Luo B, Cheung HW, Subramanian A, Sharifnia T, Okamoto M, Yang X, et al. Highly parallel identification of essential genes in cancer cells. Proc Natl Acad Sci U S A. 2008;105(51):20380–5. Epub 2008/12/19. doi: 10.1073/pnas.0810485105 ; PubMed Central PMCID: PMC2629277. 19091943 PMC2629277

[112] Hiramatsu Y, Fukuda A, Ogawa S, Goto N, Ikuta K, Tsuda M, et al. Arid1a is essential for intestinal stem cells through Sox9 regulation. Proc Natl Acad Sci U S A. 2019;116(5):1704–13. Epub 2019/01/13. doi: 10.1073/pnas.1804858116 ; PubMed Central PMCID: PMC6358682. 30635419 PMC6358682

[113] Zhao B, Lin J, Rong L, Wu S, Deng Z, Fatkhutdinov N, et al. ARID1A promotes genomic stability through protecting telomere cohesion. Nat Commun. 2019;10(1):4067. Epub 2019/09/08. doi: 10.1038/s41467-019-12037-4 ; PubMed Central PMCID: PMC6731242. 31492885 PMC6731242

[114] Rocco JW, Leong CO, Kuperwasser N, DeYoung MP, Ellisen LW. p63 mediates survival in squamous cell carcinoma by suppression of p73-dependent apoptosis. Cancer Cell. 2006;9(1):45–56. Epub 2006/01/18. doi: 10.1016/j.ccr.2005.12.013 . 16413471

[115] Aubrey BJ, Kelly GL, Janic A, Herold MJ, Strasser A. How does p53 induce apoptosis and how does this relate to p53-mediated tumour suppression? Cell Death Differ. 2018;25(1):104–13. Epub 2017/11/18. doi: 10.1038/cdd.2017.169 ; PubMed Central PMCID: PMC5729529. 29149101 PMC5729529

[116] Sano R, Reed JC. ER stress-induced cell death mechanisms. Biochim Biophys Acta. 2013;1833(12):3460–70. Epub 2013/07/16. doi: 10.1016/j.bbamcr.2013.06.028 ; PubMed Central PMCID: PMC3834229. 23850759 PMC3834229

[117] Gonzalez-Quiroz M, Blondel A, Sagredo A, Hetz C, Chevet E, Pedeux R. When Endoplasmic Reticulum Proteostasis Meets the DNA Damage Response. Trends Cell Biol. 2020;30(11):881–91. Epub 2020/10/11. doi: 10.1016/j.tcb.2020.09.002 . 33036871

[118] Suryo Rahmanto Y, Shen W, Shi X, Chen X, Yu Y, Yu ZC, et al. Inactivation of Arid1a in the endometrium is associated with endometrioid tumorigenesis through transcriptional reprogramming. Nat Commun. 2020;11(1):2717. Epub 2020/06/03. doi: 10.1038/s41467-020-16416-0 ; PubMed Central PMCID: PMC7264300. 32483112 PMC7264300

[119] Berglind H, Pawitan Y, Kato S, Ishioka C, Soussi T. Analysis of p53 mutation status in human cancer cell lines: a paradigm for cell line cross-contamination. Cancer Biol Ther. 2008;7(5):699–708. Epub 2008/02/16. doi: 10.4161/cbt.7.5.5712 . 18277095

[120] Harvey DM, Levine AJ. p53 alteration is a common event in the spontaneous immortalization of primary BALB/c murine embryo fibroblasts. Genes Dev. 1991;5(12B):2375–85. Epub 1991/12/01. doi: 10.1101/gad.5.12b.2375 . 1752433

[121] Ali SH, DeCaprio JA. Cellular transformation by SV40 large T antigen: interaction with host proteins. Semin Cancer Biol. 2001;11(1):15–23. Epub 2001/03/13. doi: 10.1006/scbi.2000.0342 . 11243895

[122] Ghandi M, Huang FW, Jane-Valbuena J, Kryukov GV, Lo CC, McDonald ER, 3rd, et al. Next-generation characterization of the Cancer Cell Line Encyclopedia. Nature. 2019;569(7757):503–8. Epub 2019/05/10. doi: 10.1038/s41586-019-1186-3 ; PubMed Central PMCID: PMC6697103. 31068700 PMC6697103

[123] Dobin A, Davis CA, Schlesinger F, Drenkow J, Zaleski C, Jha S, et al. STAR: ultrafast universal RNA-seq aligner. Bioinformatics. 2013;29(1):15–21. Epub 2012/10/30. doi: 10.1093/bioinformatics/bts635 ; PubMed Central PMCID: PMC3530905. 23104886 PMC3530905

[124] Love MI, Huber W, Anders S. Moderated estimation of fold change and dispersion for RNA-seq data with DESeq2. Genome Biol. 2014;15(12):550. Epub 2014/12/18. doi: 10.1186/s13059-014-0550-8 ; PubMed Central PMCID: PMC4302049. 25516281 PMC4302049

[125] Ignatiadis N, Klaus B, Zaugg JB, Huber W. Data-driven hypothesis weighting increases detection power in genome-scale multiple testing. Nat Methods. 2016;13(7):577–80. Epub 2016/05/31. doi: 10.1038/nmeth.3885 ; PubMed Central PMCID: PMC4930141. 27240256 PMC4930141

[126] Martin M. Cutadapt removes adapter sequences from high-throughput sequencing reads. EMBnet J. 2011;17:10–2.

[127] Andrews S. FastQC: A Quality Control Tool for High Throughput Sequence Data. 2010. Available from: http://www.bioinformatics.babraham.ac.uk/projects/fastqc.

[128] Langmead B, Salzberg SL. Fast gapped-read alignment with Bowtie 2. Nat Methods. 2012;9(4):357–9. Epub 2012/03/06. doi: 10.1038/nmeth.1923 ; PubMed Central PMCID: PMC3322381. 22388286 PMC3322381

[129] Li H, Handsaker B, Wysoker A, Fennell T, Ruan J, Homer N, et al. The Sequence Alignment/Map format and SAMtools. Bioinformatics. 2009;25(16):2078–9. Epub 2009/06/10. doi: 10.1093/bioinformatics/btp352 ; PubMed Central PMCID: PMC2723002. 19505943 PMC2723002

[130] Zhang Y, Liu T, Meyer CA, Eeckhoute J, Johnson DS, Bernstein BE, et al. Model-based analysis of ChIP-Seq (MACS). Genome Biol. 2008;9(9):R137. Epub 2008/09/19. doi: 10.1186/gb-2008-9-9-r137 ; PubMed Central PMCID: PMC2592715. 18798982 PMC2592715

[131] Amemiya HM, Kundaje A, Boyle AP. The ENCODE Blacklist: Identification of Problematic Regions of the Genome. Sci Rep. 2019;9(1):9354. Epub 2019/06/30. doi: 10.1038/s41598-019-45839-z ; PubMed Central PMCID: PMC6597582. 31249361 PMC6597582

[132] Quinlan AR, Hall IM. BEDTools: a flexible suite of utilities for comparing genomic features. Bioinformatics. 2010;26(6):841–2. Epub 2010/01/30. doi: 10.1093/bioinformatics/btq033 ; PubMed Central PMCID: PMC2832824. 20110278 PMC2832824

[133] Heinz S, Benner C, Spann N, Bertolino E, Lin YC, Laslo P, et al. Simple combinations of lineage-determining transcription factors prime cis-regulatory elements required for macrophage and B cell identities. Mol Cell. 2010;38(4):576–89. Epub 2010/06/02. doi: 10.1016/j.molcel.2010.05.004 ; PubMed Central PMCID: PMC2898526. 20513432 PMC2898526

[134] Lun AT, Smyth GK. csaw: a Bioconductor package for differential binding analysis of ChIP-seq data using sliding windows. Nucleic Acids Res. 2016;44(5):e45. Epub 2015/11/19. doi: 10.1093/nar/gkv1191 ; PubMed Central PMCID: PMC4797262. 26578583 PMC4797262

[135] Robinson JT, Thorvaldsdottir H, Winckler W, Guttman M, Lander ES, Getz G, et al. Integrative genomics viewer. Nat Biotechnol. 2011;29(1):24–6. Epub 2011/01/12. doi: 10.1038/nbt.1754 ; PubMed Central PMCID: PMC3346182. 21221095 PMC3346182

[136] Gao J, Aksoy BA, Dogrusoz U, Dresdner G, Gross B, Sumer SO, et al. Integrative analysis of complex cancer genomics and clinical profiles using the cBioPortal. Sci Signal. 2013;6(269):pl1. Epub 2013/04/04. doi: 10.1126/scisignal.2004088 ; PubMed Central PMCID: PMC4160307. 23550210 PMC4160307

[137] Cerami E, Gao J, Dogrusoz U, Gross BE, Sumer SO, Aksoy BA, et al. The cBio cancer genomics portal: an open platform for exploring multidimensional cancer genomics data. Cancer Discov. 2012;2(5):401–4. Epub 2012/05/17. doi: 10.1158/2159-8290.CD-12-0095 ; PubMed Central PMCID: PMC3956037. 22588877 PMC3956037

[138] Meyers RM, Bryan JG, McFarland JM, Weir BA, Sizemore AE, Xu H, et al. Computational correction of copy number effect improves specificity of CRISPR-Cas9 essentiality screens in cancer cells. Nat Genet. 2017;49(12):1779–84. Epub 2017/10/31. doi: 10.1038/ng.3984 ; PubMed Central PMCID: PMC5709193. 29083409 PMC5709193

[139] Depmap B. DepMap 21Q1 Public. 2020.

[140] Grossman RL, Heath AP, Ferretti V, Varmus HE, Lowy DR, Kibbe WA, et al. Toward a Shared Vision for Cancer Genomic Data. N Engl J Med. 2016;375(12):1109–12. Epub 2016/09/23. doi: 10.1056/NEJMp1607591 ; PubMed Central PMCID: PMC6309165. 27653561 PMC6309165

[141] Li B, Dewey CN. RSEM: accurate transcript quantification from RNA-Seq data with or without a reference genome. BMC Bioinformatics. 2011;12:323. Epub 2011/08/06. doi: 10.1186/1471-2105-12-323 ; PubMed Central PMCID: PMC3163565. 21816040 PMC3163565

[142] Ritchie ME, Phipson B, Wu D, Hu Y, Law CW, Shi W, et al. limma powers differential expression analyses for RNA-sequencing and microarray studies. Nucleic Acids Res. 2015;43(7):e47. Epub 2015/01/22. doi: 10.1093/nar/gkv007 ; PubMed Central PMCID: PMC4402510. 25605792 PMC4402510

[143] Benjamini Y, Drai D, Elmer G, Kafkafi N, Golani I. Controlling the false discovery rate in behavior genetics research. Behav Brain Res. 2001;125(1–2):279–84. Epub 2001/10/30. doi: 10.1016/s0166-4328(01)00297-2 . 11682119

[144] The Gene Ontology Consortium. The Gene Ontology Resource: 20 years and still GOing strong. Nucleic Acids Res. 2019;47(D1):D330–D8. Epub 2018/11/06. doi: 10.1093/nar/gky1055 ; PubMed Central PMCID: PMC6323945. 30395331 PMC6323945

[145] Ashburner M, Ball CA, Blake JA, Botstein D, Butler H, Cherry JM, et al. Gene ontology: tool for the unification of biology. The Gene Ontology Consortium. Nat Genet. 2000;25(1):25–9. Epub 2000/05/10. doi: 10.1038/75556 ; PubMed Central PMCID: PMC3037419. 10802651 PMC3037419

[146] Reich M, Liefeld T, Gould J, Lerner J, Tamayo P, Mesirov JP. GenePattern 2.0. Nat Genet. 2006;38(5):500–1. Epub 2006/04/28. doi: 10.1038/ng0506-500 . 16642009

[147] Yu G, Wang LG, Han Y, He QY. clusterProfiler: an R package for comparing biological themes among gene clusters. OMICS. 2012;16(5):284–7. Epub 2012/03/30. doi: 10.1089/omi.2011.0118 ; PubMed Central PMCID: PMC3339379. 22455463 PMC3339379

[148] Smedley D, Haider S, Ballester B, Holland R, London D, Thorisson G, et al. BioMart—biological queries made easy. BMC Genomics. 2009;10:22. Epub 2009/01/16. doi: 10.1186/1471-2164-10-22 ; PubMed Central PMCID: PMC2649164. 19144180 PMC2649164

[149] Gu Z, Eils R, Schlesner M. Complex heatmaps reveal patterns and correlations in multidimensional genomic data. Bioinformatics. 2016;32(18):2847–9. Epub 2016/05/22. doi: 10.1093/bioinformatics/btw313 . 27207943

[150] Pollard KS, Hubisz MJ, Rosenbloom KR, Siepel A. Detection of nonneutral substitution rates on mammalian phylogenies. Genome Res. 2010;20(1):110–21. Epub 2009/10/28. doi: 10.1101/gr.097857.109 ; PubMed Central PMCID: PMC2798823. 19858363 PMC2798823

[151] Siepel A, Bejerano G, Pedersen JS, Hinrichs AS, Hou M, Rosenbloom K, et al. Evolutionarily conserved elements in vertebrate, insect, worm, and yeast genomes. Genome Res. 2005;15(8):1034–50. Epub 2005/07/19. doi: 10.1101/gr.3715005 ; PubMed Central PMCID: PMC1182216. 16024819 PMC1182216

[152] Wickham H. ggplot2: Elegant Graphics for Data Analysis: Springer-Verlag New York; 2016. 212 p.

[153] R Core Team. R: A language and environment for statistical computing. 2018.

