# Supplementary figures and images for: Co-existing TP53 and ARID1A mutations promote aggressive endometrial tumorigenesis
Source: PLoS Genet. 2021 Dec 23;17(12):e1009986. doi: 10.1371/journal.pgen.1009986 (PMC8741038; doi:10.1371/journal.pgen.1009986)

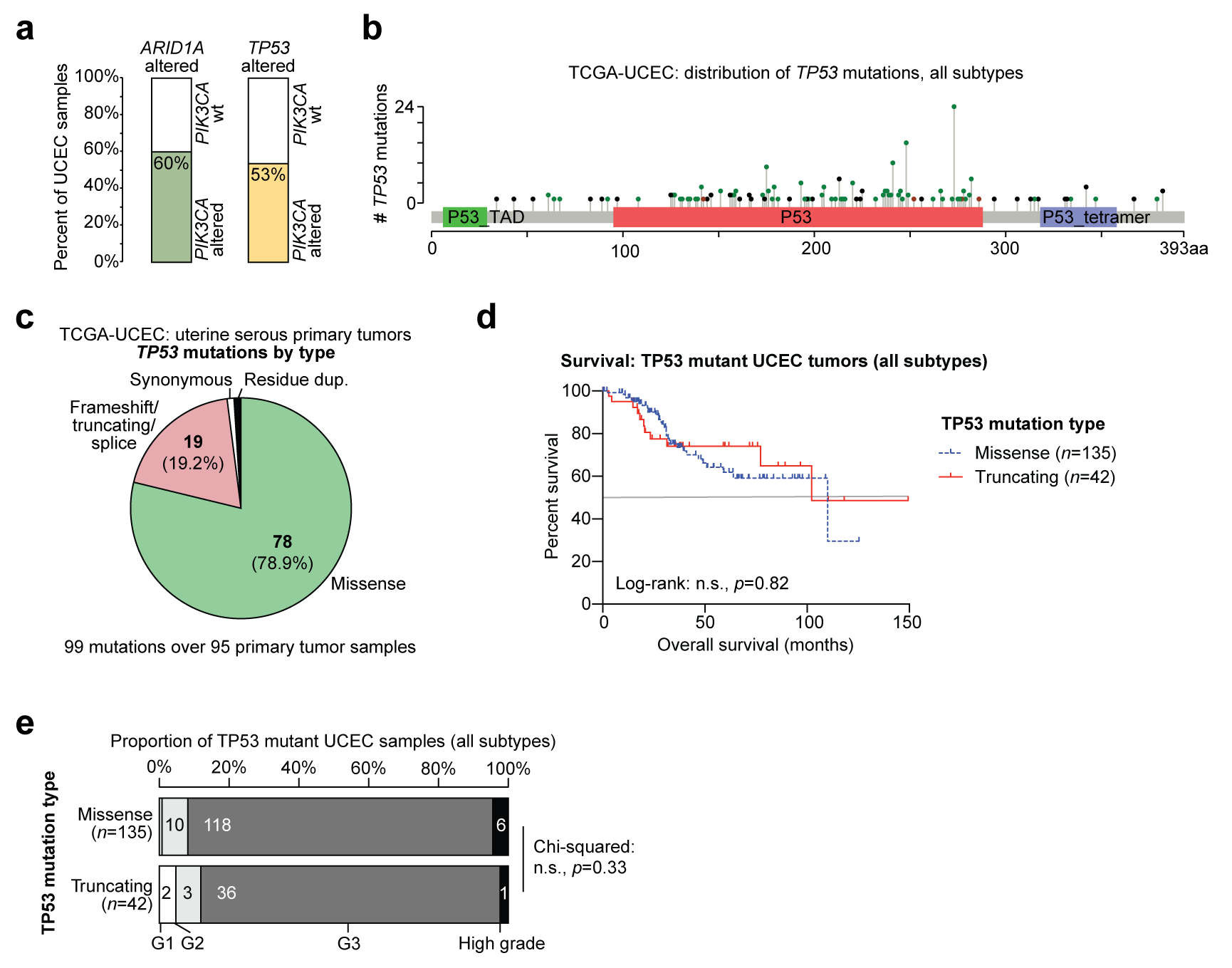

Supplement: S1 Fig — a, PIK3CA co-alteration rate among ARID1A and TP53 altered UCEC tumors. b, Lollipop plot for mutations in TP53 gene across TCGA-UCEC data (Pan-Can cohort). c, Distribution of TP53 mutations by type in TCGA-UCEC serous subtype primary tumors. d, Kaplan-Meier overall survival curves for TP53 mutant UCEC tumors segregated by type of TP53 mutation: missense vs. truncating. Statistic is log-rank test. e, Distribution of tumor grading among TP53 mutant UCEC tumors segregated by type of TP53 mutation: missense vs. truncating. Statistic is chi-squared test. (TIF) [file pgen.1009986.s001.tif]

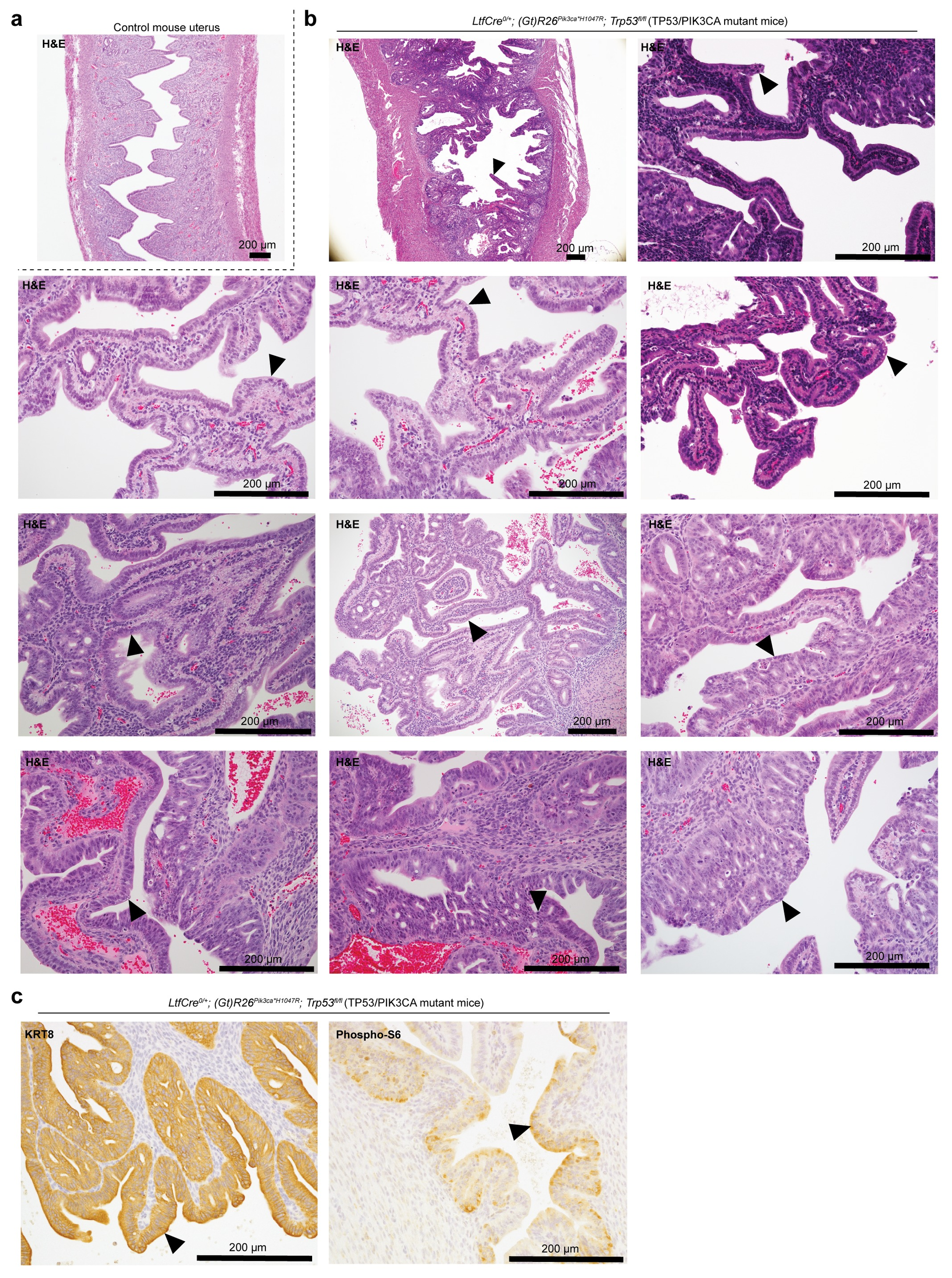

Supplement: S2 Fig — a, Representative low-magnification H&E histology of control mouse uterus. b, Additional representative H&E histology of TP53/PIK3CA mutant uterus (approximately 76-day old) at varying magnifications. Arrowheads depict endometrial epithelia. c, KRT8 (left), a marker of endometrial epithelium, and phospho-S6 (right), a marker of activated PI3K signaling. Arrowheads depict mutant endometrial epithelia. (TIF) [file pgen.1009986.s002.tif]

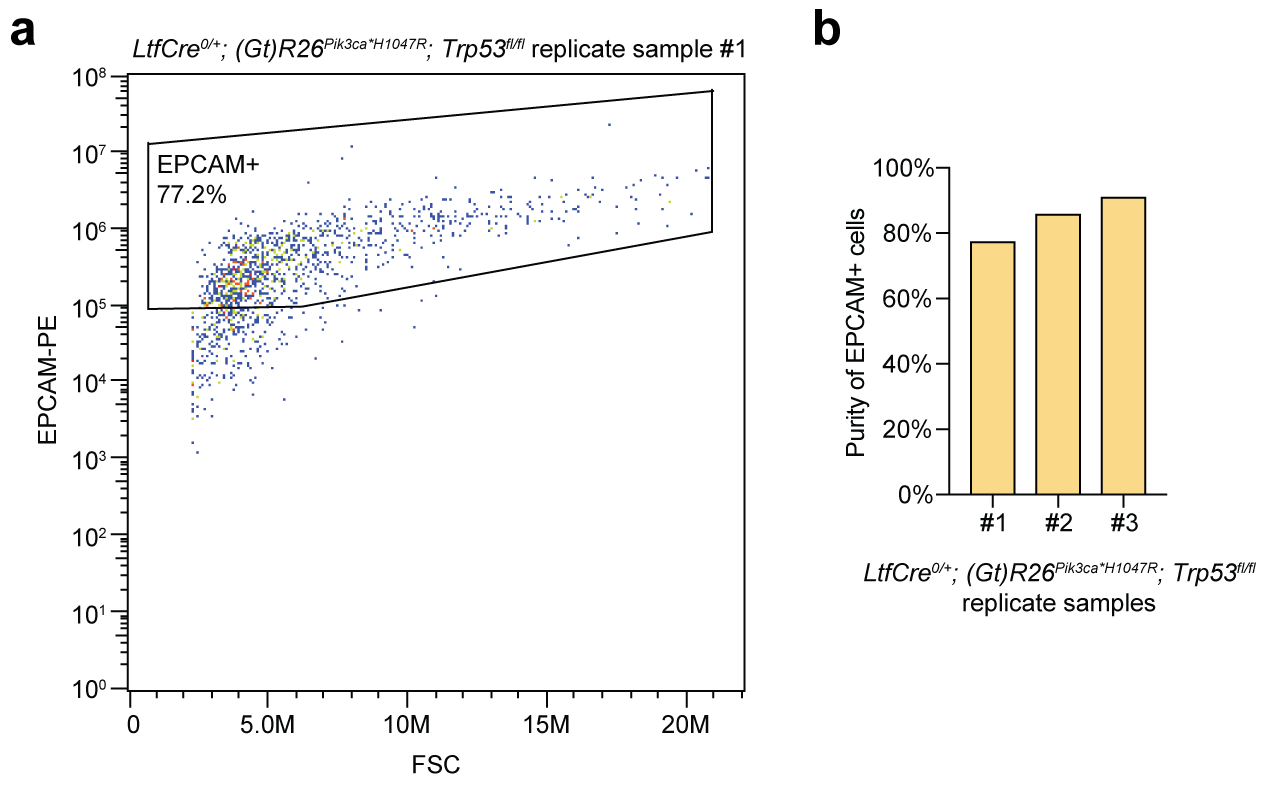

Supplement: S3 Fig — a, Example flow cytometry analysis of EPCAM purity from magnetically sorted LtfCre0/+; (Gt)R26Pik3ca*H1047R; Trp53fl/fl (TP53/PIK3CA mutant) mouse endometrial epithelial cells. b, Purity of EPCAM-isolated cell populations for each sample sequenced by RNA-seq. Mean purity ± SD (%) among sequenced samples was 84.6 ± 6.9. These results are not significantly different from the previously reported control group, 87.7 ± 5.4 (by unpaired, two-tailed t-test). (TIF) [file pgen.1009986.s003.tif]

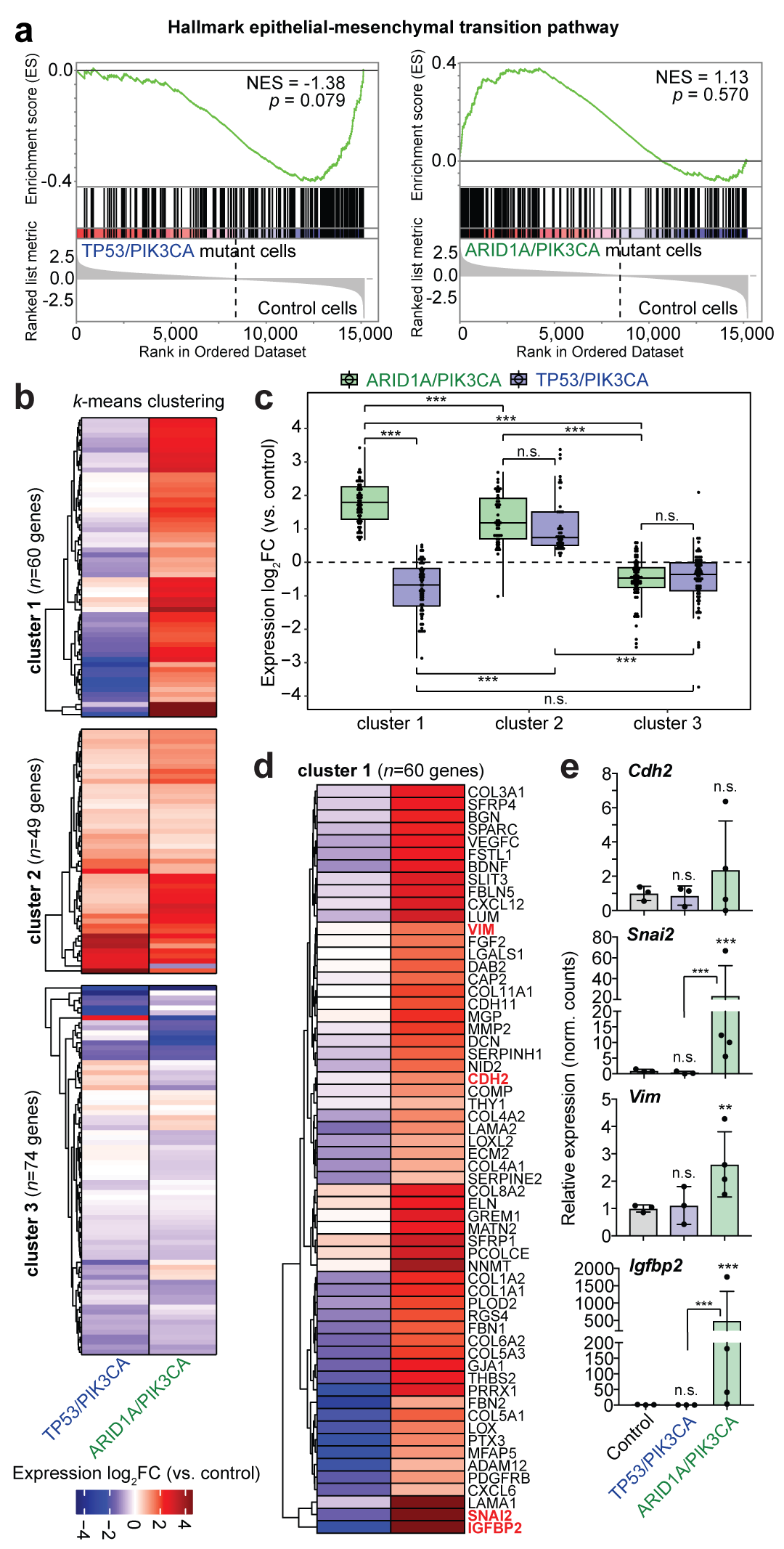

Supplement: S4 Fig — a, Broad GSEA waterfall plots for the Hallmark epithelial-mesenchymal transition (EMT) pathway in cells from each genetic mouse model compared to controls. b, k-means clustering (k = 3) of differential gene expression in TP53/PIK3CA mutant and ARID1A/PIK3CA mutant endometrial epithelial cells compared to controls for 183 mouse orthologs within the Hallmark EMT pathway. Red values indicate gene upregulation in mutant cells, and blue values indicate downregulation. c, Relative expression box-dot plots summarizing gene expression changes in the k clusters for each genetic mouse model compared to control cells. Statistic is unpaired, two-tailed Wilcoxon test. *** p < 0.001. d, Zoom into cluster 1 genes (n = 60) labeled by human ortholog. Red, bolded genes are further displayed in e as a box-dot plot. Statistic is FDR as reported by DESeq2 Wald test: * FDR < 0.05; ** FDR < 0.01; *** FDR < 0.001. (TIF) [file pgen.1009986.s004.tif]

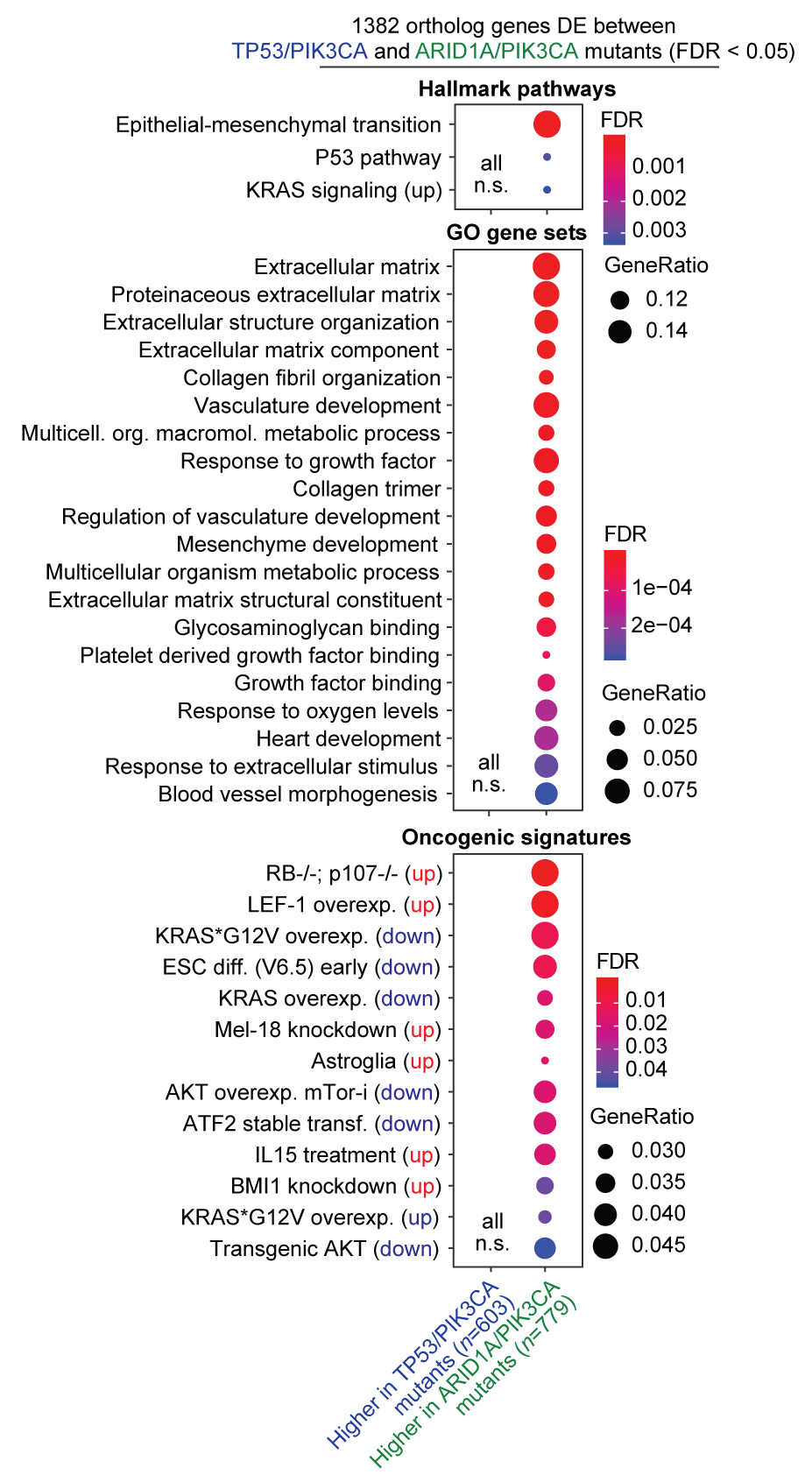

Supplement: S5 Fig — Enrichment for Hallmark pathways, GO Biological Process gene sets, and oncogenic signatures (all retrieved from MSigDB) among genes DE between TP53/PIK3CA mutant vs. ARID1A/PIK3CA mutant endometrial epithelial cells, separated by directionality. (TIF) [file pgen.1009986.s005.tif]

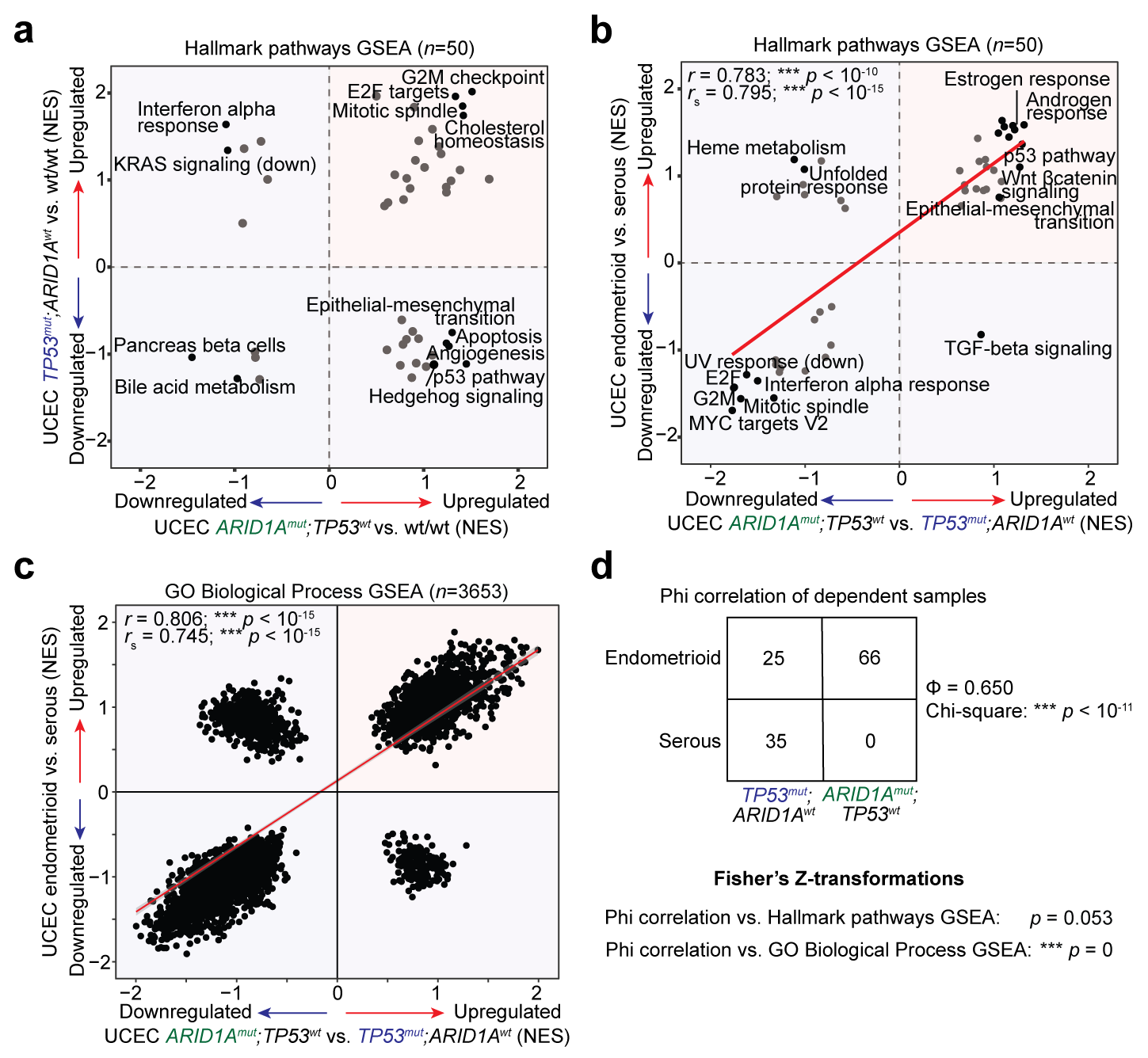

Supplement: S6 Fig — a, Broad GSEA results for Hallmark pathways between TCGA-UCEC tumors: ARID1A mutant / TP53 wild-type vs. wild-type / wild-type compared to TP53 mutant / ARID1A wild-type vs. wild-type / wild-type. b, Broad GSEA results for Hallmark pathways between TCGA-UCEC tumors: ARID1A mutant / TP53 wild-type vs. TP53 mutant / ARID1A wild-type compared to endometrioid vs. serous. Significant correlation of pathway enrichment is observed between genetics and subtype by Pearson (r) and Spearman (rs) correlations. c, same as in b but for GO Biological Process gene sets. d, Top, phi correlation and associated statistic of dependent samples classified as either endometrioid vs. serous histotype and TP53mut/ARID1Awt or ARID1Amut/TP53wt. Bottom, Fisher’s Z-transformations comparing the Pearson correlation coefficients between the phi correlation and GSEA results. (TIF) [file pgen.1009986.s006.tif]

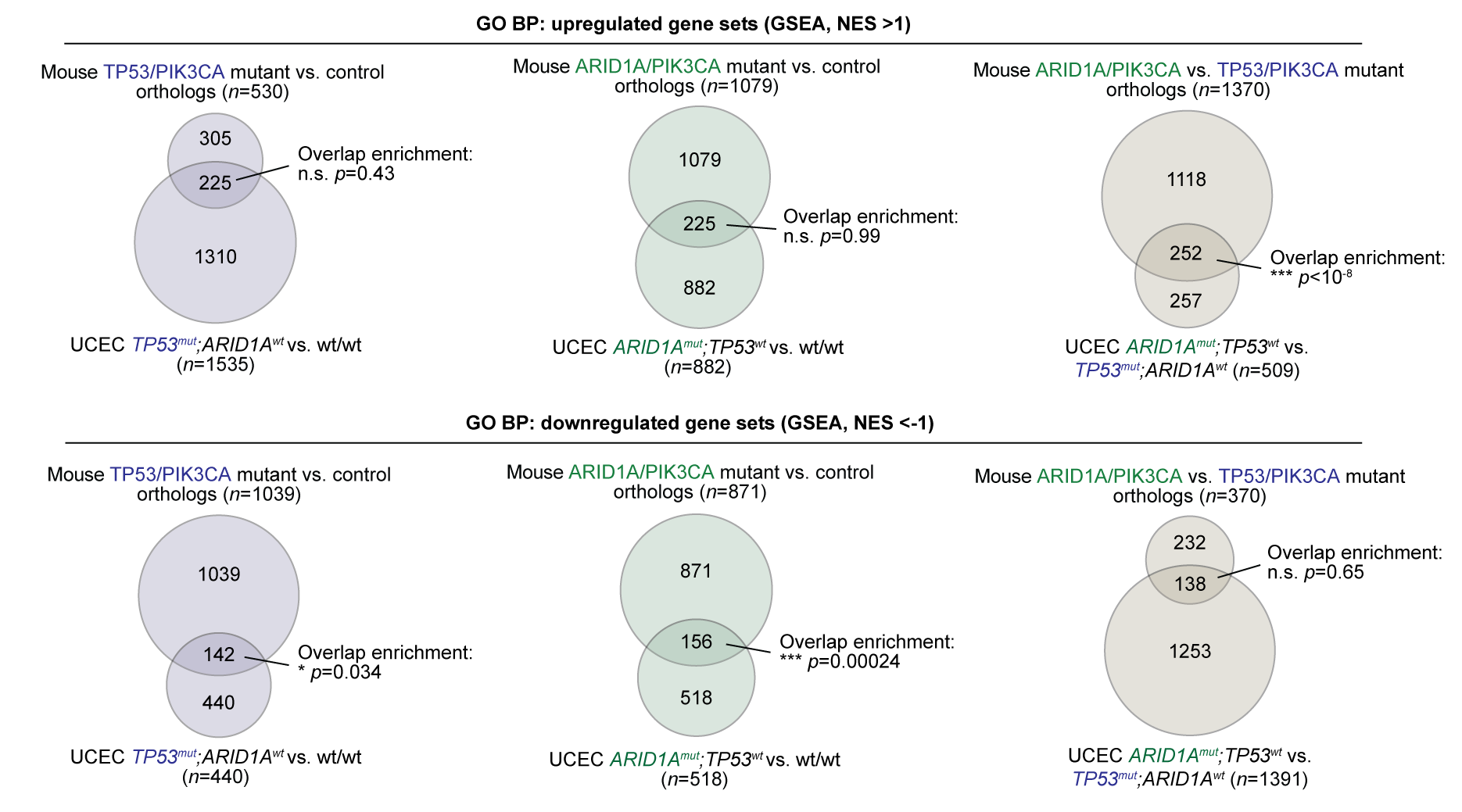

Supplement: S7 Fig — Overlap of enriched gene sets (|NES| > 1) determined in Fig 4 GSEA for various mouse and human genetic comparisons as displayed, further segregated by upregulated vs. downregulated gene sets. Significant overlap indicates that more enriched gene sets were observed in both comparisons than expected by chance alone. Statistic is hypergeometric enrichment. (TIF) [file pgen.1009986.s007.tif]

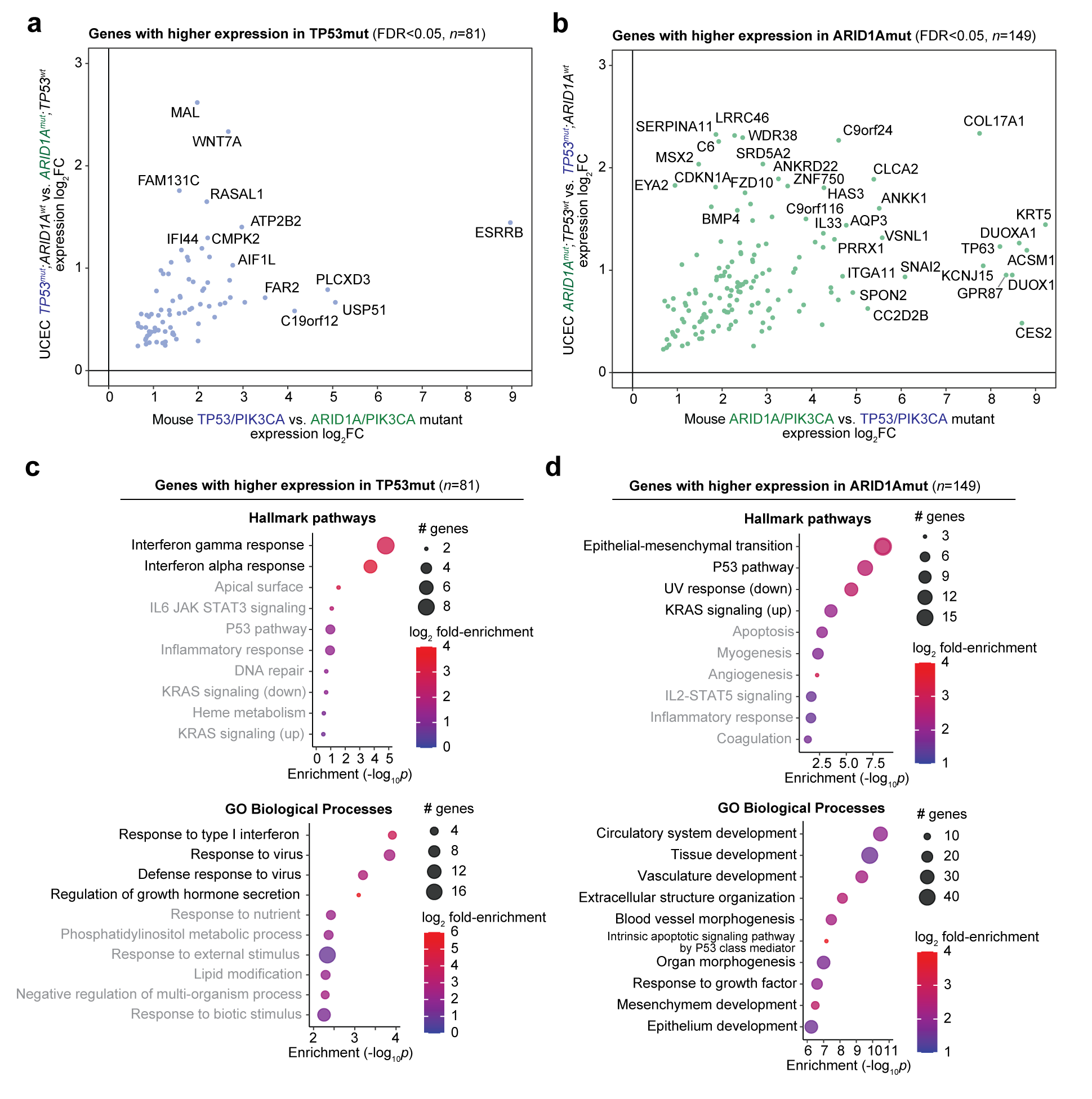

Supplement: S8 Fig — a, 81 genes with significantly higher expression (human: limma FDR < 0.05; mouse: DESeq2 FDR < 0.05) in TP53 mutant samples compared to ARID1A mutants. b, as in a but for 149 genes with higher expression in ARID1A mutant samples compared to TP53 mutants. c, top 10 enriched Hallmark pathways and GO Biological Process gene sets among the 81 genes identified in a. Gray text indicates non-significance (FDR > 0.05). d, as in c but for genes identified in b. (TIF) [file pgen.1009986.s008.tif]

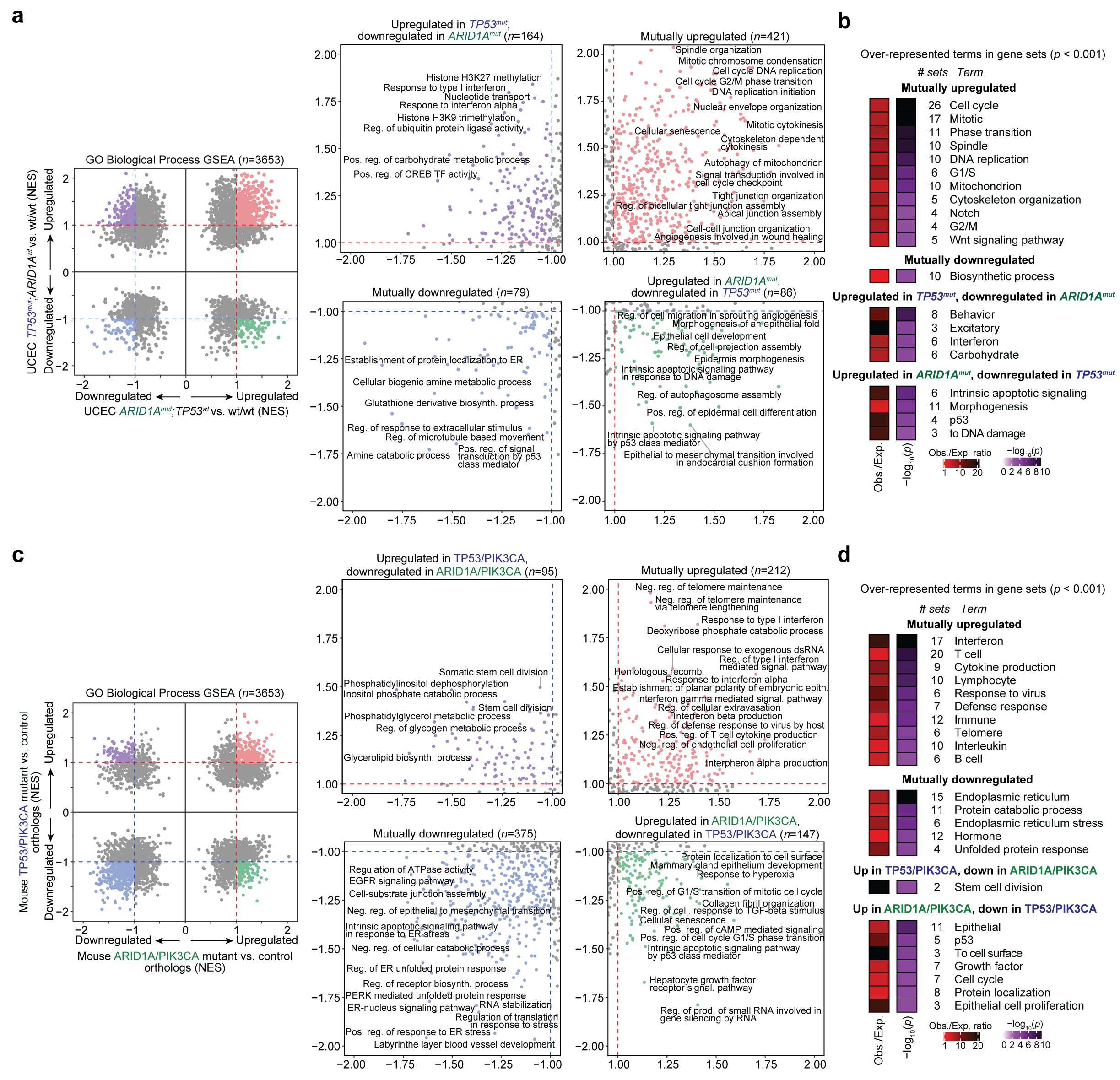

Supplement: S9 Fig — a, Detailed Broad GSEA results for TCGA-UCEC ARID1A mutant / TP53 wild-type vs. wild-type / wild-type compared to TP53 mutant / ARID1A wild-type vs. wild-type / wild-type. Representative examples of highly enriched gene sets are labeled for each quadrant. b, Significantly over-represented terms in enriched gene sets (|NES| > 1) highlighted in a. Statistic is hypergeometric enrichment. See methods for analysis framework. c-d, Same as in a-b but for TP53/PIK3CA mutant vs. control cells compared to ARID1A/PIK3CA mutant vs. control cells. (TIF) [file pgen.1009986.s009.tif]

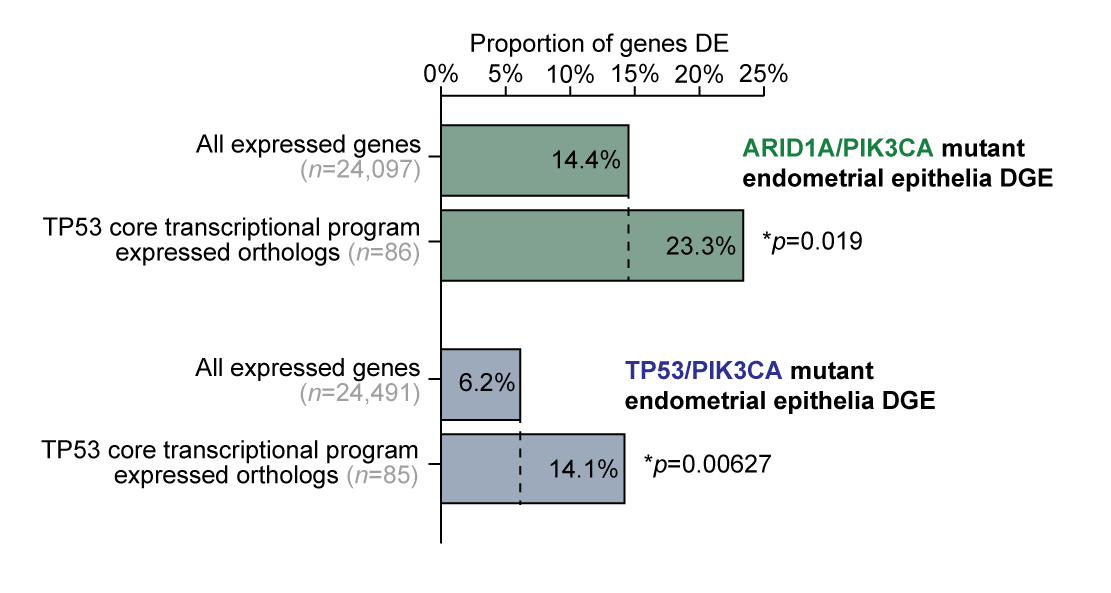

Supplement: S10 Fig — Proportion of genes significantly differentially expressed (DE) in TP53 core transcriptional program gene mouse orthologs compared to all expressed genes, for (top) ARID1A/PIK3CA mutant and (bottom) TP53/PIK3CA mutant endometrial epithelia compared to cells from control mice. Statistic is hypergeometric enrichment test. (TIF) [file pgen.1009986.s010.tif]

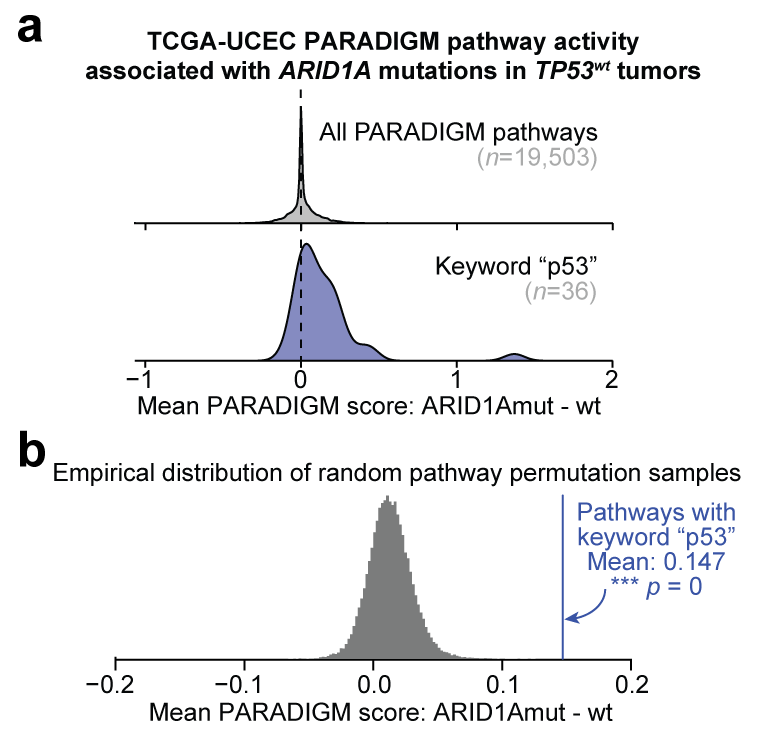

Supplement: S11 Fig — a, Distribution of PARADIGM score differences between ARID1A mutant (n = 174) vs. wild-type (n = 128) TCGA-UCEC tumors, considerate of only TP53 wild-type tumors, as in Fig 5C. Top, all 19,503 measured pathways; bottom, the 36 pathways with keyword “p53”. b, Empirical distribution of mean differences of ARID1A mutant vs. wild-type PARADIGM scores, based on 50,000 samples of 36 random PARADIGM pathways, as in Fig 5D. The blue line represents the mean score difference for the 36 pathways with keyword “p53” with associated permutation statistic. (TIF) [file pgen.1009986.s011.tif]

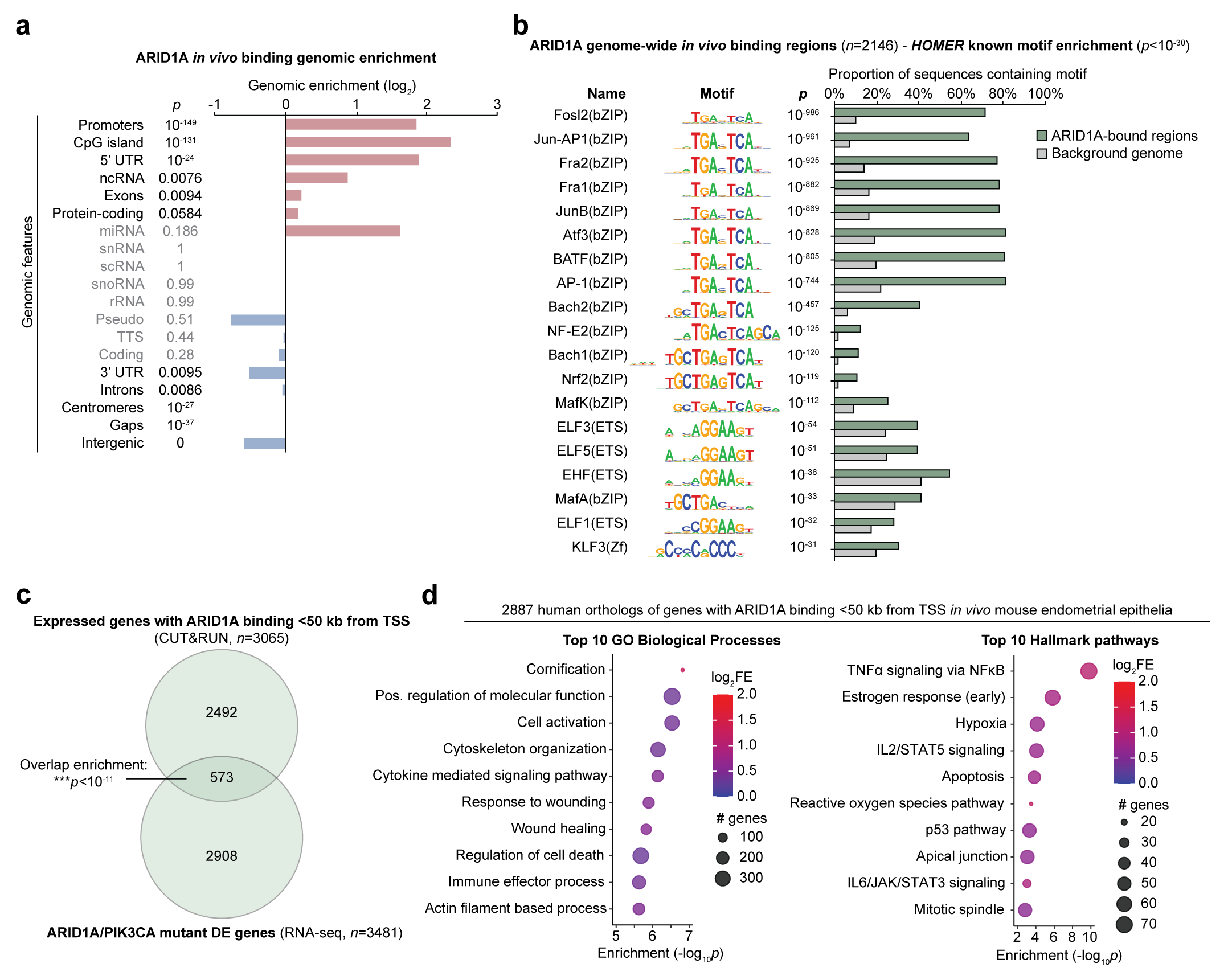

Supplement: S12 Fig — a, Genomic feature enrichment among 2146 ARID1A in vivo binding sites. b, Top significant (p < 10−30) known motifs from HOMER sequence analysis of ARID1A in vivo binding sites compared to the background genome. Motif sequence logos are scaled by information content for each nucleotide base. c, Overlap of ARID1A/PIK3CA mutant DE genes (RNA-seq, FDR < 0.05, n = 3481) and genes with ARID1A binding detected within 50 kb of TSS. d, Top 10 (left) GO Biological Process gene sets and (right) Hallmark pathways enriched among 2887 human orthologs of genes with ARID1A binding within 50 kb from TSS. (TIF) [file pgen.1009986.s012.tif]

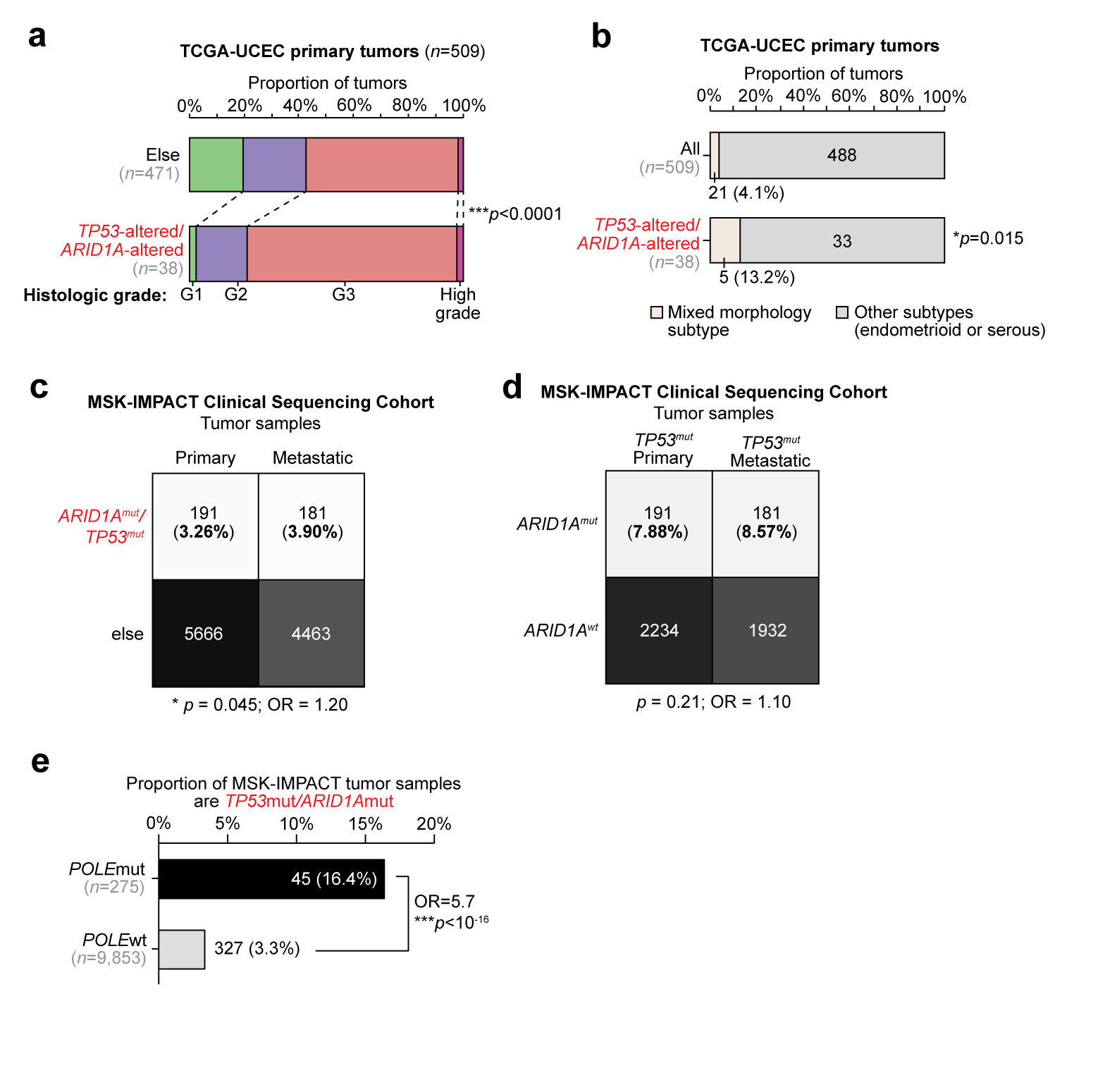

Supplement: S13 Fig — a, Distribution of histologic grading among TCGA-UCEC primary tumors, segregated by TP53/ARID1A co-altered (n = 38) vs. else (n = 471). Statistic is two-tailed Fisher’s exact test. b, Frequency of mixed morphology subtype tumors across all TCGA-UCEC primary tumors (n = 509) compared to specifically TP53/ARID1A co-altered tumors (n = 38). Statistic is hypergeometric enrichment. c, Contingency table of TP53/ARID1A co-mutation rate in all primary vs. metastatic tumors from the MSK-IMPACT Clinical Sequencing Cohort. Statistic is one-tailed Fisher’s exact test. d, Contingency table of ARID1A co-mutation rate in TP53 mutant primary vs. metastatic tumors from MSK-IMPACT as in c. e, TP53/ARID1A co-mutation rates of all MSK-IMPACT tumor samples segregated by presence or absence of POLE mutations. Statistic is two-tailed Fisher’s exact test. (TIF) [file pgen.1009986.s013.tif]

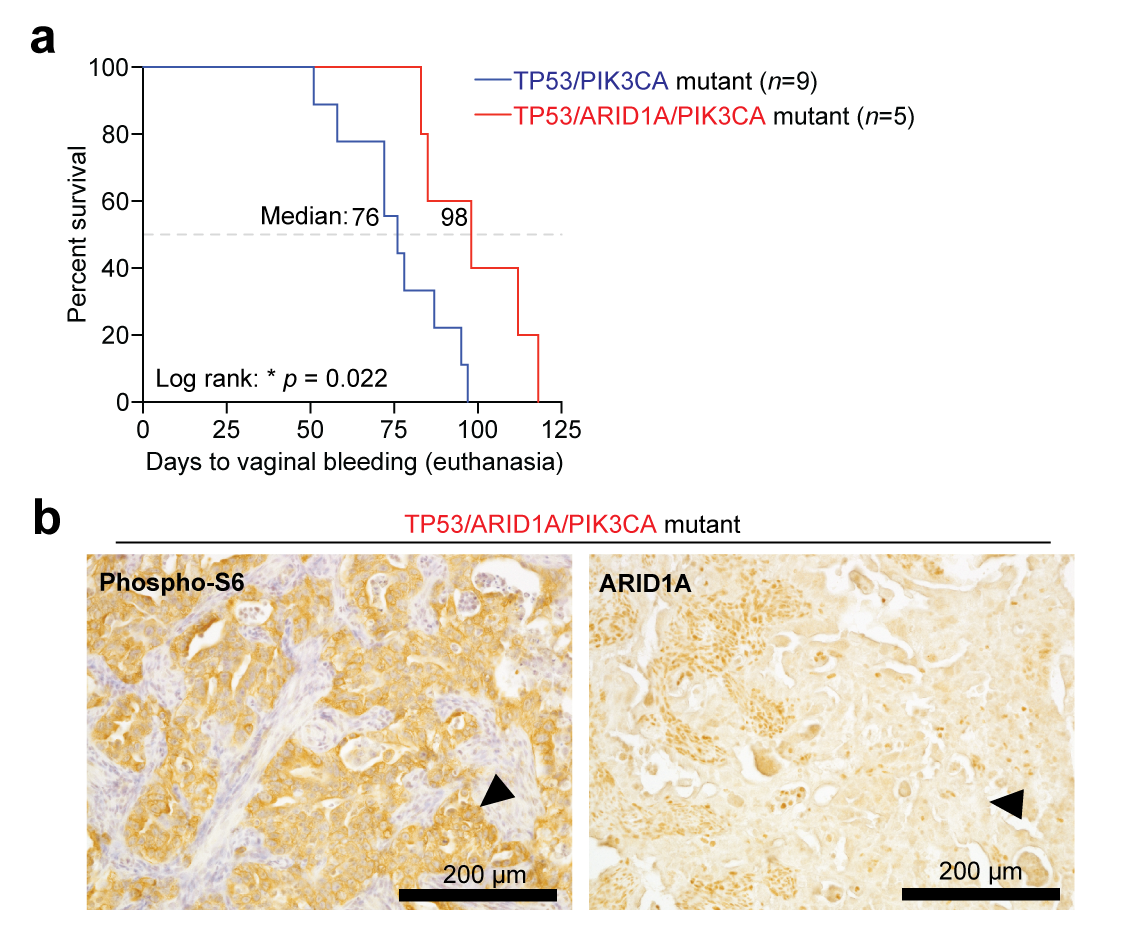

Supplement: S14 Fig — a, Survival curves for LtfCre0/+; (Gt)R26Pik3ca*H1047R; Trp53fl/fl mice with and without an additional Arid1afl/fl allele (TP53/PIK3CA mutant and TP53/ARID1A/PIK3CA mutant mice). Statistic is Cox log-rank test. b, Representative IHC marker analysis in TP53/ARID1A/PIK3CA mutant mice. Left, phospho-S6; right, ARID1A staining. Arrowheads denote mutant endometrial epithelia. (TIF) [file pgen.1009986.s014.tif]

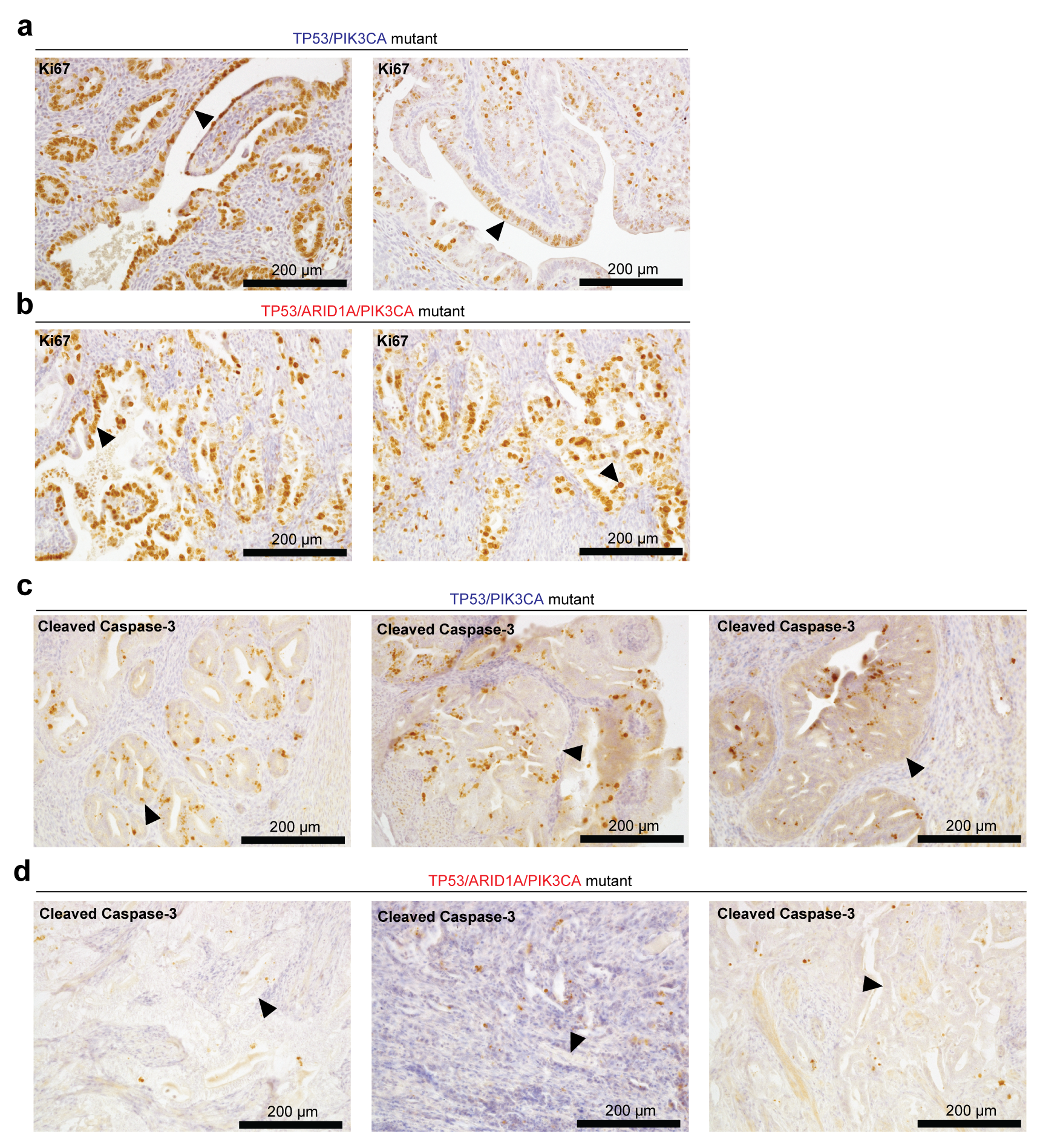

Supplement: S15 Fig — More representative IHC images of Ki67 (proliferation) and cleaved caspase-3 (cell death) in TP53/PIK3CA mutant (a and c) and TP53/ARID1A/PIK3CA mutant (b and d) mouse uterus, respectively. Arrowheads denote mutant endometrial epithelia. (TIF) [file pgen.1009986.s015.tif]

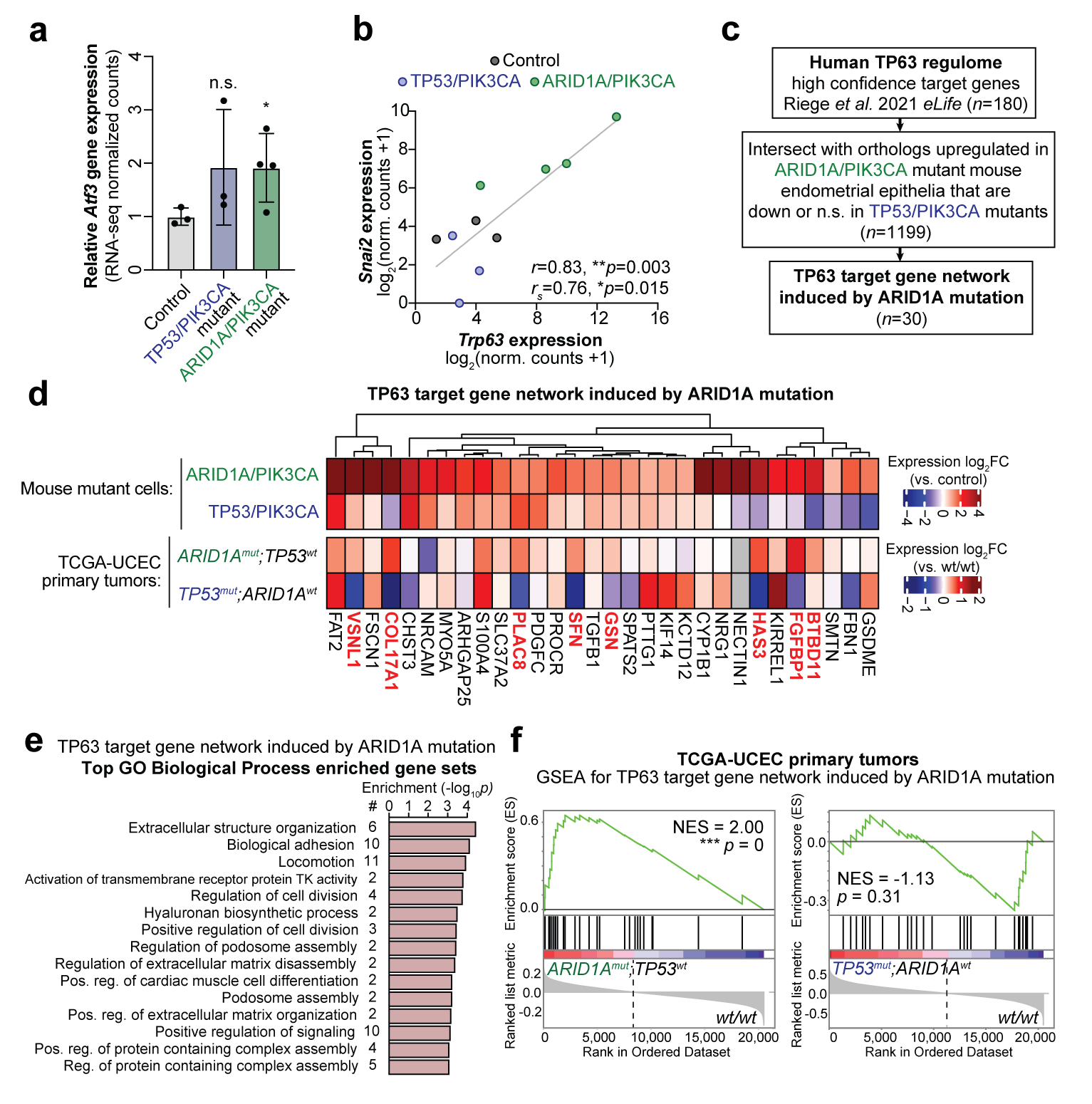

Supplement: S16 Fig — a, Atf3 gene expression (linear) in mutant mouse endometrial epithelial cell RNA-seq data. Statistic is FDR as reported by DESeq2. b, Significant correlation of Trp63 and Snai2 gene expression in experimental mouse endometrial epithelial cells. Expression is quantified as log2(normalized counts + 1). Statistics are Pearson (r) and Spearman (rs) coefficients. c, Workflow for identification of the TP63 target gene network induced by ARID1A mutation (n = 30 genes), beginning with 180 high confidence TP63 target genes defined by Riege et al. [79]. d, RNA-seq relative expression heatmap for genetic mouse models and UCEC primary tumor samples for the TP63 target gene network induced by ARID1A mutation. Red, bolded genes display similar expression patterns in genetic mouse models and human UCEC tumors. e, Top GO Biological Process gene sets enriched (hypergeometric enrichment p < 0.001) in the TP63 target gene network induced by ARID1A mutation. # represents the number of target genes found within each gene set. f, Broad GSEA results for the TP63 target gene network induced by ARID1A mutation among human UCEC primary tumors segregated by ARID1A and TP53 genetic status. Significant enrichment was observed in ARID1Amut/TP53wt vs. wt/wt tumors. (TIF) [file pgen.1009986.s016.tif]

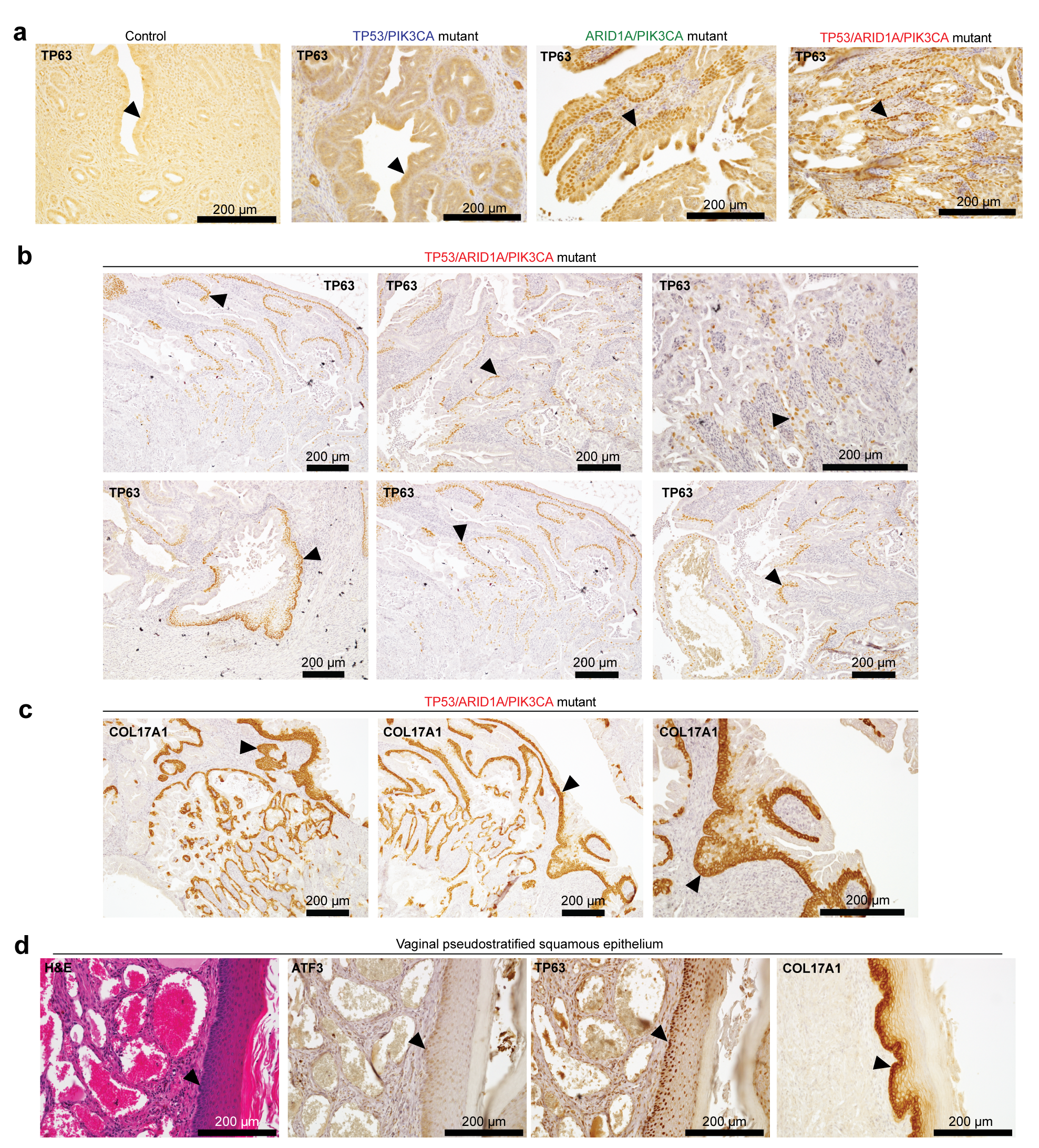

Supplement: S17 Fig — a, Representative uterine TP63 (Cell Signaling) staining in mutant mouse models. Arrowheads denote endometrial epithelium. b-c, Further representative uterine (b) TP63 (GeneTex) and (c) COL17A1 staining in TP53/ARID1A/PIK3CA mutant mice. Arrowheads denote mutant endometrial epithelia. d, Representative H&E and IHC staining for ATF3, TP63 (Cell Signaling), and COL17A1 in vaginal pseudostratified squamous epithelium of wild-type CD-1 mice. Arrowheads denote basal epithelial cells expressing markers. (TIF) [file pgen.1009986.s017.tif]
